# Analysis of the Involvement of Different Ceramide Variants in the Response to Hydroxyurea Stress in Baker's Yeast

**DOI:** 10.1371/journal.pone.0146839

**Published:** 2016-01-19

**Authors:** Po-Wei Chen, Luis L. Fonseca, Yusuf A. Hannun, Eberhard O. Voit

**Affiliations:** 1 Wallace H. Coulter Department of Biomedical Engineering, Georgia Institute of Technology, Atlanta, Georgia, United States of America; 2 The George W. Woodruff School of Mechanical Engineering, Georgia Institute of Technology, Atlanta, Georgia, United States of America; 3 The Cancer Center at Stony Brook Medicine, Stony Brook University, Health Science Center, Stony Brook, New York, United States of America; Louisiana State University Health Sciences Center, UNITED STATES

## Abstract

Sphingolipids have been identified as important signaling compounds in stress responses. However, it is not always clear how different sphingolipid profiles are achieved in a particular stress situation. Here we propose a detailed mass action model, containing 42 dependent variables and 137 reactions, that offers explanations of the roles of variant ceramides species, which differ in the lengths of their fatty acyl chains and their saturation state, in the response to hydroxyurea stress. The simulations demonstrate that the cells manage to achieve hydroxyurea tolerance through a well-coordinated, differential usage of the variant ceramide species. Moreover, the results suggest that key enzymes have different affinities toward saturated and unsaturated fatty acyl chains, which implies that the saturation state affords the cells with an additional mode of regulation that had not been recognized so far. These conclusions from our computational analysis are yet to be validated experimentally.

## Introduction

Sphingolipids actively participate as second messengers in crucial functions of eukaryotic cells. Within the class of sphingolipids, ceramides have been investigated for several decades, but the focus has recently shifted toward their direct involvement in signal transduction, which in mammalian organisms ultimately leads to cell proliferation, differentiation, and apoptosis [1–3]. While the relationships between ceramide species, signaling cascades and gene expressions are gradually becoming clearer, it is still unknown how the concentrations of the various ceramide species are altered by cells in response to stresses. Two reasons for this lack in understanding are that sphingolipid metabolism constitutes a complex, highly regulated pathway system and that consistent metabolic and enzymatic time series measurements are difficult to obtain.

In contrast to mammalian cells, yeast cells are much more easily investigated, and because they are also eukaryotic, they have become valuable model systems for mammalian stress responses. In particular, the metabolism of sphingolipids is highly conserved and quite similar between mammalian cells and baker’s yeast, and it is feasible to measure stress responses more or less directly under a variety of conditions. However, such measurements alone do not reveal the strategies that cells evoke to respond to stresses. Thus, we propose to apply a computational approach that uses experimental data of different ceramide variants and is designed to reveal and characterize patterns of metabolic regulation within the ceramide pathway under hydroxyurea stress.

The reasons for choosing hydroxyurea stress are the following. Hydroxyurea inhibits the enzyme ribonucleotide reductase and thereby decreases or even stalls DNA synthesis [4]. Due to this property, hydroxyurea has been used increasingly as a treatment option for a variety of diseases including HIV infection and AIDS, sickle cell anemia, and myeloproliferative neoplasms [5–8]. In spite of the growing interest in hydroxyurea, details of the mechanisms of action are not fully understood, and it is therefore not surprising that the investigation of cellular responses to hydroxyurea has become a highly interesting topic in biomedical research.

Using *Saccharomyces cerevisiae* as a model organism, the roles of ceramides in signal transduction under hydroxyurea exposure are beginning to become clearer. For instance, the tolerance of yeast cells to hydroxyurea exposure decreases in knockouts of each one of several genes, including isc1 (IPCase) and sur4 (fatty acid elongation), in double gene knockouts such as lag1 and lac1 (ceramide synthase), as well as in strains overexpressing ydc1 (phytoceramidase) [9]. These findings strongly imply the involvement of ceramide in mediating hydroxyurea stress. Further research has suggested a signaling cascade, which starts with an increased concentration of C18:1 phytoceramide (C18:1 PHC, phytoceramide with a C18:1 fatty acyl CoA), which triggers the activation of a sub-domain of Cdc55/PP2A (a regulatory subunit for protein phosphatase 2A) that leads to a decrease in Swe1 (a mitosis inhibitor protein kinase) level, causes dephosphorylation of Clb2-Cdc28 (which activates Cdc28p to promote the cell cycle transition from G2 to M), and ultimately activates the G2/M checkpoint [9, 10].

**Fig 1:**
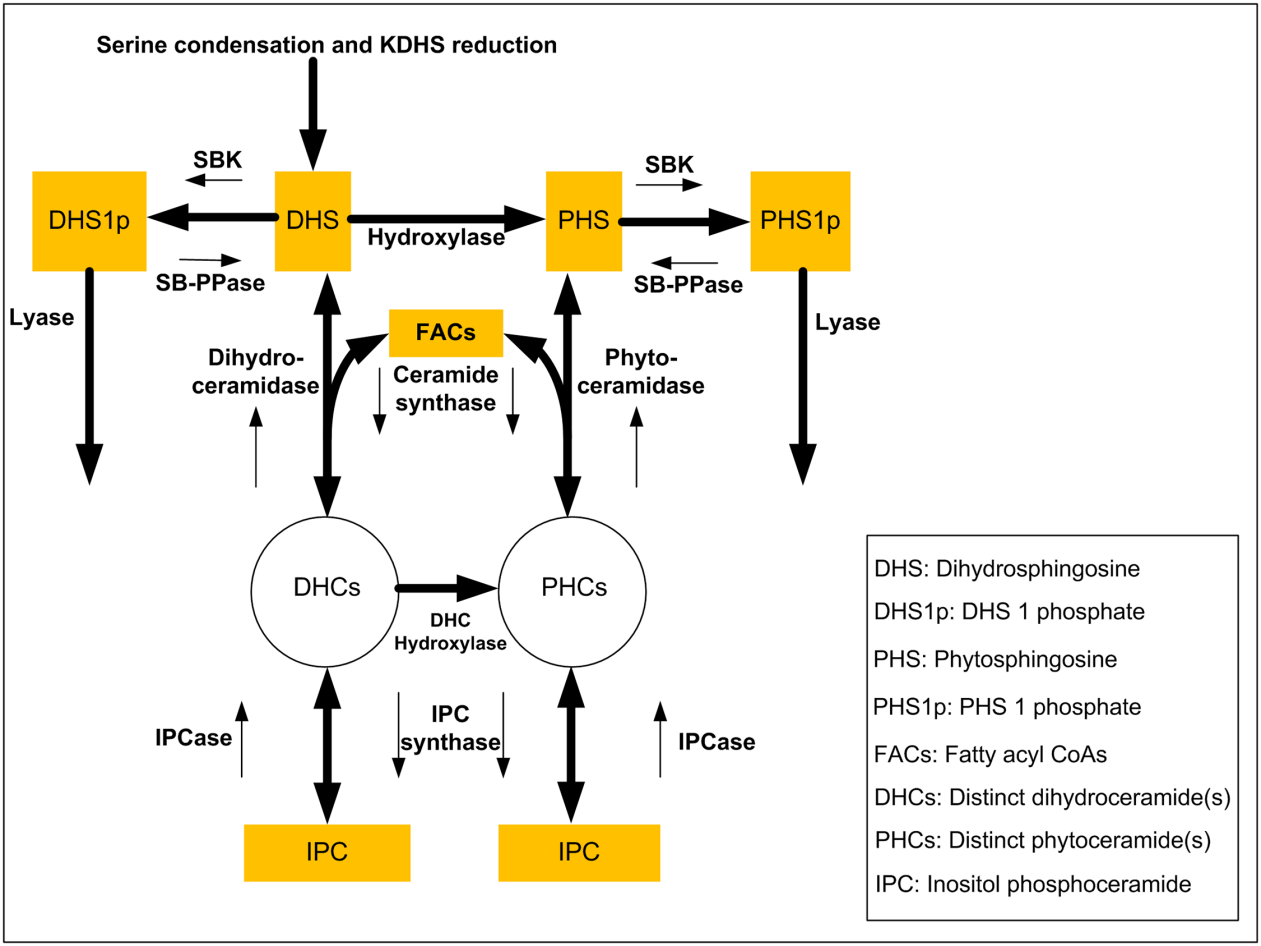
Pathway of ceramide biosynthesis in simplified representation. Yellow boxes indicate key sphingolipids and fatty acyl CoAs (FACs) included in our model. Two circles, DHCs and PHCs, represent distinct species of dihydroceramide and phytoceramide, respectively. The reversibility of reactions is indicated by thick, double-headed arrows, which are accompanied by thin unidirectional arrows and the names of the catalyzing enzymes. Details specifically associated with ceramides are given in Fig 2. The overall pathway shown here is a simplification of sphingolipid metabolism, which we have discussed and modeled elsewhere in great detail [13, 14].

**Fig 2:**
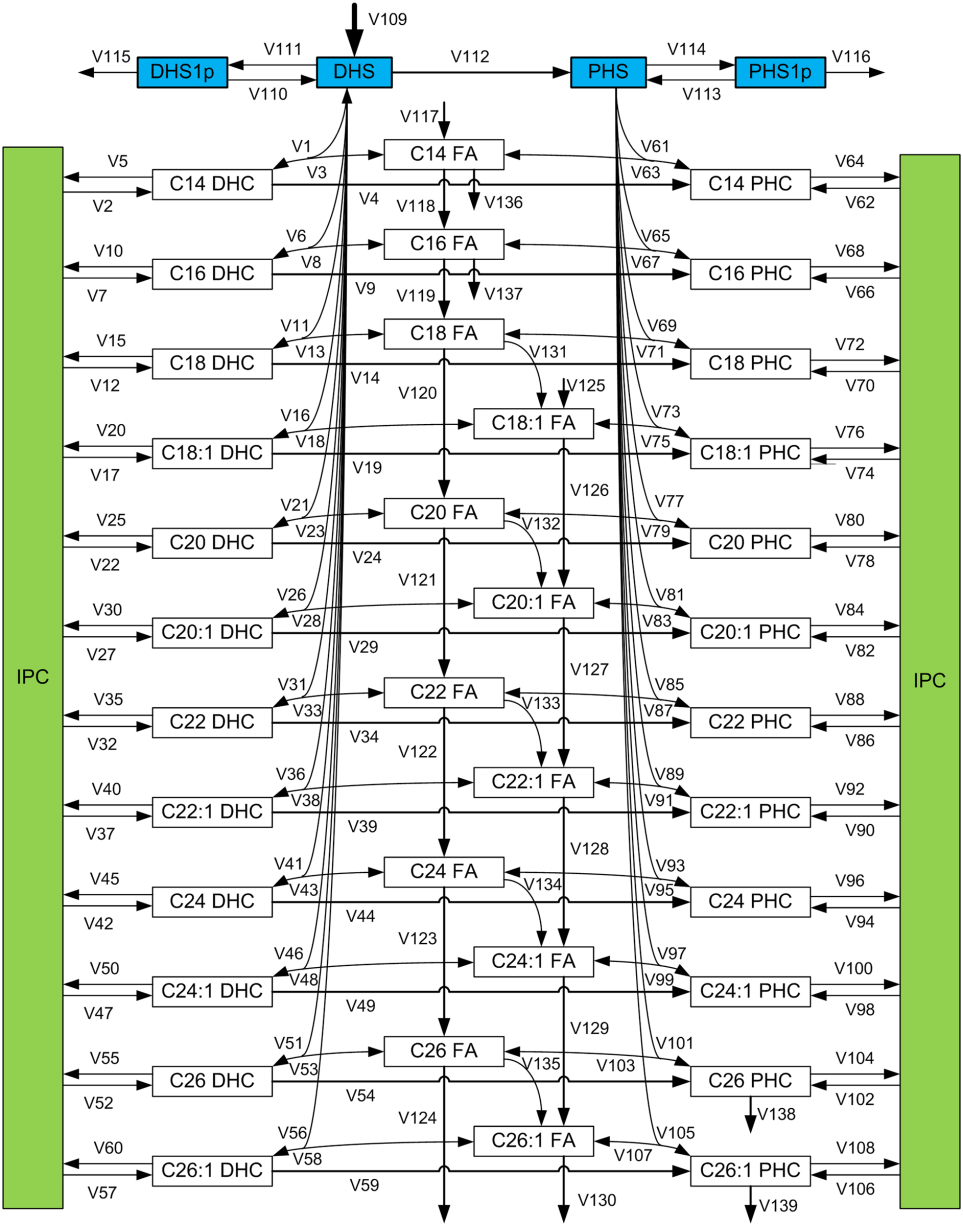
Scheme of ceramide metabolism that explicitly accounts for different fatty acyl (FA) chain lengths. The pathway consists of numerous parallel and cross reactions that lead to the production of distinct saturated and unsaturated dihydro- (DHC) and phyto- (PHC) ceramide variants with different FA chain lengths. The circles in Fig 1, containing DHCs and PHCs, are here expanded to represent 12 distinct DHC species on the left and 12 PHC species on the right, respectively. The green boxes represent IPC, and blue boxes represent corresponding sphingosine species. Fatty acyl CoA elongation is exhibited in the center of the pathway. Further details of ceramide metabolism are presented in the section *Ceramide Metabolism*.

While it has thus been suggested that yeast C18:1 PHC mediates the hydroxyurea stress response via a multi-step signaling cascade [9], it is not clear which mechanisms alter the level of this metabolite in the first place. Indeed, these mechanisms are not easy to decipher with intuition alone, because the biosynthesis of C18:1 PHC is embedded in the complicated pathway system of sphingolipid biosynthesis (Fig 1). This system, to which we will refer in the following sections as *ceramide metabolism*, actually consists of parallel pathways that generate and utilize saturated and unsaturated ceramides with different fatty acyl chain lengths. When formulated in sufficient detail to account for all different ceramide variants, this pathway is so complicated that targeted alterations of specific ceramide molecules can hardly be predicted without a targeted computational analysis (Fig 2). Nevertheless, it is important to shed light on the details of the involved metabolic processes because very specific ceramide variants appear to be the first responders to stresses, including hydroxyurea exposure and heat.

In the following, we will describe a computational approach that is based on a series of comprehensive sphingolipid models that our team has been developing over the past decade [11–15]. In contrast to all previous studies, many of which addressed heat stress responses, we focus here on cellular responses to hydroxyurea stress. The proposed approach consists of initial data processing, which addresses the sparseness and variance of the data, a piecewise optimization approach to estimate time coarse enzyme activities, and an analysis of the distribution of fluxes, which leads to an elucidation of the systemic mechanisms with which the ceramide pathway responds to hydroxyurea.

### Ceramide Metabolism

Ceramide is a crucially important species within the group of sphingolipids. It consists of a long chain base (LCB) backbone and a fatty acyl group. Long chain bases usually contain 18 carbons, but may also have 20. The most prevalent LCBs in mammalian cells are sphingosine, dihydrosphingosine (DHS) and phytosphingosine (PHS), whereas yeast only produces DHS and PHS. Most ceramide variants possess fatty acyl groups ranging from C12 to C26. Since we only consider C18 LCBs in this research, the distinct ceramide species can be named in accordance with their fatty acyl groups. For instance, C16 dihydroceramide (C16 DHC) denotes the ceramide N-palmitoyldihydrosphingosine or Cer(d18:0/16:0).

In yeast, ceramides can be generated through *de novo* biosynthesis and through the reutilization of complex sphingolipids (Fig 1). Sphingolipid biosynthesis begins with the condensation of serine and palmitoyl CoA, which is catalyzed by the enzyme serine palmitoyltransferase (SPT). This first step produces 3-keto-dihydrosphingosine (3KDHS), which is quickly reduced to DHS by the enzyme KDHS reductase. DHS is the key LCB and serves as a source for the other LCBs, PHS, as well as for DHC. An irreversible conversion of DHS to PHS is catalyzed by hydroxylase. Phosphate forms of DHS and PHS, DHS-p and PHS-p, can be reversibly converted by sphingoid base kinase (DHS to DHS-p and PHS to PHS-p) and sphingoid base phosphatase (DHS-p to DHS and PHS-p to PHS), respectively. DHS can also be converted to different variants of DHCs depending on the type of fatty acyl CoA the enzyme ceramide synthase uses. The reverse reaction from DHC to DHS is catalyzed by dihydroceramidase. PHS can be converted to PHC followed by a mechanism similar to that between DHS and DHC, but the reverse reaction is catalyzed by the specific enzyme phytoceramidase. The final reaction of the pathway system is DHC hydroxylase, which catalyzes the reaction from DHC and PHC.

The reutilization of complex sphingolipids constitutes an alternative path toward ceramide species. Complex sphingolipids include inositol phosphoceramide (IPC), mannose inositol phosphorylceramide (MIPC) and mannose di-inositol phosphorylceramide (MIP_2_C); all of these can serve as sources of ceramide species. IPC can be converted reversibly into MIPC, and MIPC can be converted reversibly into MIP_2_C. The reutilization of complex sphingolipids is catalyzed by the enzyme isc1 (IPCase), which is the yeast homologue to the mammalian neutral sphingomyelinases. The reverse reactions from DHC/PHC to IPC are catalyzed by the enzyme IPC synthase.

In summary, the ceramide levels are controlled by five enzymes, namely ceramide synthase, ceramidase (dihydroceramidase and phyotoceramidase), IPC synthase, IPCase (Isc1), and DHC hydroxylase. These reactions, combined with the array of different ceramide variants, form a complicated metabolic pathway system (Fig 2 and Methods section) that renders an intuitive understanding of the dynamic responses of hydroxyurea very challenging. To shed light on this system, we have developed a computational strategy that is based on an ordinary differential equation (ODE) model and uses experimental data as input (for details see Methods). This approach will assist us in the dissection of the regulatory strategies with which ceramide metabolism responds to hydroxyurea exposure.

## Results

The concentrations of ceramides depend on the fluxes that enter or leave each pool and add or utilize mass. The magnitudes of these fluxes are mainly determined by two factors: the level of substrate and the activity of the catalyzing enzyme. Within the framework of mass action kinetics, as well as other frameworks, such as the Michaelis-Menten rate law, the latter is often subsumed into the rate constant of the reaction. Using the techniques described in the *Methods* section, we have inferred these rate constants for all enzymatic steps involved. Summaries of these inferences are provided below. They are sorted by the five key enzymes of ceramide metabolism, namely, ceramide synthase, dihydroceramidase and phytoceramidase, IPC synthase, IPCase (Isc1), and DHC hydroxylase. Furthermore, because substrate concentrations can be inferred from interpolations of measured data, we are able to compute flux magnitudes. Thus, in the second half of this *Results* section, we describe insights from a detailed mass flow analysis that is based on these estimated flux magnitudes and offers novel insights into the mass flow patterns of different groups of ceramides.

### Enzyme Activities

Activity patterns of the key enzymes ceramide synthase, dihydroceramidase or phytoceramidase, IPC synthase, IPCase, DHC hydroxylase and other auxiliary enzymes were inferred and analyzed for the 20-hour experimental time period. Many factors can potentially influence these activities, including protein quantity, structure (folding/unfolding), metabolic regulation, and post-translational modifications. While it is impossible to characterize these secondary effects based on the currently available data, our computational approach reveals interesting regulatory strategies in several aspects, although it focuses on overall enzyme activities and rate constants.

The main results consist of two types. Time-dependent estimates are directly calculated from the optimization approach (see Methods). They are shown as gray stars, which represent individual simulation results, and blue lines, which depict simulation averages, as well as red linear regression lines. These regression lines immediately display the dynamic trends in enzyme activities during hydroxyurea exposure.

As an additional visualization, color-coded maps are computed from the dynamic time series. Specifically, the trends are simplified by two linear regressions, the first for the time period [0, 3], and the second for the time period [3, 20]; the cut at 3 hours reflects the measurement time point in the data. The trend for the first time window is shown in the left box at the top of each display associated with a reaction, while the trend for the second time window is shown in the right box on top. The wider box below these two displays the trend over the entire time period [0, 20]. These simplified representations immediately visualize the similarities and differences of dynamic enzyme activities within pools of reactions that involve the same specific enzyme.

#### 1. Ceramide synthase

**Fig 3:**
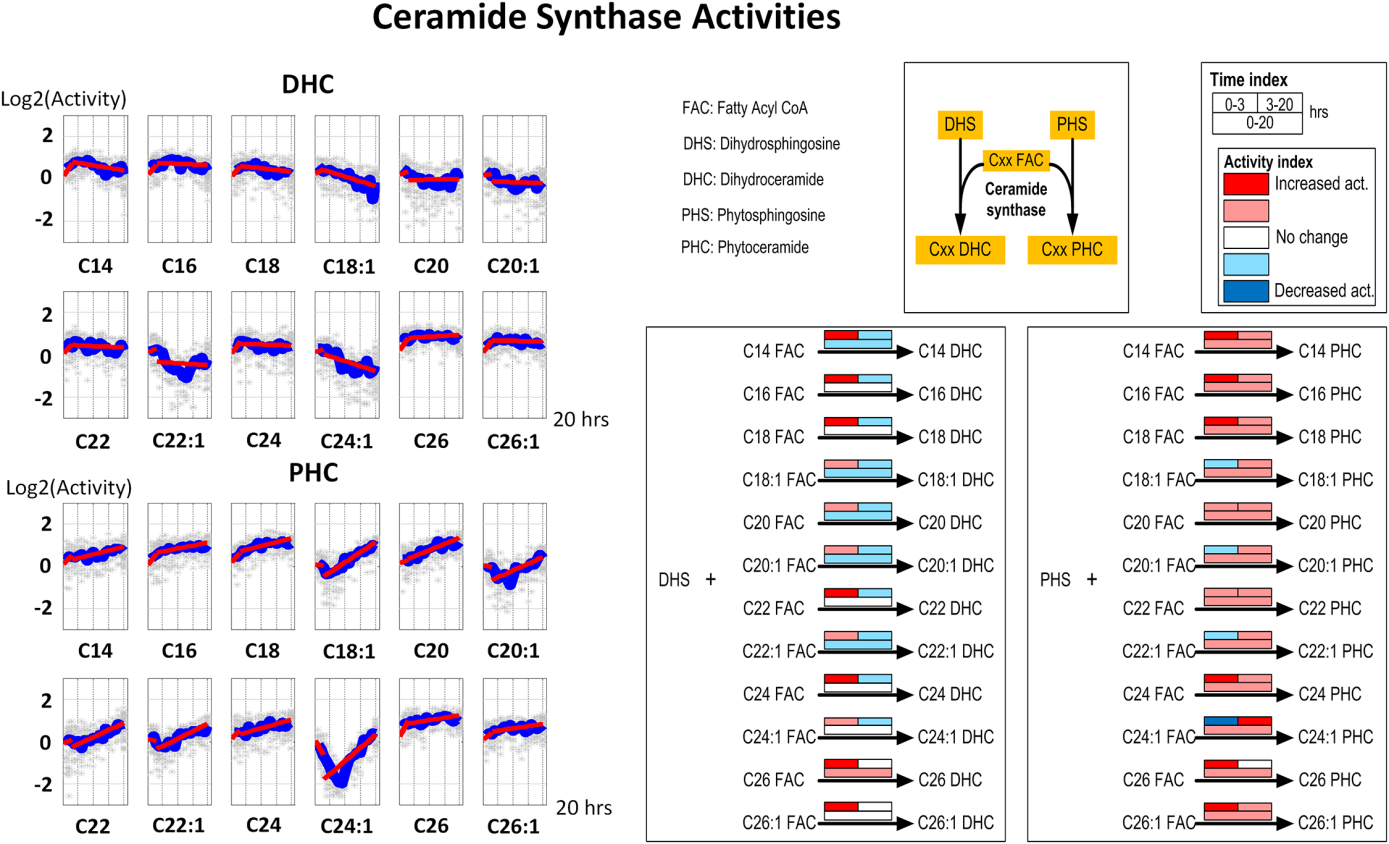
Left panel: Time series estimates of ceramide synthase activities. Each plot represents the ceramide synthase activities for a specific ceramide species. Gray stars represent individual simulation results and blue lines are averaged simulated activities. Each subplot contains two segments of red lines indicating linear regression over the intervals of 0–3 hours and 3–20 hours. Right panel: Color-coded map. Each array of boxes associated with a reaction step indicates average slopes of the dynamic trends shown in the left panel for three time intervals: 0–3 (top left box), 3–20 (top right box) and 0–20 hours (wide bottom box).

Ceramide synthase shows different patterns of activities when using DHS or PHS as substrate. In detail, the activities of ceramide synthase using DHS as a substrate exhibit a gradually decreasing pattern for fatty acyl chain lengths up to C24 DHC, while the corresponding reactions using PHS as substrate show increasing trends (except for C24:1 PHC). This finding can be further analyzed by means of the color-code maps in Fig 3. Interestingly, the reaction rates of these groups show opposite directions over the entire 20-hour experiment. Specifically, all color boxes at the bottom of each reaction for DHC are light blue, indicating a slight overall decrease, while they are pink for PHC, which indicates a slight overall increase. In addition to these overall trends, one finds differences in activities during the [0, 3] and [3, 20] hour windows.

The different patterns of ceramide synthase activities may be interpreted in two ways. First, the biosynthesis of sphingolipids is proceeding at a moderate pace, since most of the activities of ceramide synthase are within a 2-fold range. At the same time, the slight but important trends of gradually decreasing or increasing ceramide synthase activities in DHS versus PHS may be due to competition between DHS and PHS toward ceramide synthase.

#### 2. Ceramidase

**Fig 4:**
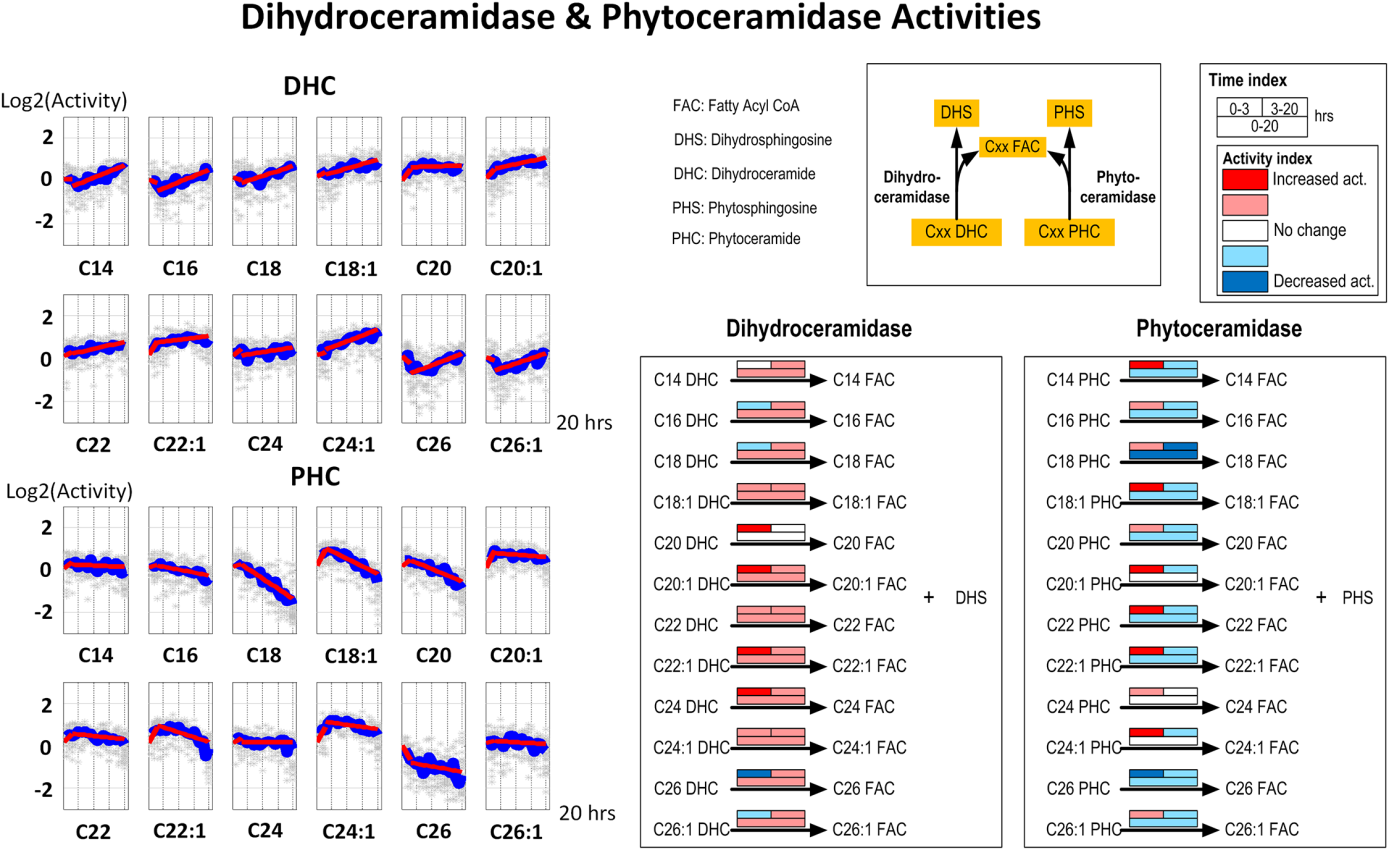
Left panel: Time series estimates of dihydroceramidase and phytoceramidase activities. Each plot represents the ceramidase activities for a specific ceramide species. Gray stars represent individual simulation and blue lines are averaged simulated activities. Each subplot contains two segments of red lines indicating linear regression over the intervals of 0–3 hours and 3–20 hours. Right panel: Color-coded map. Each array of boxes associated with a reaction step indicates average slopes of the dynamic trends shown in the left panel for three time intervals: 0–3 (top left box), 3–20 (top right box) and 0–20 hours (wide bottom box).

Ceramidases (dihydroceramidase and phytoceramidase) and IPC synthase catalyze the effluxes of ceramides; expressed differently, up-regulation of these enzymes decreases the ceramide concentrations. The cellular strategy of increasing dihydroceramidase activity to regulate the amount of DHC under heat stress has been documented. Our simulation results suggest a similar strategy under hydroxyurea stress (Fig 4). In a very consistent pattern, the activities of dihydroceramidase gradually increase while trends of phytoceramidase drop. These trends eventually result in a relatively low DHC level and a high PHC concentration.

#### 3. IPC synthase

**Fig 5:**
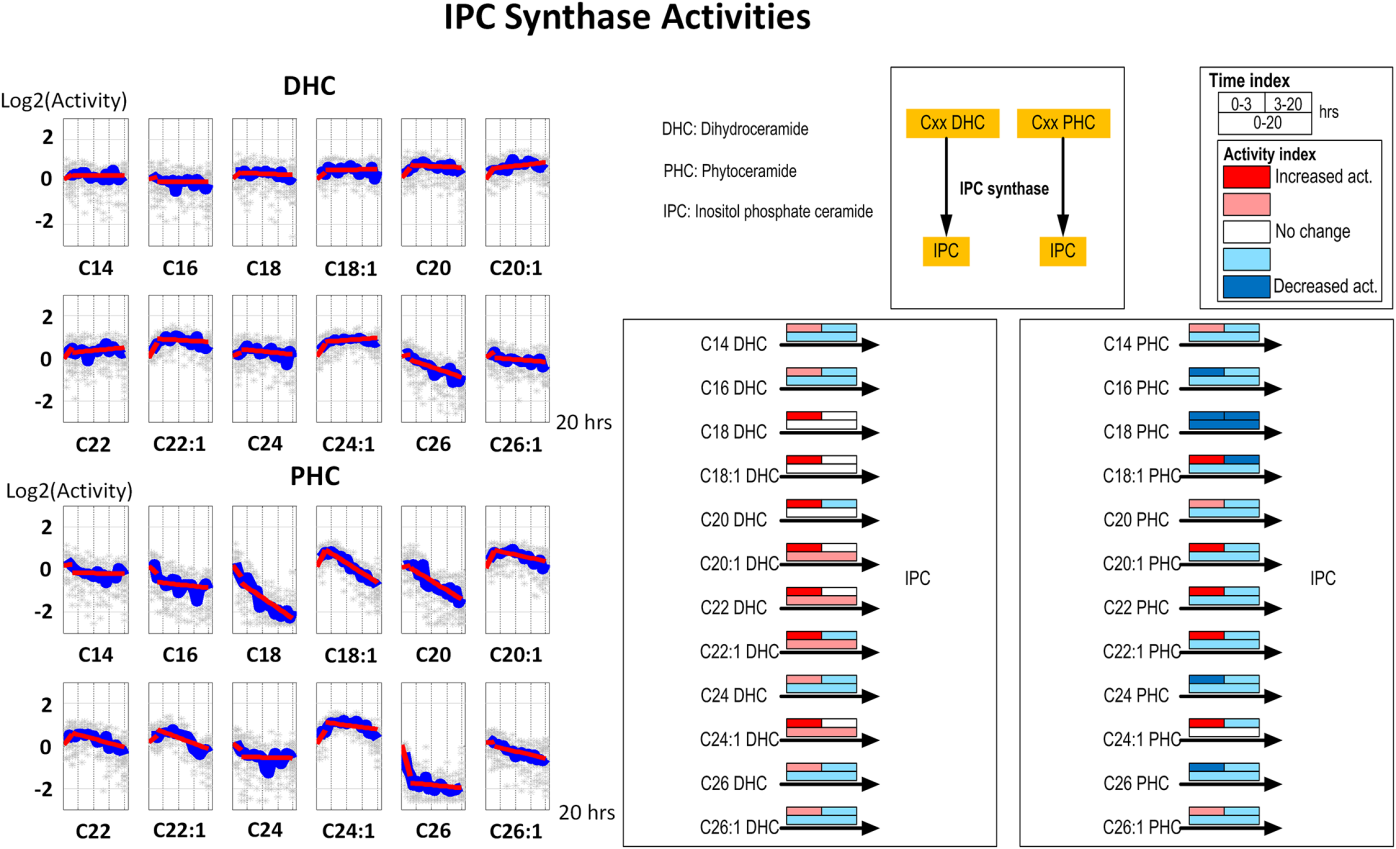
Left panel: Time series estimates of IPC synthase activities. Each plot represents the IPC synthase activities for a specific ceramide species. Gray stars represent individual simulation and blue lines are averaged simulated activities. Each subplot contains two segments of red lines indicating linear regression over the intervals of 0–3 hours and 3–20 hours. Right panel: Color-coded map. Each array of boxes associated with a reaction step indicates average slopes of the dynamic trends shown in the left panel for three time intervals: 0–3 (top left box), 3–20 (top right box) and 0–20 hours (wide bottom box).

The activities of IPC synthase, which uses DHC or PHC as substrate, show similar patterns as dihydroceramidase and phytoceramidase, respectively (Fig 5). This finding could suggest that IPC synthase might be a regulator for balancing DHC and PHC concentrations. However, the existence of only one IPC synthase in yeast limits this potential regulatory role. In fact, the color-code representation in the right panel of Fig 5 exhibits lower decreases in activities for almost all ceramide species compared with ceramidases. The distinct patterns of time-dependent activities might result from substrate competition toward IPC synthase or from compartmentalization.

Although the changes in activities of IPCase and the ceramidases look somewhat similar, the overall trends, as given in the color maps, are distinct. The strong contrast in ceramidase activity for DHC and PHC may suggest the existence of two enzyme variants (dihydroceramidase and phytoceramidase) while the similarity of IPC synthase activities suggests that there is only one IPC synthase enzyme in yeast.

#### 4. IPCase (Isc1)

**Fig 6:**
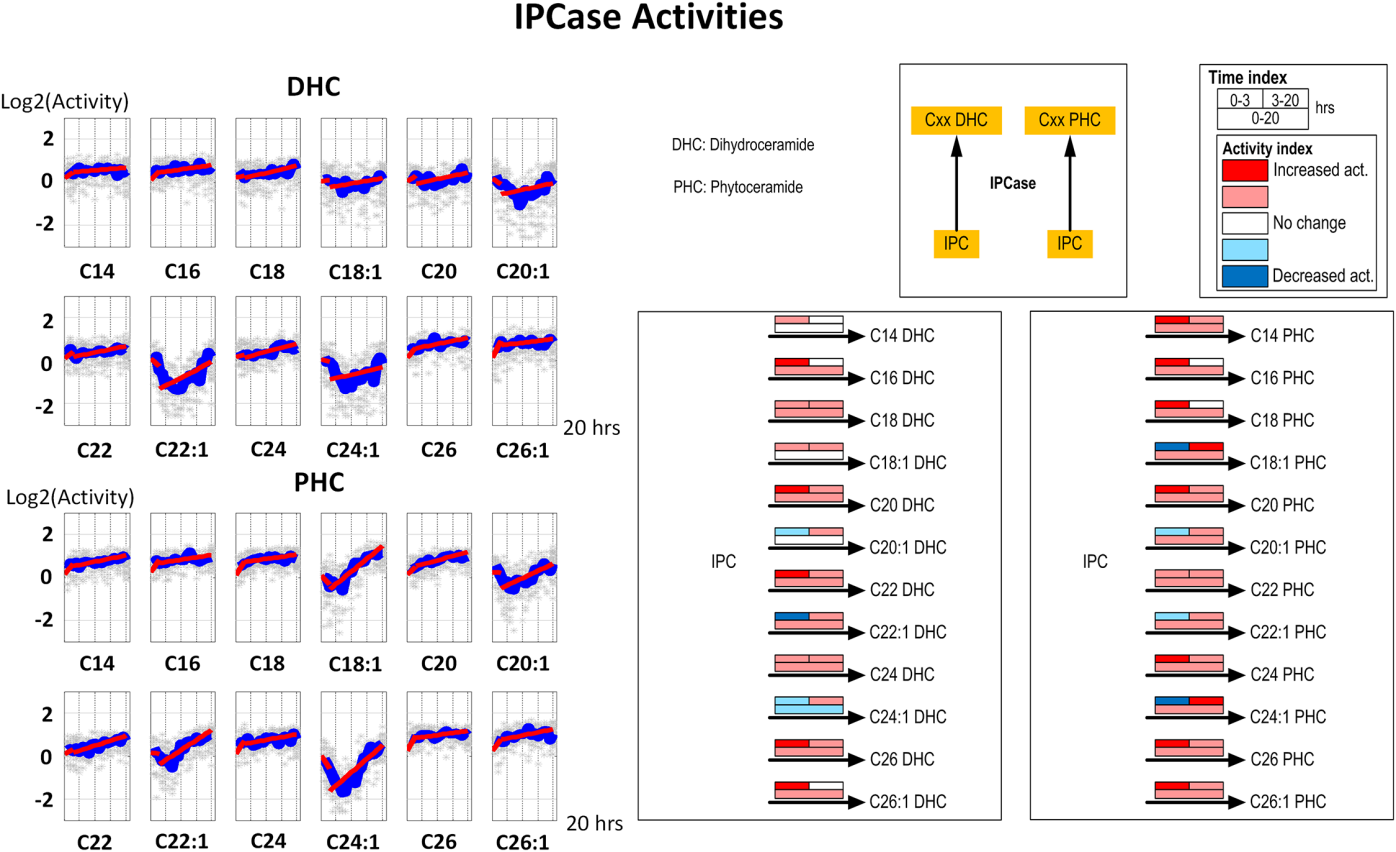
Left panel: Time series estimates of IPCase (Isc1) activities. Each plot represents the IPCase activities for a specific ceramide species. Gray stars represent individual simulation and blue lines are averaged simulated activities. Each subplot contains two segments of red lines indicating linear regression over the intervals of 0–3 hours and 3–20 hours. Right panel: Color-coded map. Each array of boxes associated with a reaction step indicates average slopes of the dynamic trends shown in the left panel for three time intervals: 0–3 (top left box), 3–20 (top right box) and 0–20 hours (wide bottom box).

Under hydroxyurea stress, up-regulation of IPCase, or Isc1, has been reported in the literature. In our simulation, IPCase activities match this finding over the 20-hour period in most of the reactions, although the initial activity decreases in several cases. These cases, C22:1 DHC, C24:1 DHC, C18:1 PHC and C24:1 PHC, may be explainable with substrate competition toward IPCase, since IPC forms different substrates from IPC, MIPC and MIP_2_C, and each of these contains distinct fatty acyl groups. However, the transient lower activities in the first 5 to 7 hours are compensated by later activation, which results in increased long term activities (Fig 6).

C18:1 PHC has been identified as a key signaling intermediate under hydroxyurea stress in yeast. Our simulation, instead of identifying a single key enzyme (such as IPCase) that would cause this increase, suggests a highly coordinated strategy that: (1) slightly increases ceramide synthase activity using PHS and C18:1 fatty acyl CoA as substrates (Fig 3); (2) slightly decreases phytoceramidase and IPC synthase activity using C18:1 PHC as substrate (Figs 4 and 5); and (3) subtly modulates IPCase activity (toward C18:1 PHC) to achieve the overall C18:1 PHC level (Fig 6). This postulated strategy suggests an energetically and metabolically effective way to control metabolites. A similar inference was made for changes in sphingolipid metabolism during the diauxic shift and during heat stress, where many enzyme activities were altered rather slightly, rather than a few enzymes that were altered much [11, 16].

#### 5. DHC Hydroxylase

**Fig 7:**
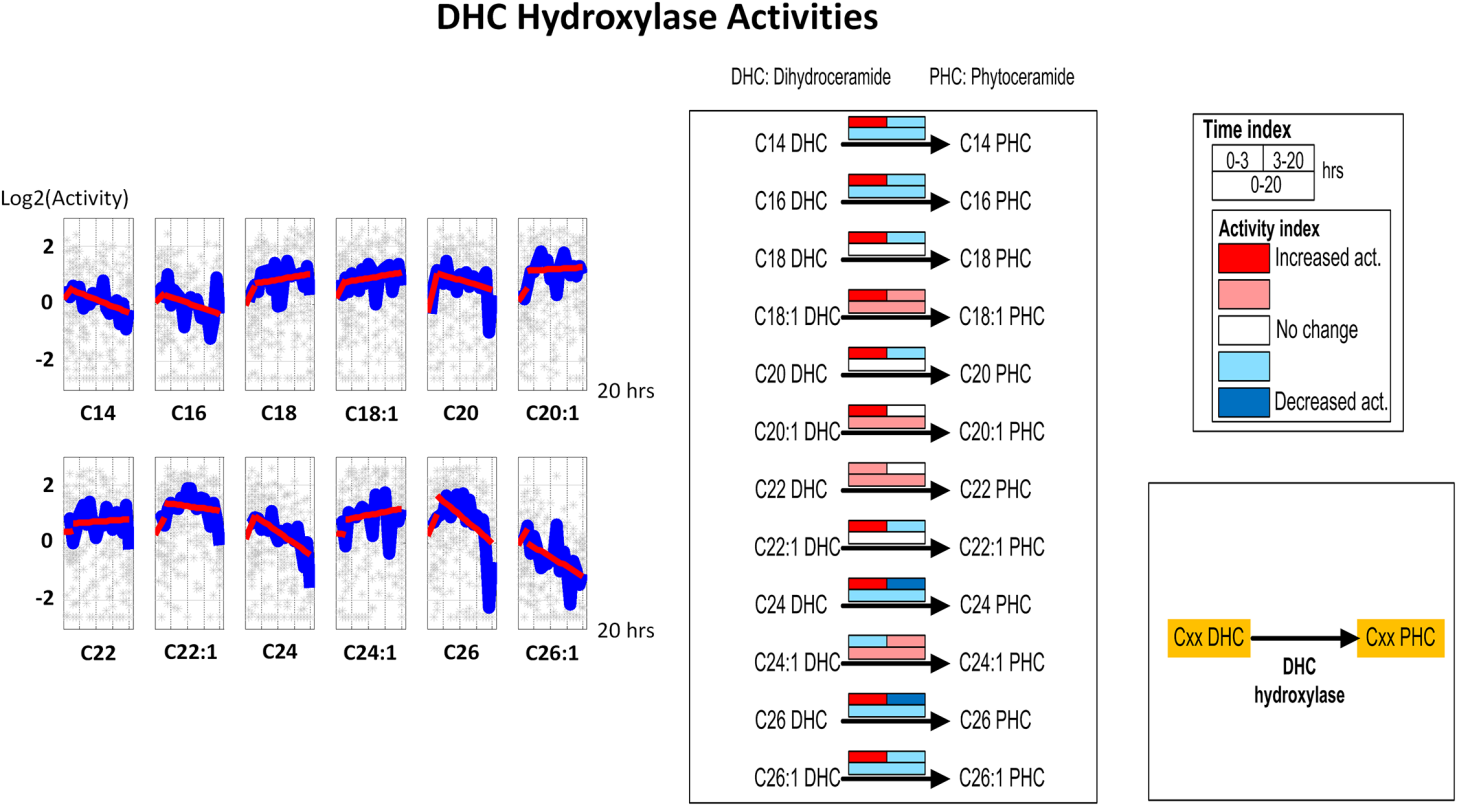
Left panel: Time series estimates of DHC hydroxylase activities. Gray stars represent individual simulation and blue lines are averaged simulated activities. Each subplot contains two segments of red lines indicating linear regression over the intervals of 0–3 hours and 3–20 hours. Right panel: Color-coded map. Each array of boxes associated with a reaction step indicates average slopes of the dynamic trends shown in the left panel for three time intervals: 0–3 (top left box), 3–20 (top right box) and 0–20 hours (wide bottom box).

Simulations of DHC hydroxylase reveal no real trends, especially when the entire 3–20 hour experiment is considered (Fig 7). The activities do change somewhat for different DHC variants, but these slight changes may be compensatory.

#### 6. Other enzymes

Enzymes associated with sphingolipid biosynthesis, fatty acid elongases and desaturase, are not as important as the enzymes listed above for regulating ceramide concentrations. However, these enzymes do affect the balances between auxiliary metabolites, such as DHS, PHS and fatty acyl CoAs, which are crucial for maintaining the appropriate ceramide concentrations. Therefore, it is beneficial to discuss the activities of these enzymes as well.

**Fig 8:**
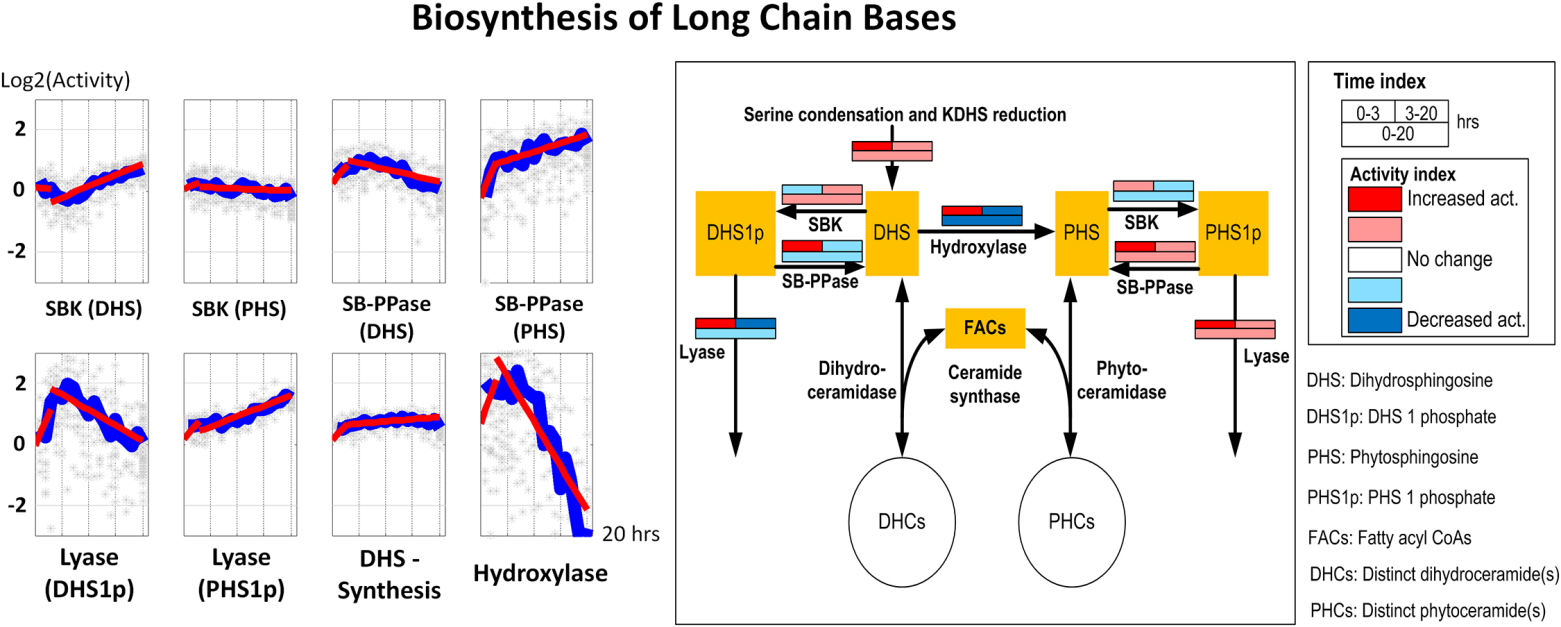
Left panel: Time series activities of enzymes catalyzing sphingolipid biosynthesis. Gray stars represent individual simulation and blue lines are averaged simulated activities. Each subplot contains two segments of red lines indicating linear regression over the intervals of 0–3 hours and 3–20 hours. Right panel: Color-coded map. Each array of boxes associated with a reaction step indicates average slopes of the dynamic trends shown in the left panel for three time intervals: 0–3 (top left box), 3–20 (top right box) and 0–20 hours (wide bottom box).

*Sphingolipid biosynthesis and utilization*. DHS biosynthesis (which here combines palmitate uptake, serine condensation and KDHS desaturation) remains constantly upregulated throughout the 20-hour experiment. This observation suggests a persistent uptake of materials from the medium. The remaining enzymes, SBK (sphingoid base kinase), SB-PPase (sphingoid base phosphatase), lyases (which remove DHS-p and PHS-p from the system) and hydroxylase (which converts DHS into PHS), along with ceramide synthase and ceramidases, cooperatively maintain DHS, PHS, DHS-p and PHS-p concentrations at appropriate levels (Fig 8).

**Fig 9:**
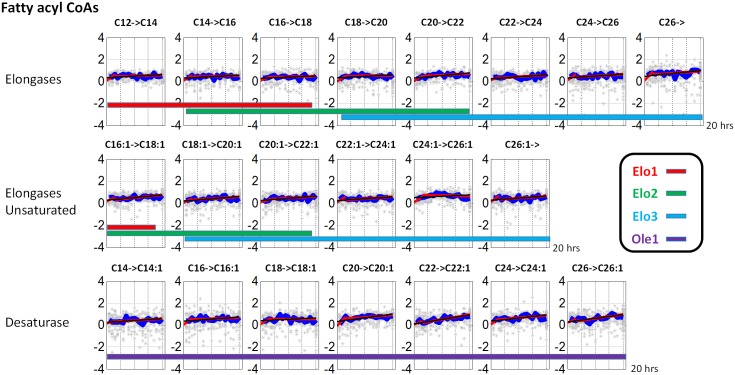
Activity estimates of elongases and desaturase over the 20-hour experimental time period.

*Elongases and desaturase*. Yeast expresses three elongases, Elo1, Elo2 and Elo3, which catalyze elongation reactions from C12 to C18, C14 to C22, and C18 to C26 fatty acid, respectively. Furthermore, desaturase (Ole1) catalyzes reactions from saturated to unsaturated fatty acyl CoAs. Estimates of the activities of these enzymes all show unremarkable patterns throughout the 20-hour experiment, even though the desaturase active rises slightly with time (Fig 9). The reason for their constancy might be that fatty acid elongation is an important cellular event, and it appears that the demand for fatty acids of various chain lengths does not change much over time.

#### 7. Mass Flow Analysis

The flux into or out of a metabolite pool is determined by the concentrations of substrates and the rate constant, which here includes the enzyme activity. Thus, in order to assess changes in flux magnitudes, it is not sufficient to study changes in rate constants alone. In consideration of this fact we conducted a mass flow analysis that estimates materials entering and leaving pools within the system from estimated rate constants (details in Methods) and measured or interpolated substrate concentrations.

Once all dynamically changing fluxes are computed for the experimental time interval, it is possible to assess the mass flow throughout the entire system during the 20 hours of hydroxyurea stress (see Methods for details). This mass flow, especially around ceramides, including DHCs, PHCs and fatty acyl CoAs, suggests how materials are synthesized, degraded or recycled during the 0–3 hour and 3–20 hour time periods, and throughout the entire 20 hours of hydroxyurea stress.

**Fig 10:**
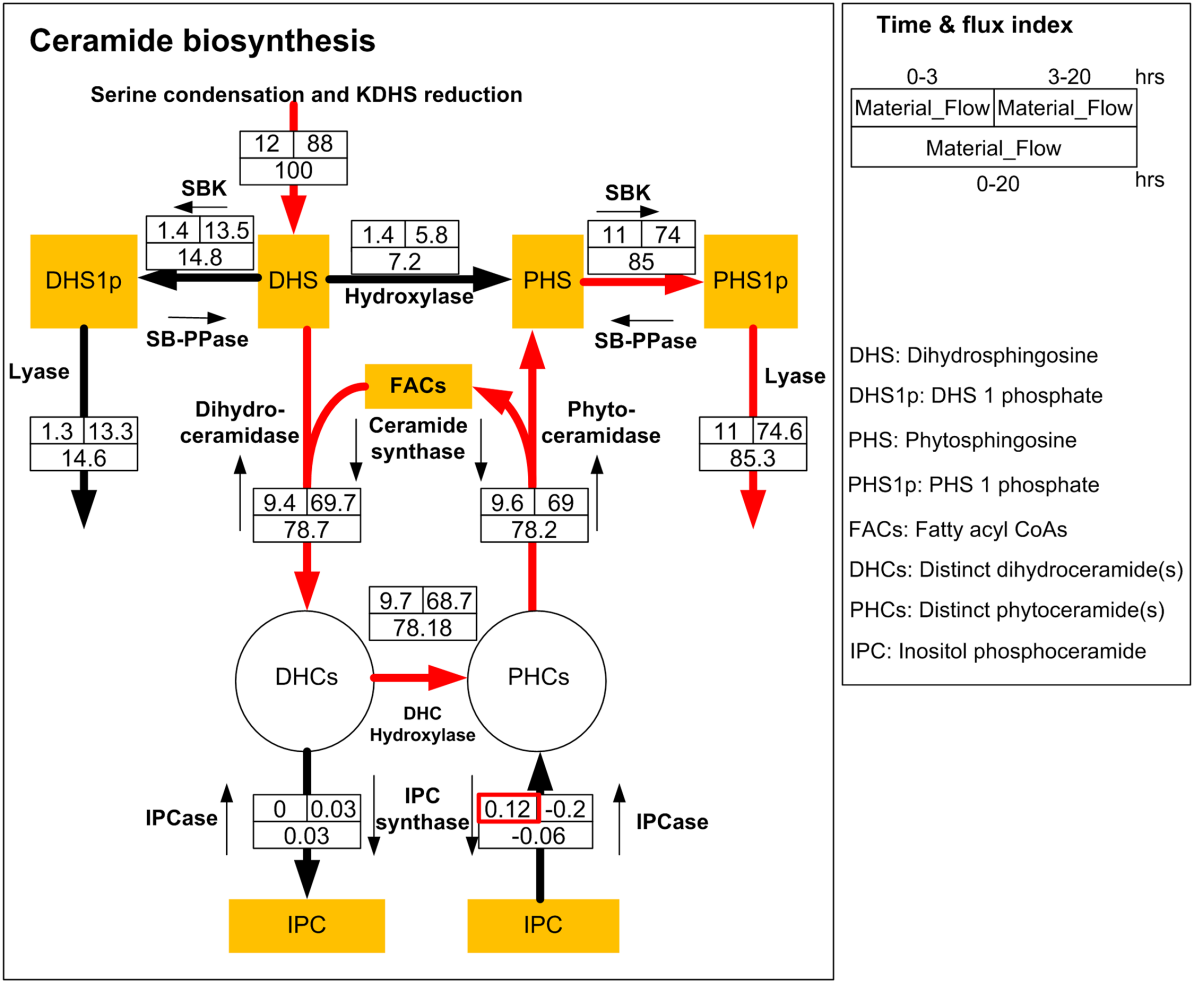
Mass flow through the pathway of ceramide biosynthesis (with different DHCs, PHCs and FAs merged into single pools). Red arrows indicate the main flow of material throughout 20 hours of hydroxyurea exposure.

The flux distribution in Fig 10 indicates that sphingolipid biosynthesis is always active. DHCs and PHCs are maintained through two main fluxes. One is the net flux of the balance between ceramide synthase on the one hand and dihydroceramidase or phytoceramidase on the other. This net flux is always high, particularly during the [3, 20] hour time window. Interestingly, the net fluxes point from DHS to DHC, but from PHC to PHS. The other flux is controlled by DHC hydroxylase. It is similarly high and carries much more mass than the small positive net flow from IPC to PHCs in the first three hours, which is actually reversed during the second phase of the experiment. Thus, it is interesting to note that material flows in a consistent manner through the system from DHS to DHC to PHC to PHS to PHS-p, from where lyase removes material from ceramide metabolism. Expressed differently, there is no drastic change of this flux pattern throughout the 20 hours of stress, and there is no change in the direction of mass flow.

This result raises the question of how the cells manage these fluxes in such a manner that all ceramide species achieve their target levels. Specifically, one must ask: if there is no evidence indicating any flux reversal, how is it possible that distinct saturated or unsaturated fatty acyl CoAs can be channeled toward the accumulation of a key species like C18:1 PHC?

**Fig 11:**
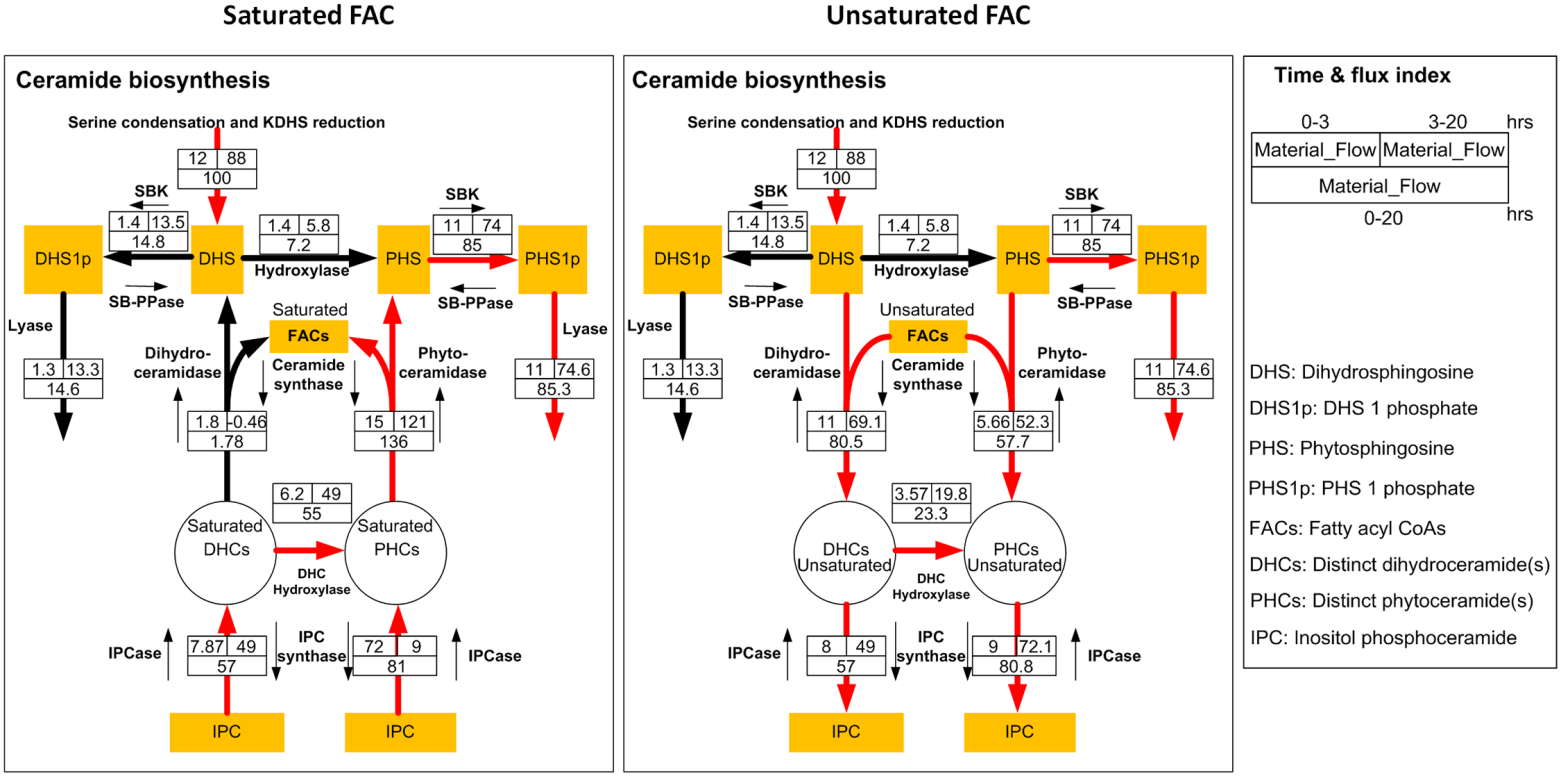
Mass flow analysis of the ceramide pathway separated for saturated and unsaturated fatty acyl groups. Red arrows indicate the main flow of mass in each system during the 20-hour experimental time period.

To assess this question, we analyzed the mass flow for saturated and unsaturated fatty acyl CoAs separately. Intriguingly, the flow patterns now are distinctly different. Fig 11 shows the mass flow of the pathway for saturated and unsaturated fatty acyl CoAs in the left and right panels, respectively.

As a first result, one notices that ceramides with saturated fatty acyl group are mainly provided through the degradation of complex sphingolipids, whereas ceramides with unsaturated fatty acyl group are obtained from biosynthesis via DHCs.

This result in turn suggests that increased Isc1 (IPCase) activity during 20 hours of hydroxyurea treatment affects saturated and unsaturated ceramides differently, which might explain findings regarding Isc1 in the literature. Such a differential effect does not seem unreasonable as saturated and unsaturated ceramides have distinct molecular shapes. According to our analysis, hydroxyurea increases the net flow from IPC to those ceramides containing saturated fatty acyl CoAs, and the PHC pool is being recycled via PHS, PHS-p, and the lyase reaction. At the same time, Isc1 apparently prevents unsaturated ceramides from being channeled toward IPC. As a consequence, knocking down Isc1 indirectly permits the conversion of PHCs to IPC. PHCs, including C18:1 PHC, eventually become depleted, and cells are no longer tolerant to hydroxyurea and die.

Fig 10 can also be interpreted from the perspective of fatty acyl CoAs (FACs). The left panel demonstrates a strong net flux into saturated FACs, while the right panel suggests a strong net efflux out of unsaturated FACs. A possible interpretation is the following: under hydroxyurea exposure, saturated FACs are used for the conversion of saturated DHCs into PHCs and can be converted into unsaturated FACs through the desaturase reaction, which exhibits increased activity. Unsaturated FACs can then be used to provide corresponding DHCs and PHCs.

## Discussion

Sphingolipids were originally identified in brain extracts, and it was later determined that they serve as integral components of membranes. It also became clear quickly that sphingolipids play enormously important roles in mediating a number of crucial functions in eukaryotic cells. In particular, it was recognized that sphingolipids can quasi serve as sensors for external perturbations, such as heat stress, and that they can trigger specific changes in gene expression, which secondarily lead to the mounting of well-coordinated responses.

Analyzing sphingolipid-mediated signaling is not trivial, as the biosynthesis, interconversions, and interactions of sphingolipids and ceramides with membranes constitute a very complex, highly regulated system, which makes intuitive reasoning problematic. To overcome this issue, we have been developing dynamic models over the past decade that capture the dynamics of sphingolipids with a rather high degree of resolution [13, 14, 17].

In previous studies [15], we analyzed stress responses and identified not only the roles of particular ceramide species, but also the roles of the same types of ceramide with different fatty acyl chain lengths. To achieve the appropriate profiles of ceramide species with different fatty acyl chain lengths, we found that yeast enrolls essentially all enzymes associated with sphingolipid metabolism, with the consequence that numerous relatively small alterations are sufficient to achieve the target profiles. More generally, our results left no doubt that the ceramide response to heat stress is not a haphazard panic reaction, but a highly cooperative, well-coordinated cellular adaptation.

In the present work, we studied the responses to exposure to hydroxyurea, a substance that is increasingly finding new treatment uses, because it reduces or even halts DNA synthesis. Interestingly, not much is known about the specific mechanisms of hydroxyurea exposure, although it has become clear that some of its effects are mediated by ceramides of different fatty acyl chain lengths. We therefore set out to infer these mechanisms through a combination of data analysis, mathematical ensemble modeling, and customized optimization.

The algorithmic strategy (as described in the Methods section) was designed to be sufficiently robust to tolerate inaccuracies within a highly dynamic dataset. This robustness was achieved by a novel combination of a custom-tailored resampling strategy with a piecewise optimization approach. The result does not consist of a single model but of an entire ensemble of models that match all observations with acceptable accuracy. Nonetheless, the conclusions drawn from our analysis should be considered with caution, as they are entirely computational and have not been independently validated by new experiments.

Our results suggest that the differential use of saturated and unsaturated FACs affords the cell with a regulatory mechanism beyond typical feedback and feedforward activation and inhibition or the use of specialized enzymes. These simulation results should be independently validated, but if they are correct, they provide novel insights into the regulatory mechanisms that govern—or at least contribute to—the control of distinct ceramide species. This regulation task is complicated by the fact that the system contains many metabolites but only a few enzymes, so that alterations in enzyme activities are quite limited when subtle changes of specific metabolites are needed. For example, increasing ceramide synthase activity elevates the rates of 12 reactions toward synthesizing distinct ceramide species in our model and does not by itself allow a change in just one or a few reaction products. Similar mechanisms in phospholipid metabolism have been identified as Lands’ cycle (remodeling pathway) where distinct phospholipids can be converted to lysophospholipids catalyzed by phospholipase A2, and the reverse reactions can be catalyzed by distinct acyltransferases with different acyl group selectivity [18, 19]. Ceramide species with distinct fatty acyl groups may be controlled by similar mechanism. Our simulations suggest that cells manage to achieve hydroxyurea tolerance through the well-coordinated, differential usage of saturated and unsaturated fatty acyl groups. In other words, the substrate affinity of enzymes toward saturated or unsaturated fatty acyl groups seems to be distinct and appears to constitute an additional mode of regulation, which ultimately permits the fine-tuning of a desired ceramide profile.

While the analysis identifies Isc1 as an enzyme that differentiates substrates by saturation state, it is most likely not the only such enzyme. In fact, it seems that ceramide synthase, dihydro- and phyto-ceramidase, IPC synthase, DHC hydroxylase all help to establish and maintain the appropriate ceramide profile.

## Methods

### Summary of the Experimental Methods

The sphingolipid measurements used to parameterize the model were obtained by exposing a culture of the wild-type yeast *Saccharomyces cerevisiae* strain JK9-3d (*MAT****a*** *trp1 leu2-3 his4 ura3 ade2 rme1*) cells to 10 mg/ml of hydroxyurea [9]. Specifically, a culture, grown overnight, was re-suspended to A_600_ of 0.15 in 50 ml of medium (YPDA) and allowed to reach stationary phase at an A_600_ of 0.7. At this point the cultures were treated with either hydroxyurea or vehicle (culture media without hydroxyurea). After 3 and 20 hours of treatment, samples of the culture were taken, the cells were centrifuged and the pellets used for lipid extraction with solvent containing: iso-propanol (50%), diethyl ether (10%), pyridine (2%), ammonia (25%) and water (15%). Lipids were analyzed in the Lipidomic Core at the Medical University of South Carolina by HPLC-MS/MS as previously described [9, 10]. Fold changes of the different lipid species were calculated by dividing amounts measured in hydroxyurea treated culture by the values obtained in the control cultures.

### Time Courses of Ceramide Concentrations

**Fig 12:**
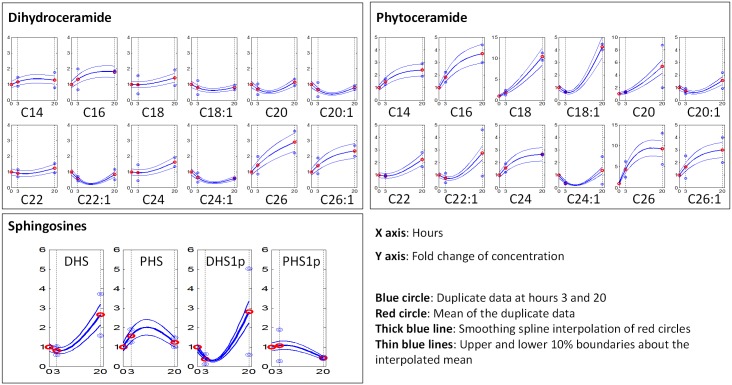
Interpolation of raw duplicate data. The duplicate time course data, means of data, interpolation of mean data and ±10% variability bands are presented in each plot as blue circles, red circles, a thick blue line, and thin blue lines, respectively.

Ceramide concentrations were measured in duplicate as fold changes, relative to the steady state at time 0, after 3 and 20 hours of exposure to hydroxyurea [9]. The measurements are presented in S1 File. Mean values of the duplicate measurements were interpolated with a smoothing spline, and this interpolation constituted the baseline levels for the dynamic trends of ceramides. The interpolated mean trends do not reflect any variability in the duplicate raw data. Therefore, we allowed for variations within 10% upper and lower boundaries relative to the interpolated baseline trends. The results are represented in Fig 12. The semi-artificial data we used to fit with our models were randomly sampled from these 20% ranges. Given the scarcity of the data, this strategy was chosen as a means of taken into account the genuine variance in the data. As a consequence, our model is not intended to fit any particular data points but to return simulation results within the 20% target range at each time point.

### Mathematical Model

The proposed ceramide pathway model contains 137 reactions (fluxes) and 42 dependent variables. It corresponds directly to the schema in Fig 2; numerical and technical details are presented in the S1 and S2 Files. The pathway system accounts for many parallel reactions, for which kinetic parameters are not known. Furthermore, absolute ceramide concentrations are not available. To minimize the consequences of this paucity of information, we chose a mass action model to infer coarse trends in enzyme activities. It seems reasonable to assume that the cells operate relatively close to steady states at the selected time points. At these states, mass action, power-law, and Michaelis-Menten models yield results that differ much less than the data we have.

As an example for the design of this mass action model, consider flux *V*_1_ in Fig 2, which represents the reaction

DHS + C16 Fatty acyl CoA →C16 DHC.

The mass action model for this reaction is defined as *V*_1_ = *γ*_1_ [DHS] [C16FAC], where [DHS] and [C16 FAC] represent the concentrations (or fold changes) of DHS and C16 Fatty acyl CoA (relative to their steady states), respectively, and *γ*_1_ denotes the rate constant. Each rate constant is considered here to include the corresponding enzyme activity. This merging of factors is similar to the combination of *k*_*cat*_ and the total enzyme concentration in the Michaelis-Menten formalism, which results in *V*_*max*_. In the mass action model resulting from these settings, the steady-state flux distribution (and the rate constants) can be estimated and tested quite rapidly.

### Piecewise Optimization Approach & Linear Interpolation

Steady-state fluxes and steady-state enzyme activities were calculated with a constrained optimization approach. Using a resampling scheme for the semi-artificial “data” from the range surrounding the first data point, corresponding averaged enzyme activities at the steady state were computed. They were tested by entering them into the mass action model and ensuring that they satisfied the steady state. If so, they were used as baseline activities.

**Fig 13:**
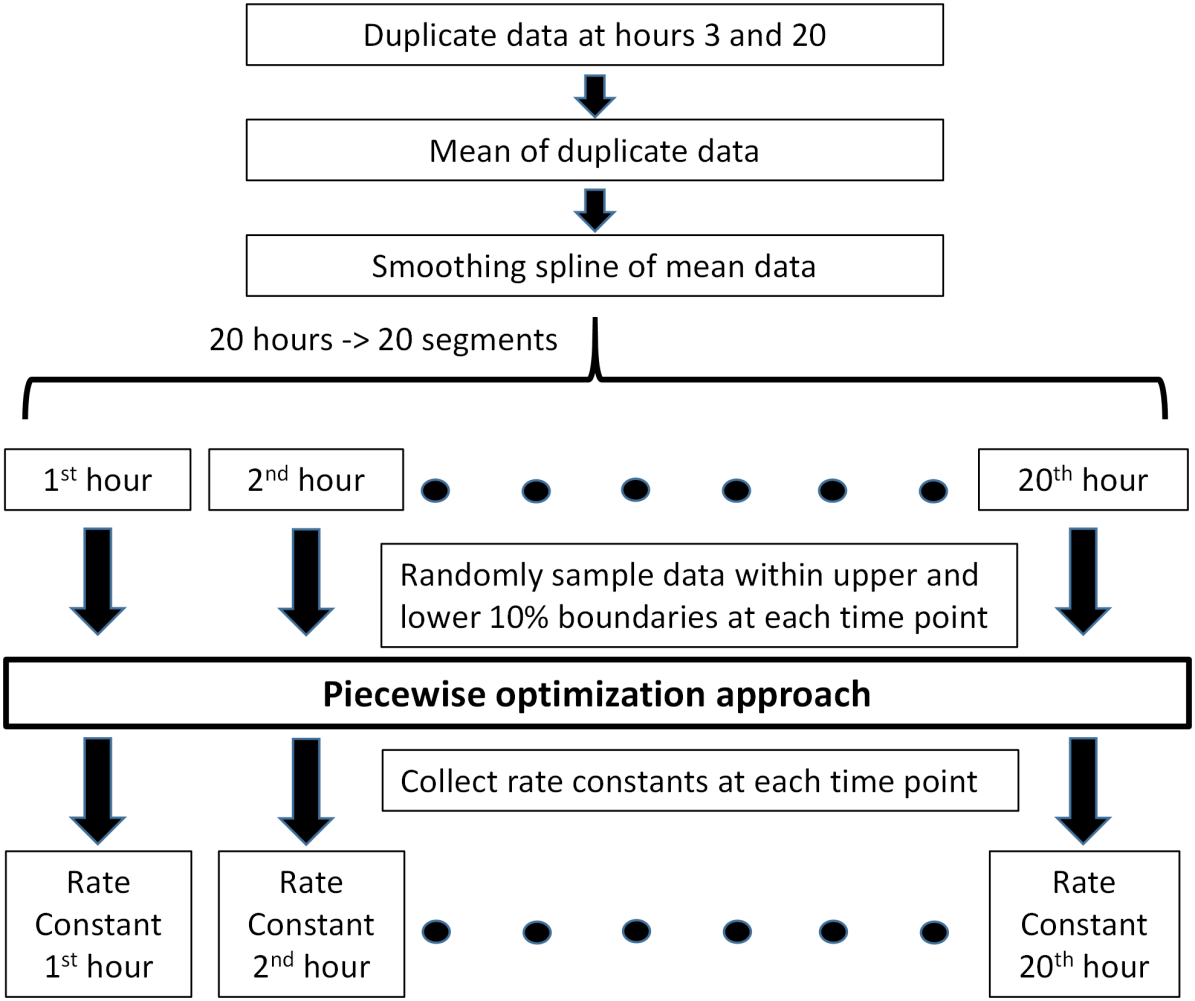
Flowchart of the piecewise optimization approach.

A piecewise optimization strategy, similar to the method described in [11], was used to estimate dynamic trends in enzyme activities with a 1-hour resolution. This optimization was necessary, because the ceramide system is underdetermined for each of the 1-hour intervals that span the experimental time period. Thus, the system itself permits infinitely many solutions regarding admissible sets of enzyme activities, while the optimization has the purpose of selecting among the many solutions those that are in some sense superior. Specifically, we created artificial variability of the “data” by introducing 10% upper and lower bounds to the mean time courses, as discussed above. We then randomly selected initial guesses of the enzyme activities, which in each case led to a much smaller and well-interpolated solution space. The subsequent model simulations, starting with randomized initial enzyme activity setting, were not forced to fit particular data points but to yield an ensemble of solutions within the ±10% variability range. This introduction of artificial variability provided us with a significant gain in confidence regarding the estimated enzyme activities. A flowchart of the process is presented in Fig 13.

The results of the piecewise optimization throughout the 20 hours of hydroxyurea exposure were stored for further analysis. It turned out that many dynamic trends in enzyme activities were almost linear or piecewise linear from 0–3 hours and from 3–20 hours, which may be due to the fact that only three measurement time points are available data. Nonetheless, we utilized this observation and computed linear regression lines from 0–3 hours, 3–20 hours, and also 0–20 hours of the estimated enzyme activities and recorded the slopes of these functions as indications of trends.

### Dynamic Flux Re-estimation & Mass Flow Analysis

The piecewise optimization returned rate constants in one-hour intervals, but did not directly provide estimations of fluxes, which we needed for the mass flow analysis. Thus, fluxes were estimated as follows. Rate constants were estimated in a stepwise manner for each 1-hour interval. However, the fluxes are not even constant within each 1-hour time interval because the substrate concentrations change. To obtain representative flux estimates for each 1-hour interval, we split each interval into 100 segments and calculated 100 sequential flux values per hour. Estimates of fluxes in each hourly interval were then represented as averages of these 100 flux segments.

For the mass flow analysis, we further averaged these fluxes from 0–3 hours, 3–20 hours, and 0–20 hours. Mass flow distributions for these time intervals were then estimated through appropriate weighting: [average of fluxes from 0–3 hours]*[3 hours], [average of fluxes from 3–20 hours]*[17 hours] and [average of fluxes from 0–20 hours]*[20 hours]. These mass flows were normalized and are displayed in Figs 9 and 10 of the *Results* section.

## Supporting Information

S1 File Additional details regarding data, model equations, and our optimization approach. (DOCX)

S2 File Details regarding the computed fold changes in enzyme activities as well as the implementation of our methods in the format of Matlab code. (DOCX)
