# Supplementary material for: Analysis of the Involvement of Different Ceramide Variants in the Response to Hydroxyurea Stress in Baker's Yeast
Source: PLoS One. 2016 Jan 19;11(1):e0146839. doi: 10.1371/journal.pone.0146839 (PMC4718512; doi:10.1371/journal.pone.0146839)
Supplement: S1 File — (DOCX) [file pone.0146839.s001.docx]

**Supporting Information 1:**

**Data, Model Equations, and Optimization Approach**

**Analysis of the Involvement of Different Ceramide Variants in the Response to Hydroxyurea Stress in Baker’s Yeast**

Po-Wei Chen, Luis L. Fonseca, Yusuf A. Hannun and Eberhard O. Voit

**Data**

The fold changes in ceramide and sphingosine concentrations under exposure of hydroxyurea were published by our collaborators. The measurements used in our model are included in Table S1.

**Table S1: Metabolites and Corresponding Fold Change Measurements**

|  | C14 DHC | C16 DHC | C18 DHC | C18:1 DHC | C20 DHC | C20:1 DHC | C22 DHC | C22:1 DHC | C24 DHC | C24:1 DHC | C26 DHC | C26:1 DHC |
| --- | --- | --- | --- | --- | --- | --- | --- | --- | --- | --- | --- | --- |
| 3 hours | **0.8** | **0.66** | **0.41** | **0.36** | **0.36** | **0.22** | **0.69** | **0.43** | **0.45** | **0.36** | **0.87** | **0.89** |
|  | **1.46** | **1.99** | **1.56** | **1.24** | **1.04** | **1.12** | **1.15** | **0.71** | **1.46** | **0.94** | **1.99** | **1.9** |
| 20 hours | **1.77** | **1.74** | **0.93** | **0.68** | **1.34** | **0.92** | **1.54** | **1.17** | **1.34** | **0.55** | **3.6** | **2** |
|  | **0.79** | **1.88** | **1.92** | **0.96** | **0.93** | **0.68** | **0.95** | **0.51** | **1.92** | **0.64** | **2.2** | **2.68** |

|  | C14 PHC | C16 PHC | C18 PHC | C18:1 PHC | C20 PHC | C20:1 PHC | C22 PHC | C22:1 PHC | C24 PHC | C24:1 PHC | C26 PHC | C26:1 PHC |
| --- | --- | --- | --- | --- | --- | --- | --- | --- | --- | --- | --- | --- |
| 3 hours | **1.25** | **2.22** | **2.35** | **0.66** | **1.37** | **0.94** | **0.88** | **1.15** | **1.23** | **0.4** | **2.5** | **0.75** |
|  | **1.69** | **1.43** | **1.24** | **0.65** | **1.18** | **0.52** | **1** | **0.39** | **1.91** | **0.3** | **6.24** | **2.54** |
| 20 hours | **2.9** | **4.4** | **9.52** | **4** | **8.77** | **2.2** | **2.8** | **4.6** | **2.6** | **0.28** | **13** | **2** |
|  | **1.9** | **3** | **11.26** | **4.46** | **1.99** | **0.92** | **1.67** | **0.92** | **2.7** | **2.47** | **5.55** | **4** |

|  | DHS | PHS | DHS1p | PHS1p |
| --- | --- | --- | --- | --- |
| 3 hours | **0.59** | **1.23** | **0.11** | **0.27** |
|  | **1.02** | **1.9** | **0.63** | **1.88** |
| 20 hours | **1.59** | **1.47** | **0.6** | **0.37** |
|  | **3.72** | **1** | **5.02** | **0.5** |

**ODE Model**

This section describes details regarding the ODE model used in this paper. Dependent variables (metabolites) were denoted as $X_{i}$, while enzyme activities were subsumed into 137 rate constants and labeled as $\gamma_{j}$, where $i,j$ represent indices of metabolites and enzyme activities in each reaction. The index of a rate constant, $j$, corresponds to the index of the associated fluxes, *V*, in Figure 2 of the main text. Names and notations of all metabolites are exhibited in Table S2. Rate constants (enzyme activities) were estimated by our approach and the plots were included in the main text.

**Table 2. Names and Symbols of Metabolites**

|  | **DHC species** |  | **PHC species** |  | **Sphingosines** |  | **Fatty acyl CoAs** |  | **Complex sphingolipid** |
| --- | --- | --- | --- | --- | --- | --- | --- | --- | --- |
| $X_{1}$ | C14 DHC | $X_{13}$ | C14 PHC | $X_{25}$ | DHS | $X_{29}$ | C14 FAC | $X_{41}$ | IPC |
| $X_{2}$ | C16 DHC | $X_{14}$ | C16 PHC | $X_{26}$ | PHS | $X_{30}$ | C16 FAC | $X_{42}$ |  |
| $X_{3}$ | C18 DHC | $X_{15}$ | C18 PHC | $X_{27}$ | DHS1p | $X_{31}$ | C18 FAC |  |  |
| $X_{4}$ | C18:1 DHC | $X_{16}$ | C18:1 PHC | $X_{28}$ | PHS1p | $X_{32}$ | C18:1 FAC |  |  |
| $X_{5}$ | C20 DHC | $X_{17}$ | C20 PHC |  |  | $X_{33}$ | C20 FAC |  |  |
| $X_{6}$ | C20:1 DHC | $X_{18}$ | C20:1 PHC |  |  | $X_{34}$ | C20:1 FAC |  |  |
| $X_{7}$ | C22 DHC | $X_{19}$ | C22 PHC |  |  | $X_{35}$ | C22 FAC |  |  |
| $X_{8}$ | C22:1 DHC | $X_{20}$ | C22:1 PHC |  |  | $X_{36}$ | C22:1 FAC |  |  |
| $X_{9}$ | C24 DHC | $X_{21}$ | C24 PHC |  |  | $X_{37}$ | C24 FAC |  |  |
| $X_{10}$ | C24:1 DHC | $X_{22}$ | C24:1 PHC |  |  | $X_{38}$ | C24:1 FAC |  |  |
| $X_{11}$ | C26 DHC | $X_{23}$ | C26 PHC |  |  | $X_{39}$ | C26 FAC |  |  |
| $X_{12}$ | C26:1 DHC | $X_{24}$ | C26:1 PHC |  |  | $X_{40}$ | C26:1 FAC |  |  |

**Equations**

**DHC Species**

$$\dot{X_{1}}=\gamma_{1}X_{29}X_{25}+\gamma_{2}X_{41}-\gamma_{3}X_{1}-\gamma_{4}X_{1}-\gamma_{5}X_{1}$$

$$\dot{X_{2}}=\gamma_{6}X_{30}X_{25}+\gamma_{7}X_{41}-\gamma_{8}X_{2}-\gamma_{9}X_{2}-\gamma_{10}X_{2}$$

$$\dot{X_{3}}=\gamma_{11}X_{31}X_{25}+\gamma_{12}X_{41}-\gamma_{13}X_{3}-\gamma_{14}X_{3}-\gamma_{15}X_{3}$$

$$\dot{X_{4}}=\gamma_{16}X_{32}X_{25}+\gamma_{17}X_{41}-\gamma_{18}X_{4}-\gamma_{19}X_{4}-\gamma_{20}X_{4}$$

$$\dot{X_{5}}=\gamma_{21}X_{33}X_{25}+\gamma_{22}X_{41}-\gamma_{23}X_{5}-\gamma_{24}X_{5}-\gamma_{25}X_{5}$$

$$\dot{X_{6}}=\gamma_{26}X_{34}X_{25}+\gamma_{27}X_{41}-\gamma_{28}X_{6}-\gamma_{29}X_{6}-\gamma_{30}X_{6}$$

$$\dot{X_{7}}=\gamma_{31}X_{35}X_{25}+\gamma_{32}X_{41}-\gamma_{33}X_{7}-\gamma_{34}X_{7}-\gamma_{35}X_{7}$$

$$\dot{X_{8}}=\gamma_{36}X_{36}X_{25}+\gamma_{37}X_{41}-\gamma_{38}X_{8}-\gamma_{39}X_{8}-\gamma_{40}X_{8}$$

$$\dot{X_{9}}=\gamma_{41}X_{37}X_{25}+\gamma_{42}X_{41}-\gamma_{43}X_{9}-\gamma_{44}X_{9}-\gamma_{45}X_{9}$$

$$\dot{X_{10}}=\gamma_{46}X_{38}X_{25}+\gamma_{47}X_{41}-\gamma_{48}X_{10}-\gamma_{49}X_{10}-\gamma_{50}X_{10}$$

$$\dot{X_{11}}=\gamma_{51}X_{39}X_{25}+\gamma_{52}X_{41}-\gamma_{53}X_{11}-\gamma_{54}X_{11}-\gamma_{55}X_{11}$$

$$\dot{X_{12}}=\gamma_{12,1}X_{56}X_{25}+\gamma_{57}X_{41}-\gamma_{58}X_{12}-\gamma_{59}X_{12}-\gamma_{60}X_{12}$$

**PHC Species**

$$\dot{X_{13}}=\gamma_{61}X_{29}X_{26}+\gamma_{62}X_{42}+\gamma_{4}X_{1}-\gamma_{63}X_{13}-\gamma_{64}X_{13}$$

$$\dot{X_{14}}=\gamma_{65}X_{30}X_{26}+\gamma_{66}X_{42}+\gamma_{9}X_{2}-\gamma_{67}X_{14}-\gamma_{68}X_{14}$$

$$\dot{X_{15}}=\gamma_{69}X_{31}X_{26}+\gamma_{70}X_{42}+\gamma_{14}X_{3}-\gamma_{71}X_{15}-\gamma_{72}X_{15}$$

$$\dot{X_{16}}=\gamma_{73}X_{32}X_{26}+\gamma_{74}X_{42}+\gamma_{19}X_{4}-\gamma_{75}X_{16}-\gamma_{76}X_{16}$$

$$\dot{X_{17}}=\gamma_{77}X_{33}X_{26}+\gamma_{78}X_{42}+\gamma_{24}X_{5}-\gamma_{79}X_{17}-\gamma_{80}X_{17}$$

$$\dot{X_{18}}=\gamma_{81}X_{34}X_{26}+\gamma_{82}X_{42}+\gamma_{29}X_{6}-\gamma_{83}X_{18}-\gamma_{84}X_{18}$$

$$\dot{X_{19}}=\gamma_{85}X_{35}X_{26}+\gamma_{86}X_{42}+\gamma_{34}X_{7}-\gamma_{87}X_{19}-\gamma_{88}X_{19}$$

$$\dot{X_{20}}=\gamma_{89}X_{36}X_{26}+\gamma_{90}X_{42}+\gamma_{39}X_{8}-\gamma_{91}X_{20}-\gamma_{92}X_{20}$$

$$\dot{X_{21}}=\gamma_{93}X_{37}X_{26}+\gamma_{94}X_{42}+\gamma_{44}X_{9}-\gamma_{95}X_{21}-\gamma_{96}X_{21}$$

$$\dot{X_{22}}=\gamma_{97}X_{38}X_{26}+\gamma_{98}X_{42}+\gamma_{49}X_{10}-\gamma_{99}X_{22}-\gamma_{100}X_{22}$$

$$\dot{X_{23}}=\gamma_{101}X_{39}X_{26}+\gamma_{102}X_{42}+\gamma_{54}X_{11}-\gamma_{103}X_{23}-\gamma_{104}X_{23}$$

$$\dot{X_{24}}=\gamma_{105}X_{40}X_{26}+\gamma_{106}X_{42}+\gamma_{59}X_{12}-\gamma_{107}X_{24}-\gamma_{108}X_{24}$$

**Sphingolipids**

$$\dot{X_{25}}=\gamma_{109}+\gamma_{110}X_{27}-\gamma_{111}X_{25}-\gamma_{112}X_{25}+\gamma_{3}X_{1}+\gamma_{8}X_{2}+\gamma_{13}X_{3}+\gamma_{18}X_{4}+\gamma_{23}X_{5}+\gamma_{28}X_{6}+\gamma_{33}X_{7}+\gamma_{38}X_{8}+\gamma_{43}X_{9}+\gamma_{48}X_{10}+\gamma_{53}X_{11}+\gamma_{58}X_{12}-\gamma_{1}X_{25}X_{29}-\gamma_{6}X_{25}X_{30}-\gamma_{11}X_{25}X_{31}-\gamma_{16}X_{25}X_{32}-\gamma_{21}X_{25}X_{33}-\gamma_{26}X_{25}X_{34}-\gamma_{31}X_{25}X_{35}-\gamma_{36}X_{25}X_{36}-\gamma_{41}X_{25}X_{37}-\gamma_{46}X_{25}X_{38}-\gamma_{51}X_{25}X_{39}-\gamma_{56}X_{25}X_{40}$$

$$\dot{X_{26}}=\gamma_{112}X_{25}+\gamma_{113}X_{28}-\gamma_{114}X_{26}+\gamma_{63}X_{13}+\gamma_{67}X_{13}+\gamma_{71}X_{13}+\gamma_{75}X_{13}+\gamma_{79}X_{13}+\gamma_{83}X_{13}+\gamma_{87}X_{13}+\gamma_{91}X_{13}+\gamma_{95}X_{13}+\gamma_{99}X_{13}+\gamma_{103}X_{13}+\gamma_{107}X_{13}-\gamma_{61}X_{26}X_{29}-\gamma_{65}X_{26}X_{30}-\gamma_{69}X_{26}X_{31}-\gamma_{73}X_{26}X_{32}-\gamma_{77}X_{26}X_{33}-\gamma_{81}X_{26}X_{34}-\gamma_{85}X_{26}X_{35}-\gamma_{89}X_{26}X_{36}-\gamma_{93}X_{26}X_{37}-\gamma_{97}X_{26}X_{38}-\gamma_{101}X_{26}X_{39}-\gamma_{105}X_{26}X_{40}$$

$$\dot{X_{27}}=\gamma_{111}X_{25}-\gamma_{110}X_{27}-\gamma_{115}X_{27}$$

$$\dot{X_{28}}=\gamma_{114}X_{26}-\gamma_{113}X_{28}-\gamma_{116}X_{28}$$

**Fatty Acyl CoAs**

$$\dot{X_{29}}=\gamma_{117}+\gamma_{3}X_{1}+\gamma_{63}X_{13}-\gamma_{1}X_{25}X_{29}-\gamma_{61}X_{26}X_{29}-\gamma_{118}X_{29}-\gamma_{136}X_{29}$$

$$\dot{X_{30}}=\gamma_{118}X_{29}+\gamma_{8}X_{2}+\gamma_{67}X_{14}-\gamma_{6}X_{25}X_{30}-\gamma_{65}X_{26}X_{30}-\gamma_{119}X_{30}-\gamma_{137}X_{30}$$

$$\dot{X_{31}}=\gamma_{119}X_{30}+\gamma_{13}X_{3}+\gamma_{71}X_{15}-\gamma_{11}X_{25}X_{31}-\gamma_{69}X_{26}X_{31}-\gamma_{120}X_{31}-\gamma_{131}X_{31}$$

$$\dot{X_{32}}=\gamma_{131}X_{31}+\gamma_{18}X_{4}+\gamma_{75}X_{16}-\gamma_{16}X_{25}X_{32}-\gamma_{73}X_{26}X_{32}+\gamma_{125}-\gamma_{126}X_{32}$$

$$\dot{X_{33}}=\gamma_{120}X_{31}+\gamma_{23}X_{5}+\gamma_{79}X_{17}-\gamma_{21}X_{25}X_{33}-\gamma_{77}X_{26}X_{33}-\gamma_{121}X_{33}-\gamma_{132}X_{33}$$

$$\dot{X_{34}}=\gamma_{132}X_{33}+\gamma_{28}X_{6}+\gamma_{83}X_{18}-\gamma_{26}X_{25}X_{34}-\gamma_{81}X_{26}X_{34}+\gamma_{126}X_{32}-\gamma_{127}X_{34}$$

$$\dot{X_{35}}=\gamma_{121}X_{33}+\gamma_{33}X_{7}+\gamma_{87}X_{19}-\gamma_{31}X_{25}X_{35}-\gamma_{85}X_{26}X_{35}-\gamma_{122}X_{35}-\gamma_{133}X_{35}$$

$$\dot{X_{36}}=\gamma_{133}X_{35}+\gamma_{38}X_{8}+\gamma_{91}X_{20}-\gamma_{36}X_{25}X_{36}-\gamma_{89}X_{26}X_{36}+\gamma_{127}X_{34}-\gamma_{128}X_{36}$$

$$\dot{X_{37}}=\gamma_{122}X_{35}+\gamma_{43}X_{9}+\gamma_{95}X_{21}-\gamma_{41}X_{25}X_{37}-\gamma_{93}X_{26}X_{37}-\gamma_{123}X_{37}-\gamma_{134}X_{37}$$

$$\dot{X_{38}}=\gamma_{134}X_{37}+\gamma_{48}X_{10}+\gamma_{99}X_{22}-\gamma_{46}X_{25}X_{38}-\gamma_{97}X_{26}X_{38}+\gamma_{128}X_{36}-\gamma_{129}X_{38}$$

$$\dot{X_{39}}=\gamma_{123}X_{37}+\gamma_{53}X_{11}+\gamma_{103}X_{23}-\gamma_{51}X_{25}X_{39}-\gamma_{101}X_{26}X_{39}-\gamma_{124}X_{39}-\gamma_{135}X_{39}$$

$$\dot{X_{40}}=\gamma_{135}X_{39}+\gamma_{58}X_{12}+\gamma_{107}X_{24}-\gamma_{56}X_{25}X_{40}-\gamma_{105}X_{26}X_{40}+\gamma_{129}X_{39}-\gamma_{130}X_{40}$$

**Task Statement for each Iteration of the Piecewise Optimization Approach**

Piecewise optimization was performed to estimate enzyme activities, which are subsumed in the rate constants. Figure 13 in the main text provides an overview of the procedures used. For each time point along the trajectories of ceramide concentrations, exhibited as fold changes, a piecewise optimization approach was performed. Specifically, the optimization task of computing the enzyme activities (rate constants) followed the procedure below:

$$\min_{E(t)} \sum_{i\in N} {(\frac{IntExp\left( t,i \right)-F(E\left( t \right),X_{i}(t-1))}{IntExp(t,i)})}^{2}$$

where:

$E(t)$ is the vector of enzyme activities at time $t$;

$IntExp\left( t,i \right)$ is the fitting target, randomly sampled from the 20% range of the interpolated data at each time point $t$ and for the $i^{th}$ ceramide/sphingosine species;

$N$ is the total number of ceramide and sphingosine species;

$F$ is a function containing the ODE solution that produces the simulated fold change concentration of ceramides and sphingosines; and

$X_{i}(t-1)$ is the simulated concentration at the previous time point, which is used as the starting point for the next simulation with the ODE solver toward the subsequent time point.
