# Supplementary material for: Analysis of the Involvement of Different Ceramide Variants in the Response to Hydroxyurea Stress in Baker's Yeast
Source: PLoS One. 2016 Jan 19;11(1):e0146839. doi: 10.1371/journal.pone.0146839 (PMC4718512; doi:10.1371/journal.pone.0146839)
Supplement: S2 File — (DOCX) [file pone.0146839.s002.docx]

**Supporting Information 2:**

**Computed Fold Changes in Enzymes and Matlab Implementation**

**Analysis of the Involvement of Different Ceramide Variants in the Response to Hydroxyurea Stress in Baker’s Yeast**

Po-Wei Chen, Luis L. Fonseca, Yusuf A. Hannun and Eberhard O. Voit

**Table S2 – Fold Changes in Enzyme Activities**

**Ceramide Synthase Activities – DHC side**

| **Hr** | **Ceramide Substrates** | | | | | | | | | | | |
| --- | --- | --- | --- | --- | --- | --- | --- | --- | --- | --- | --- | --- |
|  | **C14 DHC** | **C16 DHC** | **C18 DHC** | **C18:1 DHC** | **C20 DHC** | **C20:1 DHC** | **C22 DHC** | **C22:1 DHC** | **C24 DHC** | **C24:1 DHC** | **C26 DHC** | **C26:1 DHC** |
| **1** | 1.14 | 2 | 1.77 | 1.19 | 1.33 | 0.98 | 1.11 | 1.03 | 1.86 | 1.58 | 1.55 | 1.552 |
| **2** | 1.50 | 1.31 | 1 | 1.80 | 1.34 | 1.4 | 1.82 | 1.62 | 0.98 | 0.53 | 1.43 | 1.46 |
| **3** | 2.19 | 1.2 | 1.02 | 1.03 | 0.56 | 1.11 | 1.23 | 1.02 | 1.38 | 1.6 | 1.34 | 1.2 |
| **4** | 2.30 | 1.51 | 0.68 | 1.07 | 1.13 | 1.6 | 1 | 1.15 | 1.13 | 0.77 | 1.56 | 1.65 |
| **5** | 1.65 | 1.14 | 1.74 | 1.62 | 1.3 | 0.55 | 1.56 | 1.39 | 1.89 | 1.44 | 1.94 | 1.18 |
| **6** | 1.91 | 2.37 | 1.790 | 0.78 | 0.67 | 0.66 | 1.83 | 0.88 | 1.36 | 1.54 | 1.69 | 1.56 |
| **7** | 1.72 | 2.08 | 1.29 | 0.9 | 1.43 | 1.15 | 1.15 | 0.7 | 1.9 | 0.85 | 1.56 | 1.77 |
| **8** | 1.89 | 1.33 | 1.85 | 1.41 | 0.94 | 0.84 | 0.43 | 0.37 | 1.31 | 1.37 | 2.14 | 1.67 |
| **9** | 2.29 | 1.8 | 1.24 | 1.17 | 0.36 | 1.28 | 1.87 | 0.79 | 1.03 | 0.5 | 1.45 | 1.38 |
| **10** | 0.96 | 2.21 | 1.44 | 0.75 | 0.94 | 1.2 | 1.16 | 0.26 | 0.66 | 0.35 | 1.2 | 0.96 |
| **11** | 1.4 | 1.4 | 1.99 | 1.63 | 1.03 | 0.74 | 1.53 | 0.84 | 0.67 | 0.78 | 2.08 | 1.52 |
| **12** | 0.92 | 1.56 | 1.62 | 1.55 | 0.48 | 0.82 | 1.82 | 0.62 | 1.42 | 1.02 | 2.12 | 1.14 |
| **13** | 1.37 | 2.21 | 0.75 | 0.81 | 0.93 | 0.98 | 1.36 | 0.25 | 1.56 | 0.39 | 2.07 | 1.21 |
| **14** | 1.24 | 2.17 | 1.29 | 1.08 | 0.8 | 1.05 | 1.06 | 0.93 | 0.89 | 0.62 | 1.72 | 2 |
| **15** | 1.26 | 1.77 | 1.29 | 1.08 | 0.95 | 1.19 | 1.37 | 0.35 | 1.51 | 0.41 | 1.94 | 1.08 |
| **16** | 1.27 | 1.58 | 1.05 | 0.91 | 0.63 | 1.04 | 1.66 | 0.87 | 1.39 | 0.21 | 1.22 | 1.32 |
| **17** | 1.18 | 1.21 | 1.45 | 1.01 | 1.24 | 0.71 | 0.8 | 0.48 | 1.43 | 0.2 | 1.54 | 1.34 |
| **18** | 1.05 | 1.64 | 1.39 | 1.1 | 0.49 | 1.22 | 0.94 | 0.78 | 1.64 | 0.45 | 1.36 | 0.93 |
| **19** | 1.35 | 1.66 | 0.92 | 0.44 | 1.78 | 0.67 | 1.71 | 0.31 | 1.71 | 0.54 | 1.7 | 1.37 |
| **20** | 1.48 | 0.88 | 1.31 | 0.46 | 0.2 | 1.13 | 1.48 | 1.03 | 1.72 | 0.47 | 1.29 | 1.36 |

**Ceramide Synthase Activities – PHC side**

| **Hr** | **Ceramide Substrates** | | | | | | | | | | | |
| --- | --- | --- | --- | --- | --- | --- | --- | --- | --- | --- | --- | --- |
|  | **C14 PHC** | **C16 PHC** | **C18 PHC** | **C18:1 PHC** | **C20 PHC** | **C20:1 PHC** | **C22 PHC** | **C22:1 PHC** | **C24 PHC** | **C24:1 PHC** | **C26 PHC** | **C26:1 PHC** |
| **1** | 1.11 | 1.02 | 1.56 | 1.55 | 1.98 | 1.25 | 1.09 | 1.08 | 1.32 | 0.58 | 1.35 | 0.72 |
| **2** | 1.84 | 1.22 | 1.45 | 1.28 | 1.54 | 1.27 | 1.21 | 0.73 | 2.36 | 0.54 | 2.31 | 1.22 |
| **3** | 1.32 | 1.6 | 0.92 | 1.27 | 1.16 | 0.64 | 1.16 | 1.43 | 1.15 | 0.47 | 1.98 | 1.1 |
| **4** | 1.87 | 2.05 | 2.12 | 1.08 | 1.09 | 0.6 | 1.04 | 0.56 | 1.67 | 0.21 | 1.88 | 1.74 |
| **5** | 1.55 | 2.11 | 1.48 | 1.12 | 1.36 | 0.62 | 1.07 | 0.88 | 0.91 | 0.38 | 1.51 | 1.95 |
| **6** | 1.73 | 1.01 | 2 | 0.8 | 1.43 | 0.71 | 1.05 | 0.69 | 1.47 | 0.39 | 2.08 | 0.84 |
| **7** | 1.41 | 2.27 | 0.93 | 0.78 | 1.84 | 0.25 | 1.12 | 0.51 | 1.7 | 0.19 | 1.1 | 1.64 |
| **8** | 1.16 | 1.2 | 2.35 | 1.5 | 1.56 | 0.74 | 1.19 | 1.49 | 1.55 | 0.22 | 1.91 | 1.53 |
| **9** | 1.62 | 2.14 | 2.31 | 0.61 | 1.42 | 0.68 | 0.69 | 0.62 | 0.81 | 0.28 | 2.23 | 1.58 |
| **10** | 1.44 | 2.46 | 1.72 | 1.06 | 1.11 | 0.63 | 1.76 | 1.27 | 1.36 | 0.27 | 1.68 | 1.93 |
| **11** | 1.87 | 1.46 | 2.56 | 1.09 | 1.34 | 0.91 | 1.56 | 1.55 | 1.84 | 0.38 | 1.72 | 1.54 |
| **12** | 2.2 | 1.93 | 1.63 | 1.55 | 2.11 | 0.8 | 1.4 | 1.47 | 1.41 | 0.46 | 2.24 | 2.17 |
| **13** | 1.17 | 2.44 | 1.88 | 1.13 | 2.61 | 0.62 | 0.56 | 1.24 | 2.52 | 0.71 | 2.69 | 1.87 |
| **14** | 2.17 | 1.97 | 2.3 | 1.48 | 1.87 | 0.7 | 1.07 | 1.29 | 1.06 | 0.42 | 2.75 | 0.77 |
| **15** | 1.18 | 0.94 | 1.96 | 1.54 | 1.86 | 1.24 | 1.46 | 0.9 | 1.53 | 0.54 | 2.25 | 1.61 |
| **16** | 1.55 | 1.35 | 2.3 | 1.71 | 1.67 | 1.79 | 1.21 | 1.49 | 1.29 | 1.3 | 2.19 | 1.87 |
| **17** | 1.64 | 1.8 | 2.89 | 0.82 | 1.49 | 0.91 | 1.84 | 1.07 | 0.93 | 0.88 | 2.14 | 1.39 |
| **18** | 1.93 | 1.95 | 2.34 | 1.93 | 1.42 | 1.77 | 2.01 | 1.42 | 1.57 | 1.2 | 3 | 2.23 |
| **19** | 1.56 | 2.48 | 2.43 | 1.83 | 1.26 | 1.9 | 1.8 | 1.53 | 1.71 | 1.44 | 1.65 | 1.89 |
| **20** | 1.86 | 2.55 | 2.43 | 2.24 | 2.83 | 1.57 | 1.92 | 1.19 | 0.78 | 1.29 | 2.13 | 1.91 |

**Dihydroceramidase Activities**

| **Hr** | **Ceramide Substrates** | | | | | | | | | | | |
| --- | --- | --- | --- | --- | --- | --- | --- | --- | --- | --- | --- | --- |
|  | **C14 DHC** | **C16 DHC** | **C18 DHC** | **C18:1 DHC** | **C20 DHC** | **C20:1 DHC** | **C22 DHC** | **C22:1 DHC** | **C24 DHC** | **C24:1 DHC** | **C26 DHC** | **C26:1 DHC** |
| **1** | 1.17 | 0.69 | 1.5 | 1.44 | 1.05 | 1.54 | 1.78 | 1.66 | 1.9 | 1.43 | 1.61 | 0.98 |
| **2** | 1.09 | 0.81 | 1.58 | 1.77 | 1.31 | 1.14 | 1.38 | 0.57 | 1.04 | 0.72 | 0.95 | 0.5 |
| **3** | 1.54 | 1.25 | 1.23 | 1.53 | 1.01 | 0.59 | 1.61 | 1.29 | 1.67 | 1.59 | 0.31 | 0.73 |
| **4** | 1.39 | 0.57 | 1.81 | 0.92 | 1.75 | 1.38 | 0.83 | 1.05 | 0.81 | 1.45 | 0.89 | 0.49 |
| **5** | 0.83 | 0.52 | 1.26 | 1.41 | 1.54 | 0.74 | 1.01 | 1.56 | 0.58 | 1.57 | 1.11 | 1.16 |
| **6** | 0.45 | 1.07 | 1.38 | 0.9 | 0.96 | 0.75 | 1.19 | 1.98 | 1.23 | 1.63 | 0.17 | 0.26 |
| **7** | 1.23 | 1.28 | 0.59 | 1.5 | 2.16 | 0.93 | 0.99 | 1.48 | 1.37 | 1.08 | 0.95 | 1.12 |
| **8** | 1.47 | 0.65 | 0.88 | 1.31 | 1.75 | 1.52 | 1.04 | 2.44 | 1.46 | 2.01 | 0.72 | 0.57 |
| **9** | 1.1 | 1.33 | 0.51 | 1.27 | 0.99 | 2.15 | 1.47 | 1.68 | 1.31 | 1.74 | 0.34 | 1.01 |
| **10** | 0.57 | 0.96 | 1.23 | 1.2 | 1.51 | 1.48 | 1.34 | 1.54 | 0.52 | 1.92 | 0.71 | 0.48 |
| **11** | 1 | 1.14 | 0.59 | 1.89 | 1.81 | 1.86 | 1.64 | 1.78 | 0.89 | 2.45 | 1.01 | 0.71 |
| **12** | 0.51 | 1.15 | 1.65 | 2.32 | 2.14 | 2.51 | 1.21 | 1.94 | 0.72 | 2.97 | 0.82 | 0.27 |
| **13** | 1.41 | 1.07 | 1.2 | 1.2 | 1.48 | 2.74 | 0.87 | 1.76 | 1.33 | 2.19 | 0.97 | 1.24 |
| **14** | 0.79 | 0.52 | 1.57 | 1.53 | 1.18 | 1.68 | 1.41 | 2.78 | 1.02 | 1.49 | 0.68 | 0.96 |
| **15** | 0.73 | 1.14 | 2 | 1.96 | 1.76 | 2.61 | 1.41 | 1.49 | 0.86 | 1.66 | 0.69 | 0.79 |
| **16** | 1.58 | 1.11 | 1.99 | 1.45 | 1.37 | 1.2 | 1.5 | 1.81 | 1.64 | 2.29 | 0.96 | 1.59 |
| **17** | 1.26 | 1.6 | 0.95 | 1.58 | 1.63 | 1.61 | 1.56 | 1.07 | 1.37 | 1.31 | 0.82 | 0.94 |
| **18** | 1.81 | 1.18 | 2.32 | 2.11 | 1.3 | 1.97 | 1.55 | 1.23 | 1.39 | 1.16 | 1.12 | 0.46 |
| **19** | 1.77 | 1.75 | 1.68 | 2.24 | 1.69 | 1.91 | 1.36 | 1.74 | 1.55 | 1.85 | 1.26 | 1.41 |
| **20** | 1.61 | 0.56 | 1.42 | 1.2 | 1.02 | 2.34 | 1.81 | 2.06 | 1.64 | 0.94 | 0.69 | 1.08 |

**Phytoceramidase Activities**

| **Hr** | **Ceramide Substrates** | | | | | | | | | | | |
| --- | --- | --- | --- | --- | --- | --- | --- | --- | --- | --- | --- | --- |
|  | **C14 PHC** | **C16 PHC** | **C18 PHC** | **C18:1 PHC** | **C20 PHC** | **C20:1 PHC** | **C22 PHC** | **C22:1 PHC** | **C24 PHC** | **C24:1 PHC** | **C26 PHC** | **C26:1 PHC** |
| **1** | 0.52 | 1.37 | 1.21 | 2.16 | 1.62 | 1.81 | 1.7 | 1.69 | 1.23 | 0.81 | 0.43 | 1.41 |
| **2** | 1.39 | 0.73 | 0.94 | 1.36 | 1.16 | 2.16 | 1.1 | 1.64 | 1.34 | 0.89 | 0.62 | 0.96 |
| **3** | 1.19 | 0.39 | 0.98 | 2.22 | 1.32 | 1.26 | 1.99 | 1.68 | 0.61 | 1.46 | 0.97 | 1.31 |
| **4** | 1.43 | 1.82 | 1.7 | 2.44 | 1.2 | 1.79 | 2.05 | 1.41 | 1.45 | 2.24 | 0.7 | 1.19 |
| **5** | 0.87 | 1.61 | 1.05 | 1.73 | 0.96 | 1.07 | 1.97 | 1.67 | 1.48 | 2.6 | 0.47 | 0.9 |
| **6** | 1.61 | 0.86 | 1.02 | 1.7 | 1.6 | 1.84 | 1.37 | 1.94 | 1.07 | 2.49 | 0.55 | 0.76 |
| **7** | 1.35 | 1.61 | 0.89 | 1.92 | 1.64 | 1.55 | 2 | 1.72 | 1.11 | 1.63 | 0.25 | 1.65 |
| **8** | 0.38 | 0.54 | 0.75 | 1.56 | 1.29 | 1.9 | 1.19 | 2.74 | 1.21 | 2.25 | 0.49 | 0.99 |
| **9** | 1.58 | 1.28 | 0.99 | 1.15 | 1.36 | 1.93 | 1.76 | 2.06 | 0.87 | 2.11 | 0.63 | 1.66 |
| **10** | 1.43 | 1.06 | 0.53 | 1.67 | 0.51 | 1.38 | 1.53 | 2.31 | 1.38 | 2.8 | 0.27 | 1.03 |
| **11** | 1.16 | 0.72 | 1.15 | 0.7 | 0.65 | 2.51 | 1.31 | 1.93 | 1.18 | 1.68 | 0.23 | 1.26 |
| **12** | 1.15 | 0.94 | 0.34 | 1.29 | 0.94 | 1.95 | 0.73 | 1.54 | 1.05 | 1.4 | 0.5 | 1.28 |
| **13** | 0.76 | 1.46 | 0.66 | 1.25 | 0.77 | 1.73 | 1.34 | 2.23 | 1.06 | 1.96 | 1.06 | 1.63 |
| **14** | 1.73 | 0.83 | 0.9 | 0.56 | 1.43 | 1.83 | 1.1 | 1.65 | 0.73 | 2.14 | 0.22 | 0.67 |
| **15** | 0.98 | 0.72 | 0.45 | 1.34 | 0.65 | 2.25 | 1.28 | 0.64 | 1.06 | 1.27 | 0.35 | 0.98 |
| **16** | 0.95 | 0.73 | 0.41 | 0.78 | 0.6 | 2.28 | 1.3 | 0.98 | 0.83 | 1.84 | 0.28 | 0.51 |
| **17** | 0.74 | 0.72 | 0.56 | 0.61 | 0.47 | 1.38 | 1.57 | 1.18 | 0.68 | 1.29 | 0.57 | 1.01 |
| **18** | 1.54 | 0.65 | 0.51 | 0.7 | 0.52 | 1.55 | 1.15 | 0.8 | 1.3 | 1.87 | 0.43 | 1.39 |
| **19** | 0.64 | 1.03 | 0.24 | 0.83 | 0.5 | 1.7 | 1 | 0.42 | 0.97 | 1.55 | 0.27 | 0.8 |
| **20** | 0.91 | 0.95 | 0.92 | 0.6 | 0.58 | 1.38 | 0.89 | 0.76 | 0.51 | 1.61 | 0.39 | 0.96 |

**IPC Synthase Activities – DHC side**

| **Hr** | **Ceramide Substrates** | | | | | | | | | | | |
| --- | --- | --- | --- | --- | --- | --- | --- | --- | --- | --- | --- | --- |
|  | **C14 DHC** | **C16 DHC** | **C18 DHC** | **C18:1 DHC** | **C20 DHC** | **C20:1 DHC** | **C22 DHC** | **C22:1 DHC** | **C24 DHC** | **C24:1 DHC** | **C26 DHC** | **C26:1 DHC** |
| **1** | 1.51 | 1.89 | 1.47 | 1.06 | 1.57 | 1.19 | 0.98 | 1.13 | 1.75 | 1.46 | 0.8 | 1.62 |
| **2** | 0.83 | 0.76 | 1.02 | 1.26 | 2.68 | 1.98 | 1.66 | 2.02 | 1.86 | 1.28 | 1.35 | 1.81 |
| **3** | 1.54 | 0.53 | 0.59 | 1.44 | 1.12 | 2.03 | 1.33 | 2.36 | 1.82 | 1.68 | 1.38 | 0.7 |
| **4** | 1.15 | 0.71 | 1.24 | 2.12 | 0.91 | 1.4 | 1.64 | 1.52 | 1.37 | 1.32 | 0.45 | 1.17 |
| **5** | 1.37 | 0.68 | 0.39 | 1.33 | 2.34 | 1.36 | 1.81 | 1.81 | 1.75 | 2.16 | 1.42 | 0.39 |
| **6** | 1.62 | 0.66 | 1.84 | 1.7 | 1.54 | 1.81 | 1.75 | 1.72 | 1.84 | 2.17 | 1.01 | 0.96 |
| **7** | 1.92 | 0.97 | 2.08 | 1.22 | 1.74 | 2.02 | 0.73 | 1.83 | 1.44 | 2.06 | 0.44 | 0.8 |
| **8** | 1.38 | 1.63 | 1.01 | 1.54 | 1.27 | 2 | 1.35 | 1.91 | 1 | 2.07 | 0.55 | 1.26 |
| **9** | 1.76 | 0.7 | 1.5 | 1.44 | 1.73 | 1.89 | 1.33 | 2.68 | 1.12 | 2.11 | 1.21 | 1.34 |
| **10** | 1.7 | 1.64 | 1.27 | 1.93 | 1.33 | 2.17 | 1.6 | 1.91 | 1.3 | 1.74 | 0.58 | 0.73 |
| **11** | 1.1 | 0.53 | 1.8 | 1.9 | 2.06 | 1.85 | 1.44 | 2.17 | 1.17 | 2.07 | 0.61 | 1.2 |
| **12** | 1.23 | 0.69 | 1.35 | 1.92 | 1.94 | 1.4 | 1.24 | 2.29 | 1.53 | 1.5 | 0.9 | 0.67 |
| **13** | 0.86 | 0.79 | 1.18 | 1.76 | 1.85 | 2.21 | 1.84 | 1.51 | 1.92 | 0.9 | 0.73 | 0.82 |
| **14** | 1.11 | 1.33 | 1.83 | 1.02 | 1.71 | 1.64 | 2.2 | 1.73 | 1.37 | 2.77 | 0.39 | 1.06 |
| **15** | 1.26 | 0.92 | 1.4 | 0.95 | 1.65 | 1.41 | 1.39 | 1.34 | 0.9 | 1.69 | 0.87 | 0.61 |
| **16** | 1.26 | 1.31 | 1.12 | 1.16 | 2.22 | 2.18 | 1.97 | 2.16 | 1.37 | 1.61 | 0.54 | 0.5 |
| **17** | 0.69 | 0.65 | 1.25 | 1.54 | 1.64 | 1.74 | 1.12 | 1.61 | 1.17 | 2.15 | 0.87 | 0.85 |
| **18** | 0.51 | 0.94 | 0.6 | 2.17 | 1.27 | 1.85 | 1.38 | 1.74 | 1.28 | 1.72 | 0.19 | 0.58 |
| **19** | 1.01 | 0.77 | 0.88 | 1.32 | 1.64 | 1.23 | 1.34 | 0.74 | 1.34 | 2.04 | 0.53 | 0.73 |
| **20** | 0.81 | 1.11 | 0.74 | 0.74 | 1.81 | 1.72 | 2.04 | 1.62 | 0.92 | 2.05 | 0.58 | 0.53 |

**IPC Synthase Activities – PHC side**

| **Hr** | **Ceramide Substrates** | | | | | | | | | | | |
| --- | --- | --- | --- | --- | --- | --- | --- | --- | --- | --- | --- | --- |
|  | **C14 PHC** | **C16 PHC** | **C18 PHC** | **C18:1 PHC** | **C20 PHC** | **C20:1 PHC** | **C22 PHC** | **C22:1 PHC** | **C24 PHC** | **C24:1 PHC** | **C26 PHC** | **C26:1 PHC** |
| **1** | 1.58 | 0.87 | 0.96 | 1.24 | 1.09 | 1.35 | 1.15 | 1.34 | 1.82 | 1.44 | 1.07 | 1.4 |
| **2** | 1.31 | 0.98 | 0.95 | 1.31 | 0.67 | 1 | 2.15 | 1.13 | 1.12 | 1.56 | 0.33 | 0.81 |
| **3** | 1.08 | 1.29 | 0.76 | 2.02 | 0.76 | 1.74 | 1.16 | 2.07 | 1.2 | 1.72 | 0.17 | 1.19 |
| **4** | 1.54 | 0.39 | 1.01 | 2.27 | 0.62 | 1.1 | 1.7 | 0.96 | 0.98 | 1.68 | 0.51 | 1.24 |
| **5** | 1.42 | 0.93 | 0.57 | 2.43 | 1.75 | 1.12 | 1.36 | 1.36 | 1.05 | 1.82 | 0.18 | 1.51 |
| **6** | 0.47 | 0.36 | 0.42 | 1.48 | 1.65 | 2.03 | 1.88 | 1.3 | 1.27 | 1.69 | 0.31 | 0.9 |
| **7** | 1.03 | 0.54 | 0.43 | 1.16 | 0.92 | 1.18 | 0.88 | 1.64 | 0.95 | 2.01 | 0.39 | 1.22 |
| **8** | 1.11 | 0.69 | 0.49 | 1.6 | 0.3 | 1.29 | 1.43 | 1.46 | 0.55 | 2.73 | 0.17 | 0.76 |
| **9** | 0.46 | 0.69 | 0.18 | 1.76 | 0.17 | 2.2 | 1.49 | 0.81 | 0.98 | 2.7 | 0.17 | 1.03 |
| **10** | 0.84 | 0.2 | 0.29 | 1.08 | 0.73 | 1.99 | 1.79 | 1.41 | 0.45 | 1.46 | 0.19 | 1.16 |
| **11** | 1.41 | 0.28 | 0.18 | 0.86 | 0.38 | 1.97 | 1.34 | 1.65 | 1.02 | 1.61 | 0.22 | 0.63 |
| **12** | 1.05 | 0.77 | 0.28 | 1 | 0.74 | 0.78 | 1.13 | 1.78 | 0.55 | 2.23 | 0.21 | 0.44 |
| **13** | 0.35 | 0.55 | 0.34 | 0.52 | 0.76 | 0.64 | 0.23 | 1.53 | 0.29 | 2.44 | 0.17 | 0.74 |
| **14** | 0.78 | 0.48 | 0.17 | 1.29 | 0.35 | 1.16 | 1.8 | 0.92 | 0.8 | 1.94 | 0.29 | 0.9 |
| **15** | 0.6 | 0.57 | 0.32 | 0.85 | 0.85 | 1.37 | 0.91 | 1.21 | 0.35 | 1.64 | 0.2 | 0.61 |
| **16** | 0.44 | 0.17 | 0.17 | 1.09 | 0.62 | 0.43 | 0.68 | 1.06 | 0.51 | 2.06 | 0.22 | 0.86 |
| **17** | 0.76 | 0.66 | 0.17 | 0.24 | 0.51 | 1.11 | 0.79 | 0.83 | 0.68 | 1.7 | 0.17 | 0.55 |
| **18** | 0.59 | 0.52 | 0.19 | 0.54 | 0.58 | 0.87 | 0.61 | 1.05 | 0.25 | 1.31 | 0.31 | 0.55 |
| **19** | 0.8 | 0.2 | 0.17 | 0.67 | 0.17 | 0.64 | 0.85 | 0.86 | 0.67 | 1.02 | 0.24 | 0.52 |
| **20** | 0.89 | 0.68 | 0.17 | 0.45 | 0.7 | 1.92 | 0.6 | 0.69 | 0.87 | 0.96 | 0.24 | 0.61 |

**IPCase Activities – DHC side**

| **Hr** | **Ceramide Substrates** | | | | | | | | | | | |
| --- | --- | --- | --- | --- | --- | --- | --- | --- | --- | --- | --- | --- |
|  | **C14 DHC** | **C16 DHC** | **C18 DHC** | **C18:1 DHC** | **C20 DHC** | **C20:1 DHC** | **C22 DHC** | **C22:1 DHC** | **C24 DHC** | **C24:1 DHC** | **C26 DHC** | **C26:1 DHC** |
| **1** | 2.13 | 0.85 | 1.31 | 1.22 | 0.94 | 1.32 | 1.58 | 1.2 | 1.31 | 1.22 | 1.61 | 1.42 |
| **2** | 1.25 | 0.96 | 1.79 | 1.26 | 1.62 | 0.98 | 1.55 | 0.7 | 1.91 | 1.06 | 1.67 | 1.73 |
| **3** | 0.84 | 1.27 | 0.75 | 1.48 | 1.06 | 0.6 | 1.37 | 1.3 | 1.76 | 0.17 | 1.36 | 1.3 |
| **4** | 0.92 | 0.77 | 1.83 | 1.7 | 0.86 | 0.56 | 1.5 | 0.44 | 1.14 | 0.96 | 1.33 | 2.01 |
| **5** | 1.89 | 0.87 | 0.95 | 1.17 | 1.02 | 0.56 | 1.48 | 0.23 | 1 | 0.17 | 2.05 | 1.64 |
| **6** | 1.41 | 1.13 | 1.73 | 1.75 | 0.77 | 0.9 | 1.35 | 0.55 | 1.17 | 0.69 | 1.52 | 2.03 |
| **7** | 1.93 | 1.73 | 1.3 | 0.78 | 1.08 | 0.63 | 0.81 | 0.33 | 0.92 | 0.41 | 2.22 | 1.24 |
| **8** | 1.47 | 2.21 | 0.89 | 0.63 | 0.44 | 0.54 | 1.61 | 0.51 | 1.58 | 0.17 | 1.75 | 2.08 |
| **9** | 1.64 | 1.04 | 0.77 | 0.75 | 0.87 | 0.21 | 1.35 | 0.32 | 0.86 | 0.69 | 2.25 | 2.73 |
| **10** | 1.52 | 1.99 | 1.38 | 0.98 | 0.63 | 0.17 | 1.63 | 0.6 | 1.16 | 0.58 | 1.54 | 1.62 |
| **11** | 1.69 | 1.54 | 0.7 | 0.83 | 1.17 | 0.54 | 1.57 | 0.25 | 1.25 | 0.8 | 2.12 | 1.82 |
| **12** | 1.99 | 1.68 | 1.26 | 1.08 | 1.85 | 0.68 | 1.35 | 0.83 | 1.16 | 0.18 | 2.24 | 2.33 |
| **13** | 0.91 | 1.04 | 1.16 | 0.88 | 1.18 | 0.42 | 0.82 | 0.52 | 1.67 | 0.19 | 1.26 | 1.95 |
| **14** | 1.3 | 1.77 | 1.63 | 0.41 | 1.46 | 0.63 | 1.84 | 0.17 | 1.72 | 0.9 | 1.36 | 2.41 |
| **15** | 1.52 | 0.67 | 1.8 | 0.88 | 1.48 | 0.51 | 1.52 | 0.53 | 0.9 | 1.22 | 1.6 | 1.25 |
| **16** | 1.77 | 1.74 | 1.08 | 0.75 | 1.39 | 0.24 | 1.14 | 0.26 | 1.8 | 1.03 | 1.79 | 1 |
| **17** | 0.84 | 2.53 | 1.06 | 0.82 | 1.42 | 0.73 | 2.08 | 1.22 | 1.2 | 1.66 | 1.49 | 2.45 |
| **18** | 1.92 | 1.03 | 1.42 | 1.08 | 1.31 | 1.52 | 1.71 | 1.31 | 1.46 | 1.57 | 2.15 | 1.53 |
| **19** | 1.28 | 1.13 | 1.67 | 1.24 | 0.85 | 0.8 | 1.29 | 1.45 | 1.38 | 1.28 | 2.08 | 2.34 |
| **20** | 0.98 | 1.86 | 0.9 | 0.61 | 1.74 | 1.19 | 1.57 | 0.54 | 1.11 | 1.17 | 1.08 | 1.36 |

**IPCase Activities – PHC side**

| **Hrs** | **Ceramide Substrates** | | | | | | | | | | | |
| --- | --- | --- | --- | --- | --- | --- | --- | --- | --- | --- | --- | --- |
|  | **C14 PHC** | **C16 PHC** | **C18 PHC** | **C18:1 PHC** | **C20 PHC** | **C20:1 PHC** | **C22 PHC** | **C22:1 PHC** | **C24 PHC** | **C24:1 PHC** | **C26 PHC** | **C26:1 PHC** |
| **1** | 1.46 | 1.87 | 0.87 | 1.07 | 1.12 | 1.22 | 1.56 | 1.17 | 1.41 | 0.91 | 1.4 | 2 |
| **2** | 1.87 | 1.37 | 1.46 | 0.94 | 0.86 | 1.01 | 1.19 | 1.62 | 1.39 | 1.26 | 1.83 | 1.6 |
| **3** | 1.98 | 1.28 | 1.68 | 1.22 | 0.87 | 1.35 | 1.77 | 0.81 | 1.65 | 1.13 | 2.01 | 1.69 |
| **4** | 2.27 | 1.15 | 1.88 | 0.77 | 1.25 | 0.72 | 1.33 | 0.52 | 1.56 | 0.98 | 2.08 | 2.01 |
| **5** | 1.73 | 2.17 | 1.26 | 0.75 | 1.69 | 0.42 | 1.78 | 0.71 | 2.21 | 0.27 | 1.99 | 2.08 |
| **6** | 1.9 | 1.24 | 1.81 | 0.24 | 1.97 | 0.5 | 1.57 | 1.55 | 1.65 | 0.26 | 2.63 | 2.36 |
| **7** | 1.75 | 1.66 | 2.41 | 0.55 | 1.74 | 0.75 | 1.77 | 1.2 | 1.94 | 0.46 | 2.31 | 2.12 |
| **8** | 1.8 | 2.33 | 1.56 | 1.53 | 2.02 | 0.99 | 0.92 | 0.42 | 1.54 | 0.93 | 1.56 | 1.81 |
| **9** | 1.92 | 1.62 | 1.63 | 1.24 | 1.03 | 0.59 | 2.16 | 0.87 | 1.72 | 0.75 | 1.52 | 2.41 |
| **10** | 2.1 | 1.11 | 1.28 | 1.69 | 1.4 | 0.94 | 1.7 | 1.58 | 2.25 | 0.39 | 1.54 | 2.33 |
| **11** | 1.89 | 1.05 | 1.66 | 0.84 | 1.37 | 1.5 | 1.54 | 1.46 | 2.13 | 0.57 | 1.27 | 2.23 |
| **12** | 1.55 | 1.77 | 1.52 | 0.88 | 1.55 | 1.13 | 0.71 | 1.53 | 2.09 | 0.25 | 1.81 | 1 |
| **13** | 1.02 | 1.9 | 2 | 1.96 | 2.22 | 0.6 | 1.71 | 1.64 | 1.36 | 0.48 | 1.95 | 1.64 |
| **14** | 1.69 | 1.59 | 1.42 | 1.53 | 1.83 | 0.64 | 1.85 | 0.97 | 1.09 | 1.43 | 1.9 | 2.1 |
| **15** | 2.08 | 2.36 | 2.5 | 1.11 | 1.57 | 1.64 | 1.32 | 1.91 | 1.43 | 0.46 | 1.77 | 2.01 |
| **16** | 1.74 | 1.21 | 2.34 | 2.65 | 2.51 | 1.35 | 1.58 | 2.56 | 2.09 | 1.42 | 1.54 | 1.28 |
| **17** | 1.92 | 1.57 | 1.36 | 2.13 | 1.57 | 1.3 | 1.62 | 1.93 | 2 | 1.64 | 1.91 | 1.49 |
| **18** | 1.22 | 2.24 | 2.13 | 2.08 | 2.26 | 1.18 | 1.46 | 2.48 | 2.11 | 1.42 | 2.03 | 1.76 |
| **19** | 1.6 | 1.76 | 1.39 | 2.75 | 1.85 | 0.94 | 1.9 | 2.11 | 2.63 | 0.92 | 2.41 | 1.04 |
| **20** | 1.6 | 1.93 | 1.65 | 1.65 | 2.48 | 1.15 | 1.02 | 1.72 | 1.75 | 1.27 | 1.93 | 2.2 |

**DHC Hydroxylase Activities**

| **Hrs** | **Ceramide Substrates** | | | | | | | | | | | |
| --- | --- | --- | --- | --- | --- | --- | --- | --- | --- | --- | --- | --- |
|  | **C14 PHC** | **C16 PHC** | **C18 PHC** | **C18:1 PHC** | **C20 PHC** | **C20:1 PHC** | **C22 PHC** | **C22:1 PHC** | **C24 PHC** | **C24:1 PHC** | **C26 PHC** | **C26:1 PHC** |
| **1** | 0.39 | 0.87 | 0.57 | 0.9 | 0.33 | 1.39 | 0.74 | 2.35 | 1.99 | 2.13 | 1.98 | 0.91 |
| **2** | 1.44 | 0.32 | 1.04 | 1.42 | 1.47 | 1.31 | 1.19 | 2.99 | 3.96 | 0.76 | 0.17 | 0.54 |
| **3** | 0.32 | 2.83 | 0.47 | 0.43 | 3.99 | 1.3 | 2.73 | 0.46 | 0.85 | 0.85 | 3.61 | 4.64 |
| **4** | 0.91 | 1.21 | 1.01 | 2.52 | 1 | 0.25 | 1.38 | 2.73 | 1.61 | 1.23 | 1.38 | 0.86 |
| **5** | 0.27 | 0.49 | 3.63 | 4.28 | 0.88 | 1.07 | 1.24 | 0.83 | 1.45 | 1.96 | 1.8 | 1.64 |
| **6** | 1.64 | 0.89 | 2.51 | 2.07 | 2.53 | 5 | 2.73 | 0.21 | 0.86 | 0.83 | 4.61 | 3.31 |
| **7** | 0.48 | 0.17 | 0.37 | 0.5 | 0.97 | 3.93 | 0.23 | 3.19 | 1.23 | 2.94 | 5.57 | 0.32 |
| **8** | 0.17 | 0.72 | 1.01 | 0.17 | 0.35 | 0.65 | 0.95 | 0.73 | 0.28 | 0.17 | 5.81 | 0.38 |
| **9** | 1.41 | 0.27 | 2.06 | 0.24 | 0.69 | 4.73 | 4.45 | 1.57 | 0.88 | 1.96 | 4.03 | 0.47 |
| **10** | 1.13 | 1.18 | 1.44 | 0.28 | 1.68 | 3.08 | 0.69 | 2.86 | 0.91 | 0.74 | 0.19 | 1.09 |
| **11** | 1.06 | 0.42 | 1.18 | 1.77 | 4.87 | 2.69 | 1.49 | 5.03 | 0.66 | 0.21 | 1.34 | 0.17 |
| **12** | 2.67 | 1.24 | 0.9 | 2.92 | 0.17 | 3.71 | 0.8 | 1.46 | 0.9 | 2.03 | 0.17 | 1.17 |
| **13** | 0.17 | 2.19 | 2.25 | 1.11 | 1.33 | 0.63 | 0.19 | 0.94 | 0.22 | 1.56 | 4.47 | 1.05 |
| **14** | 0.17 | 0.9 | 0.8 | 1.65 | 4.24 | 6 | 1.56 | 1.01 | 0.61 | 2.29 | 0.81 | 1.48 |
| **15** | 0.17 | 0.17 | 2.5 | 5.47 | 1.14 | 3.32 | 0.73 | 3.92 | 0.53 | 6 | 1.43 | 0.21 |
| **16** | 0.86 | 0.38 | 0.46 | 1.21 | 4.2 | 0.17 | 3.33 | 2.04 | 1.49 | 0.27 | 2.81 | 0.17 |
| **17** | 0.17 | 0.17 | 0.84 | 1.43 | 4.29 | 1.33 | 0.17 | 4.29 | 0.17 | 3.42 | 3.57 | 0.17 |
| **18** | 0.17 | 0.42 | 1.67 | 0.72 | 0.34 | 1.4 | 1.1 | 2.18 | 0.58 | 4.66 | 2.62 | 0.18 |
| **19** | 0.51 | 4.14 | 1.01 | 0.17 | 0.17 | 1.97 | 3.01 | 1.07 | 2.88 | 2.46 | 0.17 | 0.17 |
| **20** | 0.27 | 3.17 | 4.93 | 1.67 | 0.17 | 1.03 | 1.32 | 0.53 | 0.17 | 6 | 0.17 | 0.17 |

**Sphingosine Biosynthesis**

| **Hr** | **Enzyme Names (Substrate Names)** | | | | | | | |
| --- | --- | --- | --- | --- | --- | --- | --- | --- |
|  | **SBK (DHS)** | **SBK (PHS)** | **SB-PPase (DHS)** | **SB-PPase (PHS)** | **Lyase (DHS1p)** | **Lyase (PHS1p)** | **DHS synthesis** | **Hydroxylase** |
| **1** | 1.46 | 1.05 | 1.62 | 0.44 | 1.66 | 1.76 | 1.58 | 7.27 |
| **2** | 0.88 | 0.94 | 1.88 | 0.69 | 0.67 | 1 | 1.09 | 0.34 |
| **3** | 1.16 | 1.11 | 2.42 | 0.61 | 3.96 | 1.52 | 1.73 | 0.56 |
| **4** | 0.81 | 0.76 | 1.61 | 2.46 | 3.84 | 1.27 | 1.21 | 0.74 |
| **5** | 0.74 | 0.95 | 1.89 | 0.8 | 2.19 | 1.56 | 1.66 | 8.05 |
| **6** | 0.44 | 1.52 | 1.38 | 3.21 | 1.51 | 2.07 | 2.02 | 1.23 |
| **7** | 1.07 | 0.96 | 2.29 | 2.96 | 3.92 | 1.09 | 1.38 | 0.41 |
| **8** | 1.17 | 1.01 | 2.83 | 2.57 | 1.91 | 1.45 | 1.25 | 0.52 |
| **9** | 1.26 | 0.83 | 1.59 | 2.24 | 5.24 | 1.77 | 1.95 | 2.69 |
| **10** | 1.15 | 0.7 | 1.58 | 0.78 | 2.88 | 1.34 | 1.5 | 5.71 |
| **11** | 1.5 | 1.08 | 1.72 | 2.73 | 4.69 | 1.39 | 1.77 | 6.18 |
| **12** | 1.19 | 0.79 | 1.35 | 1.8 | 1.25 | 1.41 | 1.25 | 0.79 |
| **13** | 1.7 | 1.02 | 1.65 | 4.19 | 3.26 | 1.94 | 1.92 | 1.5 |
| **14** | 1.39 | 1.04 | 1.2 | 3.76 | 0.75 | 2.44 | 1.52 | 1.08 |
| **15** | 1.73 | 0.71 | 1.71 | 0.81 | 0.75 | 1.72 | 1.5 | 0.05 |
| **16** | 1.64 | 1.29 | 1.84 | 4.53 | 1.51 | 2.34 | 1.67 | 0.05 |
| **17** | 1.97 | 0.78 | 2.02 | 2.62 | 0.61 | 2.15 | 1.41 | 0.05 |
| **18** | 2 | 1.01 | 1.62 | 4.71 | 0.62 | 2.64 | 1.46 | 0.05 |
| **19** | 1.31 | 1.23 | 0.55 | 4.15 | 2.49 | 3.37 | 2.27 | 0.05 |
| **20** | 1.29 | 0.93 | 0.81 | 3.42 | 1.31 | 3.55 | 1.72 | 0.05 |

**Fatty Acid Elongation**

| **Hr** | **Elongase - Saturated Fatty Acyl CoAs as Substrates** | | | | | | | |
| --- | --- | --- | --- | --- | --- | --- | --- | --- |
|  | **C12-C14**  **FA** | **C14-C16**  **FA** | **C16-C18**  **FA** | **C18-C20**  **FA** | **C20-C22**  **FA** | **C22-C24**  **FA** | **C24-C26**  **FA** | **C26->**  **FA** |
| **1** | 1.62 | 1.53 | 1.41 | 0.76 | 0.81 | 2.18 | 1.32 | 2.14 |
| **2** | 1.46 | 1.41 | 1.04 | 1.93 | 1.47 | 1.45 | 1.16 | 1.58 |
| **3** | 1.63 | 1.32 | 1.09 | 0.79 | 1 | 1.78 | 1.05 | 2.54 |
| **4** | 1.4 | 0.83 | 1.36 | 1.08 | 1.17 | 1.54 | 1.1 | 1.24 |
| **5** | 1.55 | 1.71 | 1.69 | 2.18 | 1.26 | 1.5 | 0.51 | 2.65 |
| **6** | 1.24 | 1.4 | 1.74 | 2.22 | 1.19 | 1.57 | 1.1 | 2.17 |
| **7** | 1.56 | 1.32 | 1.08 | 1 | 1.01 | 1.8 | 0.94 | 1.41 |
| **8** | 1.81 | 1.48 | 1.35 | 1.24 | 1.46 | 1.25 | 1.66 | 3.64 |
| **9** | 1.4 | 2.18 | 1.5 | 1.61 | 1.18 | 1.33 | 1.05 | 1.99 |
| **10** | 1.31 | 1.81 | 1.79 | 1.75 | 0.61 | 0.73 | 2.17 | 1.57 |
| **11** | 1.06 | 0.73 | 1.73 | 1.11 | 1.38 | 1.17 | 0.86 | 1.99 |
| **12** | 1.72 | 1.86 | 2.15 | 1.5 | 1.61 | 1.15 | 1.38 | 3.09 |
| **13** | 1.1 | 1.35 | 1.63 | 1.67 | 1.41 | 1.23 | 1.46 | 2.5 |
| **14** | 1.21 | 1.48 | 1.15 | 0.92 | 2.21 | 1.62 | 1.64 | 1.91 |
| **15** | 1.01 | 1.57 | 1.23 | 1.98 | 0.92 | 0.99 | 1.61 | 0.42 |
| **16** | 1.08 | 1.44 | 1.11 | 1.46 | 1.4 | 1.52 | 1.7 | 1.8 |
| **17** | 1.7 | 1.64 | 1.44 | 1.87 | 1.53 | 1.85 | 1.43 | 1.96 |
| **18** | 1.52 | 1.98 | 1.41 | 1.76 | 1.27 | 0.91 | 1.96 | 2.29 |
| **19** | 1.2 | 0.84 | 1.56 | 1.57 | 1.62 | 1.84 | 0.93 | 1.99 |
| **20** | 1.85 | 1.22 | 0.83 | 1.77 | 1.76 | 1.76 | 1.33 | 1.15 |

| **Hr** | **Elongase - Unsaturated Fatty Acyl CoAs as Substrates** | | | | | |
| --- | --- | --- | --- | --- | --- | --- |
|  | **C16:1 -> C18:1 FA** | **C18:1 -> C20:1**  **FA** | **C20:1 -> C22:1**  **FA** | **C22:1 -> C24:1**  **FA** | **C24:1 -> C26:1**  **FA** | **C26:1 ->**  **FA** |
| **1** | 1.49 | 1.59 | 1.44 | 1.98 | 1.64 | 1.36 |
| **2** | 1.65 | 2.02 | 1.24 | 0.89 | 1.5 | 1.07 |
| **3** | 1.83 | 1.77 | 1.72 | 1.15 | 1.03 | 1.72 |
| **4** | 0.93 | 0.94 | 1.62 | 1.09 | 1.71 | 1.61 |
| **5** | 1.55 | 1.3 | 1.56 | 1.02 | 1.35 | 1.8 |
| **6** | 0.73 | 0.72 | 0.83 | 1.54 | 1.09 | 1.05 |
| **7** | 0.72 | 0.48 | 1.15 | 0.98 | 1.62 | 0.27 |
| **8** | 1.45 | 0.92 | 1.13 | 0.99 | 1.72 | 1.61 |
| **9** | 1.61 | 1.39 | 1.1 | 1.4 | 2.17 | 1.39 |
| **10** | 1.54 | 1.92 | 1.38 | 1.3 | 2.24 | 1.18 |
| **11** | 1.6 | 0.89 | 1.34 | 0.81 | 1.71 | 1.05 |
| **12** | 1.3 | 0.68 | 2.01 | 1.18 | 1.17 | 1.11 |
| **13** | 1.74 | 2.08 | 1.72 | 1.57 | 1.3 | 1.24 |
| **14** | 1.86 | 1.44 | 1.27 | 1.43 | 1.97 | 1.44 |
| **15** | 1.77 | 1.42 | 1.76 | 1.2 | 1.67 | 1.94 |
| **16** | 1.15 | 1.27 | 1.66 | 0.87 | 1.57 | 0.46 |
| **17** | 1.02 | 1.26 | 0.9 | 1.08 | 1.44 | 1.33 |
| **18** | 2.04 | 1.34 | 1.92 | 1.15 | 1.52 | 1.65 |
| **19** | 1.64 | 1.51 | 2.13 | 1.55 | 1.58 | 1.77 |
| **20** | 1.46 | 2 | 0.72 | 0.57 | 0.9 | 1.99 |

| **Hr** | **Desaturase - from Saturated to Unsaturated fatty acyl CoAs** | | | | | | |
| --- | --- | --- | --- | --- | --- | --- | --- |
|  | **C14 -> C14:1**  **FA** | **C16 -> C16:1**  **FA** | **C18 -> C18:1**  **FA** | **C20 -> C20:1**  **FA** | **C22 -> C22:1**  **FA** | **C24 -> C24:1**  **FA** | **C26 -> C26:1**  **FA** |
| **1** | 1.82 | 1.3 | 0.74 | 1.02 | 1.27 | 1.03 | 1.13 |
| **2** | 2.75 | 1.62 | 1.55 | 0.57 | 1.39 | 1.73 | 1.8 |
| **3** | 1.59 | 1.23 | 1.85 | 1.51 | 0.85 | 1.95 | 1.94 |
| **4** | 1.2 | 1.25 | 2.24 | 2 | 0.96 | 1.17 | 2.01 |
| **5** | 1 | 1.59 | 1.31 | 1.7 | 1.42 | 2.61 | 1.59 |
| **6** | 0.66 | 0.71 | 1.47 | 1.65 | 0.97 | 0.59 | 0.8 |
| **7** | 1.89 | 2.31 | 1.12 | 2.94 | 1.08 | 1.45 | 1.46 |
| **8** | 0.7 | 1.29 | 1.25 | 1.61 | 1.14 | 2 | 1.73 |
| **9** | 2.04 | 0.77 | 1.88 | 1.51 | 1.49 | 0.86 | 1.69 |
| **10** | 0.95 | 2 | 0.95 | 1.96 | 1.04 | 1.59 | 1.83 |
| **11** | 1.72 | 0.9 | 1 | 2.52 | 2.63 | 1.28 | 1.52 |
| **12** | 1.12 | 2.27 | 1.47 | 1.1 | 0.81 | 1.74 | 0.77 |
| **13** | 1.16 | 0.37 | 0.57 | 1.47 | 1.28 | 2.34 | 1.17 |
| **14** | 0.93 | 2.27 | 1.6 | 1.67 | 1.47 | 1.77 | 0.9 |
| **15** | 1.85 | 0.88 | 1.45 | 0.91 | 1.65 | 1.45 | 1.45 |
| **16** | 1.05 | 1.42 | 1.68 | 1.42 | 1.88 | 1.8 | 1.92 |
| **17** | 2.03 | 0.97 | 0.66 | 0.87 | 1.83 | 1.23 | 1.67 |
| **18** | 1.65 | 1.59 | 1.61 | 2.38 | 2.81 | 1.81 | 1.39 |
| **19** | 0.24 | 1.82 | 1.14 | 1.01 | 1.23 | 1.39 | 2.13 |
| **20** | 2.41 | 1.44 | 1.31 | 2.74 | 1.9 | 0.9 | 1.18 |

**Implementation of the Proposed Methods in Matlab**

**Piecewise Optimization**

**Implementation of the following code will execute the piecewise optimization approach described in the text.**

function Piecewise_Optimization(i)

% Raw_Data_TimeSeries;

load('Stoichiometric_Matrix1')

load('Experiment1');

Data.rate1 = Solution;

rate = Solution;

[DHC_Interpolated, PHC_Interpolated, Sph_Interpolated] = Cubic_Spline_Interpolation;

Data.DHC_Interpolated = DHC_Interpolated;

Data.PHC_Interpolated = PHC_Interpolated;

Data.Sph_Interpolated = Sph_Interpolated;

Fatty_Acyl_CoAs = ones(14,21);

Experiment_X = [DHC_Interpolated;PHC_Interpolated;Sph_Interpolated; Fatty_Acyl_CoAs];

a = 0.8; b = 1.2;

if i == 1

rf = a + (b-a)*rand(42,1);

Original_X = Experiment_X(:,i);

Target_X = Experiment_X(:,i+1) .* rf;

else

rf = a + (b-a)*rand(42,1);

Original_X = Experiment_X(:,i) .* rf;

rf = a + (b-a)*rand(42,1);

Target_X = Experiment_X(:,i+1) .* rf;

end

Data.Original_X = Original_X;

Data.Target_X = Target_X;

%%

a = (1/2);

b = 2;

Rf = a + (b-a)*rand(length(Stoichiometric_Matrix),1);

x0(1:length(Stoichiometric_Matrix),1) = rate.*Rf;

x0_Enzyme = x0;

lb([1:108]) = (1/6)*rate([1:108]);

ub([1:108]) = 6*rate([1:108]);

lb([109:137]) = (1/20)*rate([109:137]);

ub([109:137]) = 20*rate([109:137]);

count = 0;

%%

A = zeros(1,length(Stoichiometric_Matrix));

b = 0;

Data.SM = Stoichiometric_Matrix;

fcn = @(V) Objective_ceramide_TS(V,Data,i);

options = optimset('Display','iter','Algorithm','active-set','MaxFunEvals',300000,'MaxIter',200,'TolFun',1e-12, 'TolX',1e-12,'TolCon',1e-12);

[Vrate, error, flag] = fmincon(fcn, x0, A, b, '', '', lb, ub,'',options);

SSopt = Vrate;

fval = error;

exitflag = flag;

count = count + 1;

%%

while exist(strcat('RatePW',num2str(i),'_',num2str(count),'.mat'),'file')

count=count+1;

end

save(strcat('RatePW',num2str(i),'_',num2str(count)),'SSopt', 'fval','exitflag','Original_X','Target_X','x0_Enzyme')

end

**Note:**  'Stoichiometric_Matrix1' and 'Experiment1' are pre-defined .mat files for the stoichiometric matrix and the steady-state flux estimates.

‘Cubic_Spline_Interpolation’ is a self-defined function given below.

function [DHC_Interpolated, PHC_Interpolated, Sph_Interpolated] = Cubic_Spline_Interpolation

load('Data_TimeSeries');

WT_DHC = DataTS.WT_DHC;

WT_PHC = DataTS.WT_PHC;

WT_Sph = DataTS.WT_Sph;

WT_DHC = [ones(1,12);WT_DHC];

WT_PHC = [ones(1,12);WT_PHC];

WT_Sph = [ones(1,4);WT_Sph];

load('Data_RawTimeSeries');

DHC_WT_3hours = DataRTS.DHC_WT_3hours;

PHC_WT_3hours = DataRTS.PHC_WT_3hours;

Sph_WT_3hours = DataRTS.Sph_WT_3hours;

DHC_WT_20hours = DataRTS.DHC_WT_20hours;

PHC_WT_20hours = DataRTS.PHC_WT_20hours;

Sph_WT_20hours = DataRTS.Sph_WT_20hours;

DHC_isc_3hours = DataRTS.DHC_isc_3hours;

PHC_isc_3hours = DataRTS.PHC_isc_3hours;

Sph_isc_3hours = DataRTS.Sph_isc_3hours;

DHC_isc_20hours = DataRTS.DHC_isc_20hours;

PHC_isc_20hours = DataRTS.PHC_isc_20hours;

Sph_isc_20hours = DataRTS.Sph_isc_20hours;

Tdata = [0 3 20];

Tfit = [0:1:20];

fs = 14;

%% Fit DHC

figure(1)

p_DHC = [1 1 1 1 1 1 1 1 1 1 1 1];

yl_DHC = [4 4 4 4 4 4 4 4 4 4 4 4];

for i = 1:12

cf_DHC(i) = csaps(Tdata,WT_DHC(:,i),p_DHC(i));

DHC_Interpolated(i,:) = fnval(cf_DHC(i),Tfit);

subplot(2,6,i)

plot(Tfit,DHC_Interpolated(i,:),'b',Tdata,WT_DHC(:,i),'ro','LineWidth',3,'MarkerSize',8)

hold on

plot(Tdata(2),DHC_WT_3hours(:,i),'bo',Tdata(3),DHC_WT_20hours(:,i),'bo','MarkerSize',8)

hold on

plot(Tdata(2),DHC_WT_3hours(:,i),'bo',Tdata(3),DHC_WT_20hours(:,i),'bo','MarkerSize',4)

hold on

plot(Tfit,DHC_Interpolated(i,:)*1.2,'b',Tfit,DHC_Interpolated(i,:)*0.8,'b','LineWidth',1.5)

ylim([0,yl_DHC(i)]);

xlim([0,21]);

set(gca,'fontsize',fs);

axh = gca;

set(axh,'XGrid','on','XTick',[0,3,20]);

hold on

end

%% Fit PHC

figure(2)

p_PHC = [1 1 1 1 1 1 1 1 1 0.1 1 1];

yl_PHC = [5 5 15 5 10 5 5 5 5 5 15 5];

for i = 1:12

cf_PHC(i) = csaps(Tdata,WT_PHC(:,i),p_PHC(i));

PHC_Interpolated(i,:) = fnval(cf_PHC(i),Tfit);

subplot(2,6,i)

plot(Tfit,PHC_Interpolated(i,:),'b',Tdata,WT_PHC(:,i),'ro','LineWidth',3,'MarkerSize',8)

hold on

plot(Tdata(2),PHC_WT_3hours(:,i),'bo',Tdata(3),PHC_WT_20hours(:,i),'bo','MarkerSize',8)

hold on

plot(Tdata(2),PHC_WT_3hours(:,i),'bo',Tdata(3),PHC_WT_20hours(:,i),'bo','MarkerSize',4)

hold on

plot(Tfit,PHC_Interpolated(i,:)*1.2,'b',Tfit,PHC_Interpolated(i,:)*0.8,'b','LineWidth',1.5)

ylim([0,yl_PHC(i)]);

xlim([0,21]);

set(gca,'fontsize',fs);

axh = gca;

set(axh,'XGrid','on','XTick',[0,3,20]);

hold on

end

%% Fit Sph

figure(3)

p_Sph = [0.3 1 0.2 1];

yl_Sph = [6 6 6 6];

for i = 1:4

cf_Sph(i) = csaps(Tdata,WT_Sph(:,i),p_Sph(i));

Sph_Interpolated(i,:) = fnval(cf_Sph(i),Tfit);

subplot(1,4,i)

plot(Tfit,Sph_Interpolated(i,:),'b',Tdata,WT_Sph(:,i),'ro','LineWidth',3,'MarkerSize',8)

hold on

plot(Tdata(2),Sph_WT_3hours(:,i),'bo',Tdata(3),Sph_WT_20hours(:,i),'bo','MarkerSize',8)

hold on

plot(Tdata(2),Sph_WT_3hours(:,i),'bo',Tdata(3),Sph_WT_20hours(:,i),'bo','MarkerSize',4)

hold on

plot(Tfit,Sph_Interpolated(i,:)*1.2,'b',Tfit,Sph_Interpolated(i,:)*0.8,'b','LineWidth',1.5)

ylim([0,yl_Sph(i)]);

xlim([0,21]);

set(gca,'fontsize',fs);

axh = gca;

set(axh,'XGrid','on','XTick',[0,3,20]);

hold on

end

end

**Note:** 'Data_TimeSeries' and 'Data_RawTimeSeries' are pre-defined .mat files containing the experimental data. Please use the following code to obtain these files.

function Raw_Data_TimeSeries

%% PHC WT + HU 3 hours

PHC_WT_3hours(1,:) = [1.25 2.22 2.35 0.66 1.37 0.94 0.88 1.15 1.23 0.4 2.5 0.75];

PHC_WT_3hours(2,:) = [1.69 1.43 1.24 0.65 1.18 0.52 1 0.39 1.91 0.3 6.24 2.54];

Mean_PHC_WT_3hours = mean(PHC_WT_3hours);

DataRTS.PHC_WT_3hours = PHC_WT_3hours;

%% PHC isc + HU 3 hours

PHC_isc_3hours(1,:) = [0.64 1.02 0.95 0.72 1.08 0.46 1.11 0.66 2 0.39 2.68 1.34];

PHC_isc_3hours(2,:) = [ 3.6 0.72 2.17 1.82 1.71 1.18 2.04 0.99 1.52 1.22 1.67 1.37];

Mean_PHC_isc_3hours = mean(PHC_isc_3hours);

DataRTS.PHC_isc_3hours = PHC_isc_3hours;

%% PHC WT + HU 20 hours

PHC_WT_20hours(1,:) = [2.9 4.4 9.52 4 8.77 2.2 2.8 4.6 2.6 0.28 13 2];

PHC_WT_20hours(2,:) = [1.9 3 11.26 4.46 1.99 0.92 1.67 0.92 2.7 2.47 5.55 4];

Mean_PHC_WT_20hours = mean(PHC_WT_20hours);

DataRTS.PHC_WT_20hours = PHC_WT_20hours;

%% PHC isc + HU 20 hours

PHC_isc_20hours(1,:) = [0.51 0.88 0.64 0.22 0.39 0.22 0.42 0.45 1.8 0.14 0.89 0.32];

PHC_isc_20hours(2,:) = [3.7 3.4 2.34 0.65 3.01 1.88 1.89 1.27 4.25 1.03 0.29 1.78];

Mean_PHC_isc_20hours = mean(PHC_isc_20hours);

DataRTS.PHC_isc_20hours = PHC_isc_20hours;

DataTS.WT_PHC = [Mean_PHC_WT_3hours; Mean_PHC_WT_20hours];

DataTS.isc_PHC = [Mean_PHC_isc_3hours; Mean_PHC_isc_20hours];

%%=========================================================================

%% DHC WT + HU 3 hours

DHC_WT_3hours(1,:) = [0.88 0.66 0.41 0.36 0.36 0.22 0.69 0.43 0.45 0.36 0.87 0.89];

DHC_WT_3hours(2,:) = [1.46 1.99 1.56 1.24 1.04 1.12 1.15 0.71 1.46 0.94 1.99 1.9];

Mean_DHC_WT_3hours = mean(DHC_WT_3hours);

DataRTS.DHC_WT_3hours = DHC_WT_3hours;

%% DHC isc + HU 3 hours

DHC_isc_3hours(1,:) = [0.67 0.63 0.69 0.44 0.71 0.52 0.68 0.53 1.35 0.73 2.75 2.42];

DHC_isc_3hours(2,:) = [1.19 1.75 1.58 1.43 1.28 1.14 0.79 0.79 1.14 0.39 1.49 0.97];

Mean_DHC_isc_3hours = mean(DHC_isc_3hours);

DataRTS.DHC_isc_3hours = DHC_isc_3hours;

%% DHC WT + HU 20 hours

DHC_WT_20hours(1,:) = [1.77 1.74 0.93 0.68 1.34 0.92 1.54 1.17 1.34 0.55 3.6 2];

DHC_WT_20hours(2,:) = [0.79 1.88 1.92 0.96 0.93 0.68 0.95 0.51 1.92 0.64 2.2 2.68];

Mean_DHC_WT_20hours = mean(DHC_WT_20hours);

DataRTS.DHC_WT_20hours = DHC_WT_20hours;

%% DHC isc + HU 20 hours

DHC_isc_20hours(1,:) = [0.12 0.1 0.034 0.02 0.02 0.018 0.11 0.066 0.67 1.49 0.49 1.12];

DHC_isc_20hours(2,:) = [3.4 1.78 2.05 1 1.27 0.76 2.04 0.96 2.99 2.7 2.26 1.63];

Mean_DHC_isc_20hours = mean(DHC_isc_20hours);

DataRTS.DHC_isc_20hours = DHC_isc_20hours;

DataTS.WT_DHC = [Mean_DHC_WT_3hours; Mean_DHC_WT_20hours];

DataTS.isc_DHC = [Mean_DHC_isc_3hours; Mean_DHC_isc_20hours];

%%=========================================================================

%% Sph WT + HU 3 hours

Sph_WT_3hours(1,:) = [0.59 1.23 0.11 0.27];

Sph_WT_3hours(2,:) = [1.02 1.9 0.63 1.88];

Mean_Sph_WT_3hours = mean(Sph_WT_3hours);

DataRTS.Sph_WT_3hours = Sph_WT_3hours;

%% Sph isc + HU 3 hours

Sph_isc_3hours(1,:) = [0.99 0.88 0.31 0.24];

Sph_isc_3hours(2,:) = [0.76 1.43 1.37 1.84];

Mean_Sph_isc_3hours = mean(Sph_isc_3hours);

DataRTS.Sph_isc_3hours = Sph_isc_3hours;

%% Sph WT + HU 20 hours

Sph_WT_20hours(1,:) = [1.59 1.47 0.6 0.37];

Sph_WT_20hours(2,:) = [3.72 1 5.02 0.5];

Mean_Sph_WT_20hours = mean(Sph_WT_20hours);

DataRTS.Sph_WT_20hours = Sph_WT_20hours;

%% Sph isc + HU 20 hours

Sph_isc_20hours(1,:) = [0.55 0.27 0.16 0.016];

Sph_isc_20hours(2,:) = [0.94 2.22 4.1 0.9];

Mean_Sph_isc_20hours = mean(Sph_isc_20hours);

DataRTS.Sph_isc_20hours = Sph_isc_20hours;

DataTS.WT_Sph = [Mean_Sph_WT_3hours; Mean_Sph_WT_20hours];

DataTS.isc_Sph = [Mean_Sph_isc_3hours; Mean_Sph_isc_20hours];

%%

save('Data_TimeSeries','DataTS');

save('Data_RawTimeSeries','DataRTS');

%%

load('Data_TimeSeries');

Data.WT_PHC = DataTS.WT_PHC;

Data.WT_DHC = DataTS.WT_DHC;

Data.WT_Sph = DataTS.WT_Sph;

Data.isc_PHC = DataTS.isc_PHC;

Data.isc_DHC = DataTS.isc_DHC;

Data.isc_Sph = DataTS.isc_Sph;

load('Data_RawTimeSeries');

Data.DHC_WT_3hours = DataRTS.DHC_WT_3hours;

Data.PHC_WT_3hours = DataRTS.PHC_WT_3hours;

Data.Sph_WT_3hours = DataRTS.Sph_WT_3hours;

Data.DHC_WT_20hours = DataRTS.DHC_WT_20hours;

Data.PHC_WT_20hours = DataRTS.PHC_WT_20hours;

Data.Sph_WT_20hours = DataRTS.Sph_WT_20hours;

Data.DHC_isc_3hours = DataRTS.DHC_isc_3hours;

Data.PHC_isc_3hours = DataRTS.PHC_isc_3hours;

Data.Sph_isc_3hours = DataRTS.Sph_isc_3hours;

Data.DHC_isc_20hours = DataRTS.DHC_isc_20hours;

Data.PHC_isc_20hours = DataRTS.PHC_isc_20hours;

Data.Sph_isc_20hours = DataRTS.Sph_isc_20hours;

end

**Generation of Plots**

**The following code generates the plots of enzyme activities in the text.**

function Main_Function_TS

load(strcat('Stoichiometric_Matrix1'));

load('Experiment1');

Data.rate1 = Solution;

clear Solution;

load('Data_TimeSeries');

Data.WT_PHC = DataTS.WT_PHC;

Data.WT_DHC = DataTS.WT_DHC;

Data.WT_Sph = DataTS.WT_Sph;

Data.isc_PHC = DataTS.isc_PHC;

Data.isc_DHC = DataTS.isc_DHC;

Data.isc_Sph = DataTS.isc_Sph;

load('Data_RawTimeSeries');

Data.DHC_WT_3hours = DataRTS.DHC_WT_3hours;

Data.PHC_WT_3hours = DataRTS.PHC_WT_3hours;

Data.Sph_WT_3hours = DataRTS.Sph_WT_3hours;

Data.DHC_WT_20hours = DataRTS.DHC_WT_20hours;

Data.PHC_WT_20hours = DataRTS.PHC_WT_20hours;

Data.Sph_WT_20hours = DataRTS.Sph_WT_20hours;

Data.DHC_isc_3hours = DataRTS.DHC_isc_3hours;

Data.PHC_isc_3hours = DataRTS.PHC_isc_3hours;

Data.Sph_isc_3hours = DataRTS.Sph_isc_3hours;

Data.DHC_isc_20hours = DataRTS.DHC_isc_20hours;

Data.PHC_isc_20hours = DataRTS.PHC_isc_20hours;

Data.Sph_isc_20hours = DataRTS.Sph_isc_20hours;

%%

[DHC_Interpolated, PHC_Interpolated, Sph_Interpolated] = Cubic_Spline_Interpolation;

Data.DHC_Interpolated = DHC_Interpolated;

Data.PHC_Interpolated = PHC_Interpolated;

Data.Sph_Interpolated = Sph_Interpolated;

count = 0;

error_tolerance(1:5) = [0.08 0.15 0.3 0.4 0.15];

error_tolerance(6:10) = [0.5 0.4 0.5 0.5 0.5];

error_tolerance(11:15) = [0.4 0.4 0.4 0.3 0.5];

error_tolerance(16:20) = [0.4 0.4 0.5 0.5 0.8];

for i = 1:20

for j = 1:100

if exist(strcat('RatePW',num2str(i),'_',num2str(j),'.mat'),'file')

load(strcat('RatePW',num2str(i),'_',num2str(j),'.mat'));

count = count + 1;

FVAL(i,count)= fval;

if fval < error_tolerance(i)

Data.SSopt = SSopt;

t0 = (i-1)*100;

dt = 1;

tf = i*100;

tspan = [t0:dt:tf];

x0 = Original_X;

Data.Original_X = Original_X;

Data.Target_X = Target_X;

index = i;

rate = SSopt;

Data.V = rate;

Data.SM = Stoichiometric_Matrix;

options = odeset('Events',@Ceramide_events,'AbsTol',1e-12,'RelTol',1e-12);

[T XX TE YE IE]=ode15s(@(t,Xd)Ceramide_Model_TS(t,Xd,Data),tspan,x0,options);

% plot_ode_TS(T,XX,Data,index,tspan);

plot_enzyme_TS(Data,i);

else

end

else

end

end

count = 0;

end

plot_enzyme_mean;

Regression_Test;

end

**Note:** ‘Plot_enzyme_TS’, ‘plot_enzyme_mean’ and ‘Regression_Test’ are three self-defined functions that help plot the figures (see below).

function plot_enzyme_TS(Data,i)

Id1 = [1,6,11,16,21,26,31,36,41,46,51,56]; % CerS DHC

Id2 = [61,65,69,73,77,81,85,89,93,97,101,105]; % CerS PHC

Id3 = [3,8,13,18,23,28,33,38,43,48,53,58]; % Dihydroceramidase

Id4 = [63,67,71,75,79,83,87,91,95,99,103,107]; % Phytoceramidase

Id5 = [5,10,15,20,25,30,35,40,45,50,55,60]; % IPC Synthase DHC

Id6 = [64,68,72,76,80,84,88,92,96,100,104,108]; % IPC Synthase PHC

Id7 = [2,7,12,17,22,27,32,37,42,47,52,57]; % IPCase DHC

Id8 = [62,66,70,74,78,82,86,90,94,98,102,106]; % IPCase PHC

Id9 = [4,9,14,19,24,29,34,39,44,49,54,59]; % Hydroxylase

Id10 = [111,114,110,113,115,116,109,112]; % DHS & PHS

Id11 = [117:124,125:130,136,137,131:135]; % Fatty acid elongation

SS = Data.rate1;

SSopt = Data.SSopt;

Fold_Change_Enzyme = SSopt(1:137)./SS;

ub = 2;

lb = -2;

fs = 18;

ms = 6;

ylimit = [-3,3];

%% Ceramide Synthase

figure(7)

for j = 1:length(Id1)

subplot(2,6,j);

plot(i,log2(Fold_Change_Enzyme([Id1(j)])),'*','color',[190 190 190]/255,'MarkerSize',ms)

xlim([0,21]);

ylim(ylimit);

% xlabel('Time')

% ylabel('Rate constants')

set(gca,'fontsize',fs,'XTickLabel',[],'YTickLabel',[],'XGrid','on','XTick',[0:5:20]);

% axh = gca;

% set(axh,'XGrid','on','XTick',[0:5:20]);

hold on

end

figure(8)

for j = 1:length(Id2)

subplot(2,6,j);

plot(i,log2(Fold_Change_Enzyme([Id2(j)])),'*','color',[190 190 190]/255,'MarkerSize',ms)

xlim([0,21]);

ylim(ylimit);

% xlabel('Time')

% ylabel('Rate constants')

set(gca,'fontsize',fs,'XTickLabel',{[]},'YTickLabel',{[]});

axh = gca;

set(axh,'XGrid','on','XTick',[0:5:20]);

hold on

plot([0,21],ub*ones(1,2),'color',[190 190 190]/255);

plot([0,21],lb*ones(1,2),'color',[190 190 190]/255);

hold on

end

%% Ceramidase

figure(9)

for j = 1:length(Id3)

subplot(2,6,j);

plot(i,log2(Fold_Change_Enzyme([Id3(j)])),'*','color',[190 190 190]/255,'MarkerSize',ms)

xlim([0,21]);

ylim(ylimit);

% xlabel('Time')

% ylabel('Rate constants')

set(gca,'fontsize',fs,'XTickLabel',{[]},'YTickLabel',{[]});

axh = gca;

set(axh,'XGrid','on','XTick',[0:5:20]);

hold on

plot([0,21],ub*ones(1,2),'color',[190 190 190]/255);

plot([0,21],lb*ones(1,2),'color',[190 190 190]/255);

hold on

end

figure(10)

for j = 1:length(Id4)

subplot(2,6,j);

plot(i,log2(Fold_Change_Enzyme([Id4(j)])),'*','color',[190 190 190]/255,'MarkerSize',ms)

xlim([0,21]);

ylim(ylimit);

% xlabel('Time')

% ylabel('Rate constants')

set(gca,'fontsize',fs,'XTickLabel',{[]},'YTickLabel',{[]});

axh = gca;

set(axh,'XGrid','on','XTick',[0:5:20]);

hold on

plot([0,21],ub*ones(1,2),'color',[190 190 190]/255);

plot([0,21],lb*ones(1,2),'color',[190 190 190]/255);

hold on

end

%% IPC Synthase

figure(11)

for j = 1:length(Id5)

subplot(2,6,j);

plot(i,log2(Fold_Change_Enzyme([Id5(j)])),'*','color',[190 190 190]/255,'MarkerSize',ms)

xlim([0,21]);

ylim(ylimit);

% xlabel('Time')

% ylabel('Rate constants')

set(gca,'fontsize',fs,'XTickLabel',{[]},'YTickLabel',{[]});

axh = gca;

set(axh,'XGrid','on','XTick',[0:5:20]);

hold on

plot([0,21],ub*ones(1,2),'color',[190 190 190]/255);

plot([0,21],lb*ones(1,2),'color',[190 190 190]/255);

hold on

end

figure(12)

for j = 1:length(Id6)

subplot(2,6,j);

plot(i,log2(Fold_Change_Enzyme([Id6(j)])),'*','color',[190 190 190]/255,'MarkerSize',ms)

xlim([0,21]);

ylim(ylimit);

% xlabel('Time')

% ylabel('Rate constants')

set(gca,'fontsize',fs,'XTickLabel',{[]},'YTickLabel',{[]});

axh = gca;

set(axh,'XGrid','on','XTick',[0:5:20]);

hold on

plot([0,21],ub*ones(1,2),'color',[190 190 190]/255);

plot([0,21],lb*ones(1,2),'color',[190 190 190]/255);

hold on

end

%% IPCase

figure(13)

for j = 1:length(Id7)

subplot(2,6,j);

plot(i,log2(Fold_Change_Enzyme([Id7(j)])),'*','color',[190 190 190]/255,'MarkerSize',ms)

xlim([0,21]);

ylim(ylimit);

% xlabel('Time')

% ylabel('Rate constants')

set(gca,'fontsize',fs,'XTickLabel',{[]},'YTickLabel',{[]});

axh = gca;

set(axh,'XGrid','on','XTick',[0:5:20]);

hold on

plot([0,21],ub*ones(1,2),'color',[190 190 190]/255);

plot([0,21],lb*ones(1,2),'color',[190 190 190]/255);

hold on

end

figure(14)

for j = 1:length(Id8)

subplot(2,6,j);

plot(i,log2(Fold_Change_Enzyme([Id8(j)])),'*','color',[190 190 190]/255,'MarkerSize',ms)

xlim([0,21]);

ylim(ylimit);

% xlabel('Time')

% ylabel('Rate constants')

set(gca,'fontsize',fs,'XTickLabel',{[]},'YTickLabel',{[]});

axh = gca;

set(axh,'XGrid','on','XTick',[0:5:20]);

hold on

plot([0,21],ub*ones(1,2),'color',[190 190 190]/255);

plot([0,21],lb*ones(1,2),'color',[190 190 190]/255);

hold on

end

%% Hydroxylase

figure(15)

for j = 1:length(Id9)

subplot(2,6,j);

plot(i,log2(Fold_Change_Enzyme([Id9(j)])),'*','color',[190 190 190]/255,'MarkerSize',ms)

xlim([0,21]);

ylim(ylimit);

% xlabel('Time')

% ylabel('Rate constants')

set(gca,'fontsize',fs,'XTickLabel',{[]},'YTickLabel',{[]});

axh = gca;

set(axh,'XGrid','on','XTick',[0:5:20]);

hold on

plot([0,21],ub*ones(1,2),'color',[190 190 190]/255);

plot([0,21],lb*ones(1,2),'color',[190 190 190]/255);

hold on

end

%% Sphingosine

figure(16)

for j = 1:length(Id10)

subplot(2,4,j);

plot(i,log2(Fold_Change_Enzyme([Id10(j)])),'*','color',[190 190 190]/255,'MarkerSize',ms)

xlim([0,21]);

ylim(ylimit);

% xlabel('Time')

% ylabel('Rate constants')

set(gca,'fontsize',fs,'XTickLabel',{[]},'YTickLabel',{[]});

axh = gca;

set(axh,'XGrid','on','XTick',[0:5:20]);

hold on

plot([0,21],ub*ones(1,2),'color',[190 190 190]/255);

plot([0,21],lb*ones(1,2),'color',[190 190 190]/255);

hold on

end

%% Fatty acid elongation

figure(17)

for j = 1:8

subplot(3,8,j);

plot(i,log2(Fold_Change_Enzyme([Id11(j)])),'*','color',[190 190 190]/255,'MarkerSize',ms)

xlim([0,21]);

ylim(ylimit);

% xlabel('Time')

% ylabel('Rate constants')

set(gca,'fontsize',fs,'XTickLabel',{[]},'YTickLabel',{[]});

axh = gca;

set(axh,'XGrid','on','XTick',[0:5:20]);

hold on

plot([0,21],ub*ones(1,2),'color',[190 190 190]/255);

plot([0,21],lb*ones(1,2),'color',[190 190 190]/255);

hold on

end

%%=========================================================================

for j = 1:6

subplot(3,8,8+j);

plot(i,log2(Fold_Change_Enzyme([Id11(8+j)])),'*','color',[190 190 190]/255,'MarkerSize',ms)

xlim([0,21]);

ylim(ylimit);

% xlabel('Time')

% ylabel('Rate constants')

set(gca,'fontsize',fs,'XTickLabel',{[]},'YTickLabel',{[]});

axh = gca;

set(axh,'XGrid','on','XTick',[0:5:20]);

hold on

plot([0,21],ub*ones(1,2),'color',[190 190 190]/255);

plot([0,21],lb*ones(1,2),'color',[190 190 190]/255);

hold on

end

%%=========================================================================

for j = 1:7

subplot(3,8,16+j);

plot(i,log2(Fold_Change_Enzyme([Id11(14+j)])),'*','color',[190 190 190]/255,'MarkerSize',ms)

xlim([0,21]);

ylim(ylimit);

% xlabel('Time')

% ylabel('Rate constants')

set(gca,'fontsize',fs,'XTickLabel',{[]},'YTickLabel',{[]});

axh = gca;

set(axh,'XGrid','on','XTick',[0:5:20]);

hold on

plot([0,21],ub*ones(1,2),'color',[190 190 190]/255);

plot([0,21],lb*ones(1,2),'color',[190 190 190]/255);

hold on

end

end

function plot_enzyme_mean

load('Experiment1');

Data.rate1 = Solution;

clear Solution;

count = zeros(1,20);

error_tolerance(1:5) = [0.08 0.15 0.3 0.4 0.15];

error_tolerance(6:10) = [0.5 0.4 0.5 0.5 0.5];

error_tolerance(11:15) = [0.4 0.4 0.4 0.3 0.5];

error_tolerance(16:20) = [0.4 0.4 0.5 0.5 0.8];

for i = 1:20

for j = 1:100

if exist(strcat('RatePW',num2str(i),'_',num2str(j),'.mat'),'file')

load(strcat('RatePW',num2str(i),'_',num2str(j),'.mat'));

if fval < error_tolerance(i)

count(1,i) = count(1,i) + 1;

else

end

else

end

end

end

%%

mean_SSopt = zeros(137,20);

for i = 1:20

for j = 1:100

if exist(strcat('RatePW',num2str(i),'_',num2str(j),'.mat'),'file')

load(strcat('RatePW',num2str(i),'_',num2str(j),'.mat'));

if fval < error_tolerance(i)

mean_SSopt(:,i) = mean_SSopt(:,i) + SSopt*count(1,i)^-1;

else

end

else

end

end

end

Id1 = [1,6,11,16,21,26,31,36,41,46,51,56]; % CerS DHC

Id2 = [61,65,69,73,77,81,85,89,93,97,101,105]; % CerS PHC

Id3 = [3,8,13,18,23,28,33,38,43,48,53,58]; % Dihydroceramidase

Id4 = [63,67,71,75,79,83,87,91,95,99,103,107]; % Phytoceramidase

Id5 = [5,10,15,20,25,30,35,40,45,50,55,60]; % IPC Synthase DHC

Id6 = [64,68,72,76,80,84,88,92,96,100,104,108]; % IPC Synthase PHC

Id7 = [2,7,12,17,22,27,32,37,42,47,52,57]; % IPCase DHC

Id8 = [62,66,70,74,78,82,86,90,94,98,102,106]; % IPCase PHC

Id9 = [4,9,14,19,24,29,34,39,44,49,54,59]; % Hydroxylase

Id10 = [111,114,110,113,115,116,109,112]; % DHS & PHS

Id11 = [117:124,125:130,136,137,131:135]; % Fatty acid elongation

SS = Data.rate1;

Mean_Fold_Change_Enzmye = mean_SSopt./ndgrid(SS,[1:20]);

t = [1:20];

LW = 8;

fs = 18;

ylimit = [-3,3];

%% Ceramide Synthase

figure(7)

for j = 1:length(Id1)

subplot(2,6,j);

plot(t,log2(Mean_Fold_Change_Enzmye(Id1(j),:)),'-b','LineWidth',LW)

xlim([0,21]);

ylim(ylimit);

% xlabel('Time')

% ylabel('Rate constants')

set(gca,'fontsize',fs,'XTickLabel',{[]},'YTickLabel',{[]});

axh = gca;

set(axh,'XGrid','on','XTick',[0:5:20]);

hold on

end

figure(8)

for j = 1:length(Id2)

subplot(2,6,j);

plot(t,log2(Mean_Fold_Change_Enzmye(Id2(j),:)),'-b','LineWidth',LW)

xlim([0,21]);

ylim(ylimit);

% xlabel('Time')

% ylabel('Rate constants')

set(gca,'fontsize',fs,'XTickLabel',{[]},'YTickLabel',{[]});

axh = gca;

set(axh,'XGrid','on','XTick',[0:5:20]);

hold on

end

%% Ceramidase

figure(9)

for j = 1:length(Id3)

subplot(2,6,j);

plot(t,log2(Mean_Fold_Change_Enzmye(Id3(j),:)),'-b','LineWidth',LW)

xlim([0,21]);

ylim(ylimit);

% xlabel('Time')

% ylabel('Rate constants')

set(gca,'fontsize',fs,'XTickLabel',{[]},'YTickLabel',{[]});

axh = gca;

set(axh,'XGrid','on','XTick',[0:5:20]);

hold on

end

figure(10)

for j = 1:length(Id4)

subplot(2,6,j);

plot(t,log2(Mean_Fold_Change_Enzmye(Id4(j),:)),'-b','LineWidth',LW)

xlim([0,21]);

ylim(ylimit);

% xlabel('Time')

% ylabel('Rate constants')

set(gca,'fontsize',fs,'XTickLabel',{[]},'YTickLabel',{[]});

axh = gca;

set(axh,'XGrid','on','XTick',[0:5:20]);

hold on

end

%% IPC Synthase

figure(11)

for j = 1:length(Id5)

subplot(2,6,j);

plot(t,log2(Mean_Fold_Change_Enzmye(Id5(j),:)),'-b','LineWidth',LW)

xlim([0,21]);

ylim(ylimit);

% xlabel('Time')

% ylabel('Rate constants')

set(gca,'fontsize',fs,'XTickLabel',{[]},'YTickLabel',{[]});

axh = gca;

set(axh,'XGrid','on','XTick',[0:5:20]);

hold on

end

figure(12)

for j = 1:length(Id6)

subplot(2,6,j);

plot(t,log2(Mean_Fold_Change_Enzmye(Id6(j),:)),'-b','LineWidth',LW)

xlim([0,21]);

ylim(ylimit);

% xlabel('Time')

% ylabel('Rate constants')

set(gca,'fontsize',fs,'XTickLabel',{[]},'YTickLabel',{[]});

axh = gca;

set(axh,'XGrid','on','XTick',[0:5:20]);

hold on

end

%% IPCase

figure(13)

for j = 1:length(Id7)

subplot(2,6,j);

plot(t,log2(Mean_Fold_Change_Enzmye(Id7(j),:)),'-b','LineWidth',LW)

xlim([0,21]);

ylim(ylimit);

% xlabel('Time')

% ylabel('Rate constants')

set(gca,'fontsize',fs,'XTickLabel',{[]},'YTickLabel',{[]});

axh = gca;

set(axh,'XGrid','on','XTick',[0:5:20]);

hold on

end

figure(14)

for j = 1:length(Id8)

subplot(2,6,j);

plot(t,log2(Mean_Fold_Change_Enzmye(Id8(j),:)),'-b','LineWidth',LW)

xlim([0,21]);

ylim(ylimit);

% xlabel('Time')

% ylabel('Rate constants')

set(gca,'fontsize',fs,'XTickLabel',{[]},'YTickLabel',{[]});

axh = gca;

set(axh,'XGrid','on','XTick',[0:5:20]);

hold on

end

%% Hydroxylase

figure(15)

for j = 1:length(Id9)

subplot(2,6,j);

plot(t,log2(Mean_Fold_Change_Enzmye(Id9(j),:)),'-b','LineWidth',LW)

xlim([0,21]);

ylim(ylimit);

% xlabel('Time')

% ylabel('Rate constants')

set(gca,'fontsize',fs,'XTickLabel',{[]},'YTickLabel',{[]});

axh = gca;

set(axh,'XGrid','on','XTick',[0:5:20]);

hold on

end

%% Sphingosine

figure(16)

for j = 1:length(Id10)

subplot(2,4,j);

plot(t,log2(Mean_Fold_Change_Enzmye(Id10(j),:)),'-b','LineWidth',LW)

xlim([0,21]);

ylim(ylimit);

% xlabel('Time')

% ylabel('Rate constants')

set(gca,'fontsize',fs,'XTickLabel',{[]},'YTickLabel',{[]});

axh = gca;

set(axh,'XGrid','on','XTick',[0:5:20]);

hold on

end

%% Fatty acyl CoAs concentration

figure(17)

for j = 1:8

subplot(3,8,j);

plot(t,log2(Mean_Fold_Change_Enzmye(Id11(j),:)),'-b','LineWidth',LW)

xlim([0,21]);

ylim(ylimit);

set(gca,'fontsize',fs,'XTickLabel',{[]},'YTickLabel',{[]});

axh = gca;

set(axh,'XGrid','on','XTick',[0:5:20]);

hold on

end

%%=========================================================================

for j = 1:6

subplot(3,8,8+j);

plot(t,log2(Mean_Fold_Change_Enzmye(Id11(8+j),:)),'-b','LineWidth',LW)

xlim([0,21]);

ylim(ylimit);

set(gca,'fontsize',fs,'XTickLabel',{[]},'YTickLabel',{[]});

axh = gca;

set(axh,'XGrid','on','XTick',[0:5:20]);

hold on

end

%%=========================================================================

for j = 1:7

subplot(3,8,16+j);

plot(t,log2(Mean_Fold_Change_Enzmye(Id11(14+j),:)),'-b','LineWidth',LW)

xlim([0,21]);

ylim(ylimit);

% xlabel('Time')

% ylabel('Rate constants')

set(gca,'fontsize',fs,'XTickLabel',{[]},'YTickLabel',{[]});

axh = gca;

set(axh,'XGrid','on','XTick',[0:5:20]);

hold on

end

end

**Code for our Regression to Determine Linear Trends for**

**Enzyme Activities from 0-3 and 3-20 Hours**

function [Coefficients] = Regression_Test

load('Experiment1');

Data.rate1 = Solution;

clear Solution;

count = zeros(1,20);

error_tolerance(1:5) = [0.08 0.15 0.3 0.4 0.15];

error_tolerance(6:10) = [0.5 0.4 0.5 0.5 0.5];

error_tolerance(11:15) = [0.4 0.4 0.4 0.3 0.5];

error_tolerance(16:20) = [0.4 0.4 0.5 0.5 0.8];

c = 0;

for i = 1:20

for j = 1:100

if exist(strcat('RatePW',num2str(i),'_',num2str(j),'.mat'),'file')

load(strcat('RatePW',num2str(i),'_',num2str(j),'.mat'));

c = c + 1;

FVAL(i,c) = fval;

if fval < error_tolerance(i)

count(1,i) = count(1,i) + 1;

else

end

else

end

end

c = 0;

end

%%

mean_SSopt = zeros(137,20);

for i = 1:20

for j = 1:200

if exist(strcat('RatePW',num2str(i),'_',num2str(j),'.mat'),'file')

load(strcat('RatePW',num2str(i),'_',num2str(j),'.mat'));

if fval < error_tolerance(i)

mean_SSopt(:,i) = mean_SSopt(:,i) + SSopt*count(1,i)^-1;

else

end

else

end

end

end

Id1 = [1,6,11,16,21,26,31,36,41,46,51,56]; % CerS DHC

Id2 = [61,65,69,73,77,81,85,89,93,97,101,105]; % CerS PHC

Id3 = [3,8,13,18,23,28,33,38,43,48,53,58]; % Dihydroceramidase

Id4 = [63,67,71,75,79,83,87,91,95,99,103,107]; % Phytoceramidase

Id5 = [5,10,15,20,25,30,35,40,45,50,55,60]; % IPC Synthase DHC

Id6 = [64,68,72,76,80,84,88,92,96,100,104,108]; % IPC Synthase PHC

Id7 = [2,7,12,17,22,27,32,37,42,47,52,57]; % IPCase DHC

Id8 = [62,66,70,74,78,82,86,90,94,98,102,106]; % IPCase PHC

Id9 = [4,9,14,19,24,29,34,39,44,49,54,59]; % Hydroxylase

Id10 = [111,114,110,113,115,116,109,112]; % DHS & PHS

Id11 = [117:124,125:130,136,137,131:135]; % Fatty acid elongation

SS = Data.rate1;

Mean_Fold_Change_Enzmye = mean_SSopt./ndgrid(SS,[1:20]);

LM(:,1) = zeros(137,1);

LM(:,2:21) = log2(Mean_Fold_Change_Enzmye);

tt1 = [0:1:3]';

tt2 = [3:1:20]';

tt3 = [0:1:20]';

p_value = zeros(137,3);

slope = zeros(137,3);

intercept = zeros(137,3);

for i = 1:137

lm3 = fitlm(tt1,LM(i,[tt1+1]'),'linear');

temp1 = anova(lm3);

p_value(i,1) = temp1(1,5);

temp_s1= lm3.Coefficients;

slope(i,1) = temp_s1(2,1);

temp_i1 = lm3.Coefficients;

intercept(i,1) = temp_i1(1,1);

lm20 = fitlm(tt2,LM(i,[tt2+1]'),'linear');

temp2 = anova(lm20);

p_value(i,2) = temp2(1,5);

temp_s2 = lm20.Coefficients;

slope(i,2) = temp_s2(2,1);

temp_i2 = lm20.Coefficients;

intercept(i,2) = temp_i2(1,1);

lmtot = fitlm(tt3,LM(i,[tt3+1]'),'linear');

temp3 = anova(lmtot);

p_value(i,3) = temp3(1,5);

temp_s3 = lmtot.Coefficients;

slope(i,3) = temp_s3(2,1);

temp_i3 = lmtot.Coefficients;

intercept(i,3) = temp_i3(1,1);

clear lm3;

clear lm20;

clear lmtot;

clear temp1;

clear temp2;

clear temp3;

end

Coefficients.Ceramide_Synthase_3h_p = p_value([Id1,Id2]',1);

Coefficients.Ceramide_Synthase_3h_slope = slope([Id1,Id2]',1);

Coefficients.Dihydroceramidase_3h_p = p_value([Id3]',1);

Coefficients.Dihydroceramidase_3h_slope = slope([Id3]',1);

Coefficients.Phytoceramidase_3h_p = p_value([Id4]',1);

Coefficients.Phytoceramidase_3h_slope = slope([Id4]',1);

Coefficients.IPC_Synthase_3h_p = p_value([Id5,Id6]',1);

Coefficients.IPC_Synthase_3h_slope = slope([Id5,Id6]',1);

Coefficients.IPCase_3h_p = p_value([Id7,Id8]',1);

Coefficients.IPCase_3h_slope = slope([Id7,Id8]',1);

Coefficients.Hydroxylase_3h_p = p_value([Id9]',1);

Coefficients.Hydroxylase_3h_slope = slope([Id9]',1);

Coefficients.Sph_3h_p = p_value([Id10]',1);

Coefficients.Sph_3h_slope = slope([Id10]',1);

Coefficients.FA_3h_p = p_value([Id11]',1);

Coefficients.FA_3h_slope = slope([Id11]',1);

Coefficients.Ceramide_Synthase_20h_p = p_value([Id1,Id2]',2);

Coefficients.Ceramide_Synthase_20h_slope = slope([Id1,Id2]',2);

Coefficients.Dihydroceramidase_20h_p = p_value([Id3]',2);

Coefficients.Dihydroceramidase_20h_slope = slope([Id3]',2);

Coefficients.Phytoceramidase_20h_p = p_value([Id4]',2);

Coefficients.Phytoceramidase_20h_slope = slope([Id4]',2);

Coefficients.IPC_Synthase_20h_p = p_value([Id5,Id6]',2);

Coefficients.IPC_Synthase_20h_slope = slope([Id5,Id6]',2);

Coefficients.IPCase_20h_p = p_value([Id7,Id8]',2);

Coefficients.IPCase_20h_slope = slope([Id7,Id8]',2);

Coefficients.Hydroxylase_20h_p = p_value([Id9]',2);

Coefficients.Hydroxylase_20h_slope = slope([Id9]',2);

Coefficients.Sph_20h_p = p_value([Id10]',2);

Coefficients.Sph_20h_slope = slope([Id10]',2);

Coefficients.FA_20h_p = p_value([Id11]',2);

Coefficients.FA_20h_slope = slope([Id11]',2);

Coefficients.Ceramide_Synthase_tot_p = p_value([Id1,Id2]',3);

Coefficients.Ceramide_Synthase_tot_slope = slope([Id1,Id2]',3);

Coefficients.Dihydroceramidase_tot_p = p_value([Id3]',3);

Coefficients.Dihydroceramidase_tot_slope = slope([Id3]',3);

Coefficients.Phytoceramidase_tot_p = p_value([Id4]',3);

Coefficients.Phytoceramidase_tot_slope = slope([Id4]',3);

Coefficients.IPC_Synthase_tot_p = p_value([Id5,Id6]',3);

Coefficients.IPC_Synthase_tot_slope = slope([Id5,Id6]',3);

Coefficients.IPCase_tot_p = p_value([Id7,Id8]',3);

Coefficients.IPCase_tot_slope = slope([Id7,Id8]',3);

Coefficients.Hydroxylase_tot_p = p_value([Id9]',3);

Coefficients.Hydroxylase_tot_slope = slope([Id9]',3);

Coefficients.Sph_tot_p = p_value([Id10]',3);

Coefficients.Sph_tot_slope = slope([Id10]',3);

Coefficients.FA_tot_p = p_value([Id11]',3);

Coefficients.FA_tot_slope = slope([Id11]',3);

Coefficient1 = [intercept(:,1) slope(:,1)];

Coefficient2 = [intercept(:,2) slope(:,2)];

Coefficient3 = [intercept(:,3) slope(:,3)];

% LM(:,1) = zeros(137,1);

% LM(:,2:21) = log2(Mean_Fold_Change_Enzmye);

%

% [a,b] = size(Mean_Fold_Change_Enzmye);

%

% t1(:,1) = ones(4,1);

% t1(:,2) = [0:1:3]';

%

% tt1 = t1(:,2);

%

% t2(:,1) = ones(18,1);

% t2(:,2) = [3:1:20]';

%

% tt2 = t2(:,2);

%

% for i = 1:a

%

% [coe1,coe_int1,ri1,ri_int1,stat1] = regress(LM(i,[tt1+1])',t1);

% [coe2,coe_int2,ri2,ri_int2,stat2] = regress(LM(i,[tt2])',t2);

%

% Coefficient1(i,:) = [coe1(1),coe1(2),stat1];

% Coefficient2(i,:) = [coe2(1),coe2(2),stat2];

%

% end

%

% %% p values

%

%

% p_value_Ceramide_Synthase(1,:,1) = Coefficient1([Id1(1:6)],5)'; p_value_Ceramide_Synthase(1,:,2) = Coefficient2([Id1(1:6)],5)';

% p_value_Ceramide_Synthase(2,:,1) = Coefficient1([Id1(7:12)],5)'; p_value_Ceramide_Synthase(2,:,2) = Coefficient2([Id1(7:12)],5)';

% p_value_Ceramide_Synthase(3,:,1) = Coefficient1([Id2(1:6)],5)'; p_value_Ceramide_Synthase(3,:,2) = Coefficient2([Id2(1:6)],5)';

% p_value_Ceramide_Synthase(4,:,1) = Coefficient1([Id2(7:12)],5)'; p_value_Ceramide_Synthase(4,:,2) = Coefficient2([Id2(7:12)],5)';

%

% p_value_Dihydroceramidase(1,:,1) = Coefficient1([Id3(1:6)],5)'; p_value_Dihydroceramidase(1,:,2) = Coefficient2([Id3(1:6)],5)';

% p_value_Dihydroceramidase(2,:,1) = Coefficient1([Id3(7:12)],5)'; p_value_Dihydroceramidase(2,:,2) = Coefficient2([Id3(7:12)],5)';

% p_value_Phytoceramidase(1,:,1) = Coefficient1([Id4(1:6)],5)'; p_value_Phytoceramidase(1,:,2) = Coefficient2([Id4(1:6)],5)';

% p_value_Phytoceramidase(2,:,1) = Coefficient1([Id4(7:12)],5)'; p_value_Phytoceramidase(2,:,2) = Coefficient2([Id4(7:12)],5)';

%

% p_value_IPC_Synthase(1,:,1) = Coefficient1([Id5(1:6)],5)'; p_value_IPC_Synthase(1,:,2) = Coefficient2([Id5(1:6)],5)';

% p_value_IPC_Synthase(2,:,1) = Coefficient1([Id5(7:12)],5)'; p_value_IPC_Synthase(2,:,2) = Coefficient2([Id5(7:12)],5)';

% p_value_IPC_Synthase(3,:,1) = Coefficient1([Id6(1:6)],5)'; p_value_IPC_Synthase(3,:,2) = Coefficient2([Id6(1:6)],5)';

% p_value_IPC_Synthase(4,:,1) = Coefficient1([Id6(7:12)],5)'; p_value_IPC_Synthase(4,:,2) = Coefficient2([Id6(7:12)],5)';

%

% p_value_IPCase(1,:,1) = Coefficient1([Id7(1:6)],5)'; p_value_IPCase(1,:,2) = Coefficient2([Id7(1:6)],5)';

% p_value_IPCase(2,:,1) = Coefficient1([Id7(7:12)],5)'; p_value_IPCase(2,:,2) = Coefficient2([Id7(7:12)],5)';

% p_value_IPCase(3,:,1) = Coefficient1([Id8(1:6)],5)'; p_value_IPCase(3,:,2) = Coefficient2([Id8(1:6)],5)';

% p_value_IPCase(4,:,1) = Coefficient1([Id8(7:12)],5)'; p_value_IPCase(4,:,2) = Coefficient2([Id8(7:12)],5)';

%

%

% p_value_Hydroxylase(1,:,1) = Coefficient1([Id9(1:6)],5)'; p_value_Hydroxylase(1,:,2) = Coefficient2([Id9(1:6)],5)';

% p_value_Hydroxylase(2,:,1) = Coefficient1([Id9(7:12)],5)'; p_value_Hydroxylase(2,:,2) = Coefficient2([Id9(7:12)],5)';

%

%

% p_value_Sph(1,:,1) = Coefficient1([Id10(1:4)],5)'; p_value_Sph(1,:,2) = Coefficient2([Id10(1:4)],5)';

% p_value_Sph(2,:,1) = Coefficient1([Id10(5:8)],5)'; p_value_Sph(2,:,2) = Coefficient2([Id10(5:8)],5)';

%

%

% p_value_FA(1,1:8,1) = Coefficient1([Id11(1:8)],5)'; p_value_FA(1,1:8,2) = Coefficient2([Id11(1:8)],5)';

% p_value_FA(2,1:6,1) = Coefficient1([Id11(9:14)],5)'; p_value_FA(2,1:6,2) = Coefficient2([Id11(9:14)],5)';

% p_value_FA(3,1:7,1) = Coefficient1([Id11(15:21)],5)'; p_value_FA(3,1:7,2) = Coefficient2([Id11(15:21)],5)';

%

%

% Data.p_value_Ceramide_Synthase = p_value_Ceramide_Synthase;

% Data.p_value_Dihydroceramidase = p_value_Dihydroceramidase;

% Data.p_value_Phytoceramidase = p_value_Phytoceramidase;

% Data.p_value_IPC_Synthase = p_value_IPC_Synthase;

% Data.p_value_IPCase = p_value_IPCase;

% Data.p_value_Hydroxylase = p_value_Hydroxylase;

% Data.p_value_Sph = p_value_Sph;

% Data.p_value_FA = p_value_FA;

%

%

% %% Linear Coefficients

%

%

% slope_Ceramide_Synthase(1,:,1) = Coefficient1([Id1(1:6)],2)'; slope_Ceramide_Synthase(1,:,2) = Coefficient2([Id1(1:6)],2)';

% slope_Ceramide_Synthase(2,:,1) = Coefficient1([Id1(7:12)],2)'; slope_Ceramide_Synthase(2,:,2) = Coefficient2([Id1(7:12)],2)';

% slope_Ceramide_Synthase(3,:,1) = Coefficient1([Id2(1:6)],2)'; slope_Ceramide_Synthase(3,:,2) = Coefficient2([Id2(1:6)],2)';

% slope_Ceramide_Synthase(4,:,1) = Coefficient1([Id2(7:12)],2)'; slope_Ceramide_Synthase(4,:,2) = Coefficient2([Id2(7:12)],2)';

%

% slope_Dihydroceramidase(1,:,1) = Coefficient1([Id3(1:6)],2)'; slope_Dihydroceramidase(1,:,2) = Coefficient2([Id3(1:6)],2)';

% slope_Dihydroceramidase(2,:,1) = Coefficient1([Id3(7:12)],2)'; slope_Dihydroceramidase(2,:,2) = Coefficient2([Id3(7:12)],2)';

% slope_Phytoceramidase(1,:,1) = Coefficient1([Id4(1:6)],2)'; slope_Phytoceramidase(1,:,2) = Coefficient2([Id4(1:6)],2)';

% slope_Phytoceramidase(2,:,1) = Coefficient1([Id4(7:12)],2)'; slope_Phytoceramidase(2,:,2) = Coefficient2([Id4(7:12)],2)';

%

% slope_IPC_Synthase(1,:,1) = Coefficient1([Id5(1:6)],2)'; slope_IPC_Synthase(1,:,2) = Coefficient2([Id5(1:6)],2)';

% slope_IPC_Synthase(2,:,1) = Coefficient1([Id5(7:12)],2)'; slope_IPC_Synthase(2,:,2) = Coefficient2([Id5(7:12)],2)';

% slope_IPC_Synthase(3,:,1) = Coefficient1([Id6(1:6)],2)'; slope_IPC_Synthase(3,:,2) = Coefficient2([Id6(1:6)],2)';

% slope_IPC_Synthase(4,:,1) = Coefficient1([Id6(7:12)],2)'; slope_IPC_Synthase(4,:,2) = Coefficient2([Id6(7:12)],2)';

%

% slope_IPCase(1,:,1) = Coefficient1([Id7(1:6)],2)'; slope_IPCase(1,:,2) = Coefficient2([Id7(1:6)],2)';

% slope_IPCase(2,:,1) = Coefficient1([Id7(7:12)],2)'; slope_IPCase(2,:,2) = Coefficient2([Id7(7:12)],2)';

% slope_IPCase(3,:,1) = Coefficient1([Id8(1:6)],2)'; slope_IPCase(3,:,2) = Coefficient2([Id8(1:6)],2)';

% slope_IPCase(4,:,1) = Coefficient1([Id8(7:12)],2)'; slope_IPCase(4,:,2) = Coefficient2([Id8(7:12)],2)';

%

%

% slope_Hydroxylase(1,:,1) = Coefficient1([Id9(1:6)],2)'; slope_Hydroxylase(1,:,2) = Coefficient2([Id9(1:6)],2)';

% slope_Hydroxylase(2,:,1) = Coefficient1([Id9(7:12)],2)'; slope_Hydroxylase(2,:,2) = Coefficient2([Id9(7:12)],2)';

%

%

% slope_Sph(1,:,1) = Coefficient1([Id10(1:4)],2)'; slope_Sph(1,:,2) = Coefficient2([Id10(1:4)],2)';

% slope_Sph(2,:,1) = Coefficient1([Id10(5:8)],2)'; slope_Sph(2,:,2) = Coefficient2([Id10(5:8)],2)';

%

%

% slope_FA(1,1:8,1) = Coefficient1([Id11(1:8)],2)'; slope_FA(1,1:8,2) = Coefficient2([Id11(1:8)],2)';

% slope_FA(2,1:6,1) = Coefficient1([Id11(9:14)],2)'; slope_FA(2,1:6,2) = Coefficient2([Id11(9:14)],2)';

% slope_FA(3,1:7,1) = Coefficient1([Id11(15:21)],2)'; slope_FA(3,1:7,2) = Coefficient2([Id11(15:21)],2)';

%

%

% Data.slope_Ceramide_Synthase = slope_Ceramide_Synthase;

% Data.slope_Dihydroceramidase = slope_Dihydroceramidase;

% Data.slope_Phytoceramidase = slope_Phytoceramidase;

% Data.slope_IPC_Synthase = slope_IPC_Synthase;

% Data.slope_IPCase = slope_IPCase;

% Data.slope_Hydroxylase = slope_Hydroxylase;

% Data.slope_Sph = slope_Sph;

% Data.slope_FA = slope_FA;

% plot

% tt = [0:1:20];

LW = 4;

fs = 18;

ylimit = [-3,3];

figure(7)

for j = 1:length(Id1)

subplot(2,6,j);

plot(tt1,Coefficient1(Id1(j),2)*tt1+Coefficient1(Id1(j),1),'-r','LineWidth',LW)

hold on

plot(tt2,Coefficient2(Id1(j),2)*tt2+Coefficient2(Id1(j),1),'-r','LineWidth',LW)

hold on

% plot(tt3,Coefficient3(Id1(j),2)*tt3+Coefficient3(Id1(j),1),'-k','LineWidth',LW-2)

xlim([0,21]);

ylim(ylimit);

set(gca,'fontsize',fs,'XTickLabel',{[]},'YTickLabel',{[]});

axh = gca;

set(axh,'XGrid','on','XTick',[0:5:20]);

hold on

end

figure(8)

for j = 1:length(Id2)

subplot(2,6,j);

plot(tt1,Coefficient1(Id2(j),2)*tt1+Coefficient1(Id2(j),1),'-r','LineWidth',LW)

hold on

plot(tt2,Coefficient2(Id2(j),2)*tt2+Coefficient2(Id2(j),1),'-r','LineWidth',LW)

hold on

% plot(tt3,Coefficient3(Id2(j),2)*tt3+Coefficient3(Id2(j),1),'-k','LineWidth',LW-2)

xlim([0,21]);

ylim(ylimit);

% xlabel('Time')

% ylabel('Rate constants')

set(gca,'fontsize',fs,'XTickLabel',{[]},'YTickLabel',{[]});

axh = gca;

set(axh,'XGrid','on','XTick',[0:5:20]);

hold on

end

%% Ceramidase

figure(9)

for j = 1:length(Id3)

subplot(2,6,j);

plot(tt1,Coefficient1(Id3(j),2)*tt1+Coefficient1(Id3(j),1),'-r','LineWidth',LW)

hold on

plot(tt2,Coefficient2(Id3(j),2)*tt2+Coefficient2(Id3(j),1),'-r','LineWidth',LW)

hold on

% plot(tt3,Coefficient3(Id3(j),2)*tt3+Coefficient3(Id3(j),1),'-k','LineWidth',LW-2)

xlim([0,21]);

ylim(ylimit);

% xlabel('Time')

% ylabel('Rate constants')

set(gca,'fontsize',fs,'XTickLabel',{[]},'YTickLabel',{[]});

axh = gca;

set(axh,'XGrid','on','XTick',[0:5:20]);

hold on

end

figure(10)

for j = 1:length(Id4)

subplot(2,6,j);

plot(tt1,Coefficient1(Id4(j),2)*tt1+Coefficient1(Id4(j),1),'-r','LineWidth',LW)

hold on

plot(tt2,Coefficient2(Id4(j),2)*tt2+Coefficient2(Id4(j),1),'-r','LineWidth',LW)

hold on

% plot(tt3,Coefficient3(Id4(j),2)*tt3+Coefficient3(Id4(j),1),'-k','LineWidth',LW-2)

xlim([0,21]);

ylim(ylimit);

% xlabel('Time')

% ylabel('Rate constants')

set(gca,'fontsize',fs,'XTickLabel',{[]},'YTickLabel',{[]});

axh = gca;

set(axh,'XGrid','on','XTick',[0:5:20]);

hold on

end

%% IPC Synthase

figure(11)

for j = 1:length(Id5)

subplot(2,6,j);

plot(tt1,Coefficient1(Id5(j),2)*tt1+Coefficient1(Id5(j),1),'-r','LineWidth',LW)

hold on

plot(tt2,Coefficient2(Id5(j),2)*tt2+Coefficient2(Id5(j),1),'-r','LineWidth',LW)

hold on

% plot(tt3,Coefficient3(Id5(j),2)*tt3+Coefficient3(Id5(j),1),'-k','LineWidth',LW-2)

xlim([0,21]);

ylim(ylimit);

% xlabel('Time')

% ylabel('Rate constants')

set(gca,'fontsize',fs,'XTickLabel',{[]},'YTickLabel',{[]});

axh = gca;

set(axh,'XGrid','on','XTick',[0:5:20]);

hold on

end

figure(12)

for j = 1:length(Id6)

subplot(2,6,j);

plot(tt1,Coefficient1(Id6(j),2)*tt1+Coefficient1(Id6(j),1),'-r','LineWidth',LW)

hold on

plot(tt2,Coefficient2(Id6(j),2)*tt2+Coefficient2(Id6(j),1),'-r','LineWidth',LW)

hold on

% plot(tt3,Coefficient3(Id6(j),2)*tt3+Coefficient3(Id6(j),1),'-k','LineWidth',LW-2)

xlim([0,21]);

ylim(ylimit);

% xlabel('Time')

% ylabel('Rate constants')

set(gca,'fontsize',fs,'XTickLabel',{[]},'YTickLabel',{[]});

axh = gca;

set(axh,'XGrid','on','XTick',[0:5:20]);

hold on

end

%% IPCase

figure(13)

for j = 1:length(Id7)

subplot(2,6,j);

plot(tt1,Coefficient1(Id7(j),2)*tt1+Coefficient1(Id7(j),1),'-r','LineWidth',LW)

hold on

plot(tt2,Coefficient2(Id7(j),2)*tt2+Coefficient2(Id7(j),1),'-r','LineWidth',LW)

hold on

% plot(tt3,Coefficient3(Id7(j),2)*tt3+Coefficient3(Id7(j),1),'-k','LineWidth',LW-2)

xlim([0,21]);

ylim(ylimit);

% xlabel('Time')

% ylabel('Rate constants')

set(gca,'fontsize',fs,'XTickLabel',{[]},'YTickLabel',{[]});

axh = gca;

set(axh,'XGrid','on','XTick',[0:5:20]);

hold on

end

figure(14)

for j = 1:length(Id8)

subplot(2,6,j);

plot(tt1,Coefficient1(Id8(j),2)*tt1+Coefficient1(Id8(j),1),'-r','LineWidth',LW)

hold on

plot(tt2,Coefficient2(Id8(j),2)*tt2+Coefficient2(Id8(j),1),'-r','LineWidth',LW)

hold on

% plot(tt3,Coefficient3(Id8(j),2)*tt3+Coefficient3(Id8(j),1),'-k','LineWidth',LW-2)

xlim([0,21]);

ylim(ylimit);

% xlabel('Time')

% ylabel('Rate constants')

set(gca,'fontsize',fs,'XTickLabel',{[]},'YTickLabel',{[]});

axh = gca;

set(axh,'XGrid','on','XTick',[0:5:20]);

hold on

end

%% Hydroxylase

figure(15)

for j = 1:length(Id9)

subplot(2,6,j);

plot(tt1,Coefficient1(Id9(j),2)*tt1+Coefficient1(Id9(j),1),'-r','LineWidth',LW)

hold on

plot(tt2,Coefficient2(Id9(j),2)*tt2+Coefficient2(Id9(j),1),'-r','LineWidth',LW)

hold on

% plot(tt3,Coefficient3(Id9(j),2)*tt3+Coefficient3(Id9(j),1),'-k','LineWidth',LW-2)

xlim([0,21]);

ylim(ylimit);

% xlabel('Time')

% ylabel('Rate constants')

set(gca,'fontsize',fs,'XTickLabel',{[]},'YTickLabel',{[]});

axh = gca;

set(axh,'XGrid','on','XTick',[0:5:20]);

hold on

end

%% Sphingosine

figure(16)

for j = 1:length(Id10)

subplot(2,4,j);

plot(tt1,Coefficient1(Id10(j),2)*tt1+Coefficient1(Id10(j),1),'-r','LineWidth',LW)

hold on

plot(tt2,Coefficient2(Id10(j),2)*tt2+Coefficient2(Id10(j),1),'-r','LineWidth',LW)

hold on

% plot(tt3,Coefficient3(Id10(j),2)*tt3+Coefficient3(Id10(j),1),'-k','LineWidth',LW-2)

xlim([0,21]);

ylim(ylimit);

% xlabel('Time')

% ylabel('Rate constants')

set(gca,'fontsize',fs,'XTickLabel',{[]},'YTickLabel',{[]});

axh = gca;

set(axh,'XGrid','on','XTick',[0:5:20]);

hold on

end

%% Fatty acyl CoAs concentration

figure(17)

for j = 1:8

subplot(3,8,j);

plot(tt1,Coefficient1(Id11(j),2)*tt1+Coefficient1(Id11(j),1),'-r','LineWidth',LW)

hold on

plot(tt2,Coefficient2(Id11(j),2)*tt2+Coefficient2(Id11(j),1),'-r','LineWidth',LW)

hold on

% plot(tt3,Coefficient3(Id11(j),2)*tt3+Coefficient3(Id11(j),1),'-k','LineWidth',LW-2)

xlim([0,21]);

ylim(ylimit);

% xlabel('Time')

% ylabel('Rate constants')

set(gca,'fontsize',fs,'XTickLabel',{[]},'YTickLabel',{[]});

axh = gca;

set(axh,'XGrid','on','XTick',[0:5:20]);

hold on

end

%%=========================================================================

for j = 1:6

subplot(3,8,8+j);

plot(tt1,Coefficient1(Id11(8+j),2)*tt1+Coefficient1(Id11(8+j),1),'-r','LineWidth',LW)

hold on

plot(tt2,Coefficient2(Id11(8+j),2)*tt2+Coefficient2(Id11(8+j),1),'-r','LineWidth',LW)

hold on

% plot(tt3,Coefficient3(Id11(8+j),2)*tt3+Coefficient3(Id11(8+j),1),'-k','LineWidth',LW-2)

xlim([0,21]);

ylim(ylimit);

% xlabel('Time')

% ylabel('Rate constants')

set(gca,'fontsize',fs,'XTickLabel',{[]},'YTickLabel',{[]});

axh = gca;

set(axh,'XGrid','on','XTick',[0:5:20]);

hold on

end

%%=========================================================================

for j = 1:7

subplot(3,8,16+j);

plot(tt1,Coefficient1(Id11(14+j),2)*tt1+Coefficient1(Id11(14+j),1),'-r','LineWidth',LW)

hold on

plot(tt2,Coefficient2(Id11(14+j),2)*tt2+Coefficient2(Id11(14+j),1),'-r','LineWidth',LW)

hold on

% plot(tt3,Coefficient3(Id11(14+j),2)*tt3+Coefficient3(Id11(14+j),1),'-k','LineWidth',LW-2)

xlim([0,21]);

ylim(ylimit);

% xlabel('Time')

% ylabel('Rate constants')

set(gca,'fontsize',fs,'XTickLabel',{[]},'YTickLabel',{[]});

axh = gca;

set(axh,'XGrid','on','XTick',[0:5:20]);

hold on

end

end
